# Endohedral Mixed Aggregates: Sodium Alkoxide Cages with Organic or Inorganic Central Anions and Variable Hull

**DOI:** 10.1002/chem.202100912

**Published:** 2021-05-28

**Authors:** Erkam Cebi, Jan Klett

**Affiliations:** ^1^ Department of Chemistry Johannes Gutenberg University Mainz Duesbergweg 10–14 55128 Mainz Germany

**Keywords:** aggregation, alkoxide, cage compounds, isotopic labeling, sodium

## Abstract

Alkali metal alkoxides are widely used in chemistry due to their Brønsted basic and nucleophilic properties. Potassium alkoxides assist alkyllithium in the metalation of hydrocarbons in Lochmann‐Schlosser‐bases. Both compounds form mixed aggregates, which enhance the thermal stability, solubility, and the basic reactivity of these mixtures. A very unusual spherical mixed alkoxy aggregate was discovered by Grützmacher et al., where a central dihydrogen phosphide anion is surrounded by a highly dynamic shell of thirteen sodium atoms and a hull of twelve *tert*‐butoxide groups. This structural motif can be reproduced by a reaction of trimethylsilyl compounds of methane, halogens, or pseudo‐halogens with excess sodium *tert*‐butoxide. A nucleophilic substitution releases the corresponding anion, which is then encapsulated by the sodium alkoxide units. The compounds are soluble in hydrocarbon solvents, enabling studies of solutions by high‐resolution NMR spectroscopy and IR/Raman studies of the crystalline materials.

## Graphical abstract

**Sodium alkoxide with a pinch of salt**: The in situ release of organic or inorganic anions from silyl compounds allows for their encapsulation in a sodium alkoxide shell. These spherical mixed aggregates incorporate anions ranging from the organic and basic methyl anion, halides, and pseudo‐halides to the nitrate anion. In this form, such anions can be studied as isolated entities unaffected by the influence of strongly coordinating solvents.

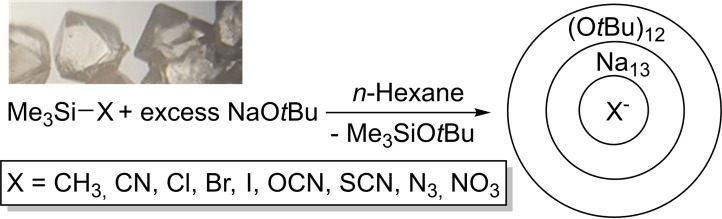

## Introduction

Alkali metal *tert*‐alkoxides are involved in a wide range of synthetic applications; however, despite its usefulness, they are hardly considered sophisticated reagents. Their roles in synthetic chemistry range from precursor for metal oxide layers,^[1]^ starting material for the Williamson ether synthesis,^[2]^ additive in polymerizations,^[3]^ or as moderate base^[4]^ in organic reactions. In some examples, alkali metal alkoxides play a less replaceable role when it is used as a catalyst in silylation reactions,^[5]^ in alkoxide assisted halogen metal exchanges,^[6]^ as an electron transfer reagent,^[7]^ or in organometallic bases of the Lochmann‐Schlosser type.^[8]^ These highly reactive bases are usually compiled from *n*‐butyllithium^[9]^ [Li*n*C_4_H_9_] and potassium *tert*‐butoxide [KO*t*Bu].^[10]^ When neopentyllithium^[11]^ [LiCH_2_ *t*Bu, LiNp] was used in combination with KO*t*Bu, the product of this mixture could be identified as mixed aggregates combining all four components, lithium, potassium, alkyl, and alkoxy groups side by side.^[12]^ A range of other alkyllithium compounds, such as methyllithium,^[13]^ *iso*‐butyllithium,^[14]^ or *tert*‐butyllithium,^[15]^ were tested in such mixtures with potassium alkoxide. Strikingly, no results exist about corresponding reactions and mixtures using silylalkyllithium compounds such as trimethylsilyl methyllithium^[16]^ [LiCH_2_SiMe_3_], despite its commercial availability. Other useful properties of the trimethylsilylmethyl group are based on the carbon‐silicon bond polarity and negative hyperconjugation^[17]^ resulting in better stabilization of the negative charge on the carbon atom^[18]^ (basicity:^[19]^ CH_2_ *t*Bu>CH_2_SiMe_3_), or advantageous crystallization behavior due to the group polarities.

## Results and Discussion

**Scheme 1:**
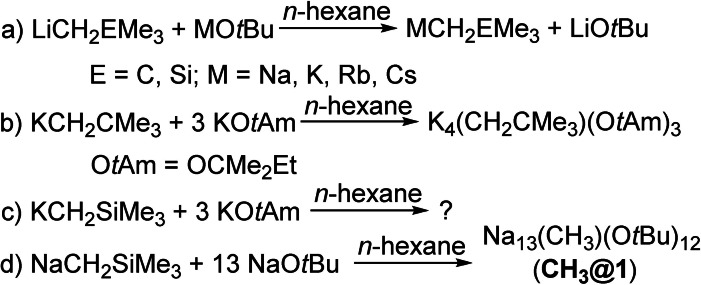
a) formation of heavier alkali metal compounds by reaction of alkyllithium with alkali metal alkoxide; b) formation of a potassium superbase from neopentyl potassium and potassium alkoxide; c) unsuccessful reaction of trimethylsilylmethyl potassium with potassium alkoxide; d) formation of **CH_3_@1** from trimethylsilylmethyl sodium with excess sodium alkoxide.

The lack of information regarding the use of trimethylsilylmethyl in alkali metal superbases is not to be confused with related reactions producing heavier alkali metal trimethylsilylmethyl congeners of sodium [NaCH_2_SiMe_3_],[^20^, ^21^] potassium [KCH_2_SiMe_3_],[^20^, ^22^] rubidium [RbCH_2_SiMe_3_],^[23]^ or cesium [CsCH_2_SiMe_3_]^[23]^ (Scheme 1). This approach is also applicable to neopentyl compounds of sodium^[24]^ and potassium.^[12]^ In our quest to obtain unimetal^[25]^ or homo‐metallic superbases, we were successful in producing neopentyl compounds such as [K_4_Np(O*t*Am)_3_] (O*t*Am=OCMe_2_Et).^[26]^ However, in preceding experiments with trimethylsilylmethyl potassium [KCH_2_SiMe_3_] and an excess of [KO*t*Bu] or [KO*t*Am], only intractable products were obtained. The use of the corresponding sodium compounds, NaCH_2_SiMe_3_ [NaR] and NaO*t*Bu, turned out to be more successful. Heating of solutions in *n*‐hexane or *n*‐heptane resulted in clear, yellowish solutions, which produced yellowish octahedral crystals of compound **CH_3_@1** (in *n*‐heptane, yield (NaO*t*Bu): 0.70 g, 60 %) with the composition [Na_13_(CH_3_)(O*t*Bu)_12_] (Scheme 1).

The same result was observed when LiCH_2_SiMe_3_ was treated with excess NaO*t*Bu. However, the purity is affected by the presence of side‐products. We chose the denomination of **CH_3_@1** to emphasize the design of the molecule: a spherical cage molecule with the possibility of accommodating a range of small atomic and molecular units in its center. In this regard, it is comparable to endohedral fullerenes^[27]^ [e. g., M@C_60_] (or alsosilafulleranes^[28]^), with which it also shares the shape derived from an icosahedron. Spherical but less homoleptic or symmetric host‐arrangements^[29]^ are a repeating topic in chemistry, also sometimes with incorporated anions^[30]^ or alkali metal atoms in disordered positions.^[31]^ The tendency of NaO*t*Bu to form spherical arrangements (but without central atom or molecule) is expressed in it hexameric^[1]^ or nonameric^[32]^ forms. Here a Na_2_O_2_ four‐ring and its extension are found as central structural motif, which can be described as ring‐laddering.^[33]^

**Figure 1:**
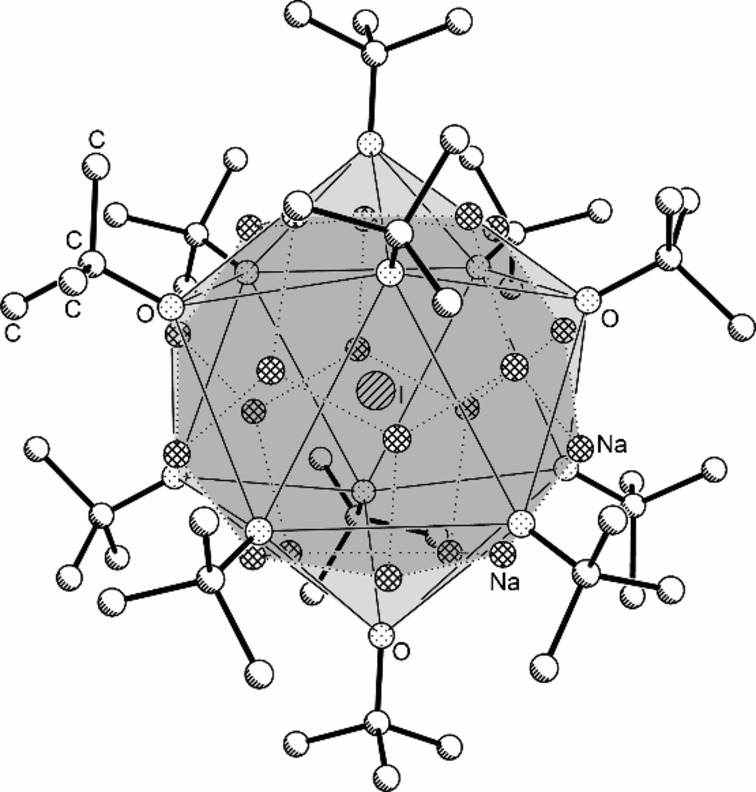
Molecular structure of compounds of the composition **X@1**, X=CH_3_, CN, Cl, Br, I, OCN, SCN, N_3_, or NO_3_. The structure of **I@1** is shown as a representative structure. Hydrogen atoms and minor‐disordered units are omitted and only representative atom names are shown for clarity. The dark gray shaded areas emphasize the pentagonal dodecahedron formed by sodium atoms; the light grey shaded areas emphasize the corners of the icosahedron formed by O*t*Bu oxygen atoms.

Strikingly, **CH_3_@1** is a close analogue of a predecessor compound reported about as **PH_2_@1** by Grützmacher et al. in 2010 as sodium alkoxide/dihydrogen phosphide complex with the formula Na_13_(PH_2_)(O*t*Bu)_12_.^[34]^ Both compounds crystallize in a cubic cell with cell constants *a* between 19 and 20 Å (**PH_2_@1**: 19.3622(3) Å at 30 K; **CH_3_@1**: 19.4734(21) Å at 173 K) with a face‐centered cube packing. The peripheral region of the spherical molecule is formed by a symmetrical arrangement of twelve *tert*‐butoxy groups in both cases (Figure 1). The inward pointing oxygen atoms are placed on the corners of an almost regular icosahedron. 13 positionally disordered sodium atoms are placed in good proximity on the 20 corners of a regular pentagon dodecahedron located inside of the oxygen icosahedron.

**Figure 2:**
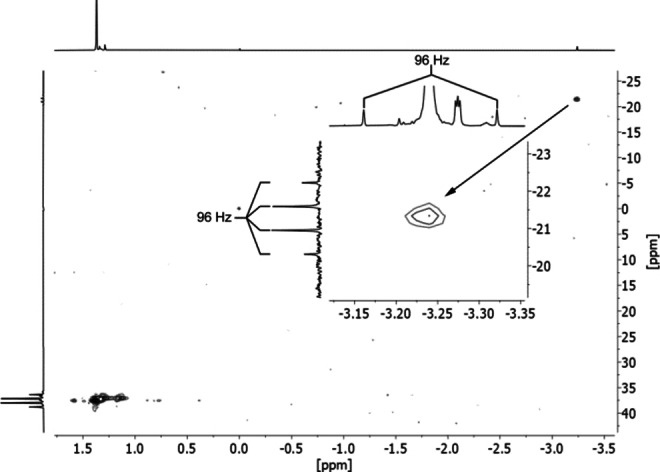
^1^H–^13^C HSQC NMR spectrum of **CH_3_@1** in C_6_D_6_, the traces of the 2D plot are separately recorded ^1^H (top) and ^13^C (left) NMR spectra. The inset shows a magnified view of the methide (CH_3_) resonance in the high‐field area with traces of the corresponding spectra. The methide signal clearly shows a 1 : 3 : 3 : 1 quartet structure with a coupling constant of 96 Hz in the 13 C NMR spectrum, consistent with a CH_3_ unit.

The 13 sodium atoms are statistically distributed over these 20 positions by a dynamic ion movement, which manifest itself as positional disorder in the molecular structure. The charges of the 12 alkoxide anions and the 13 sodium cations are balanced by the central anion. The shape of this molecule shows parallels to an inverted micelle, with nonpolar groups on the outside and highly polar or ionic units in the core.^[35]^ In the case of **PH_2_@1**, the center is formed by the dihydrogen phosphide anion [PH_2_^‐^], which is encapsulated by a cationic, spherical [Na_13_(O*t*Bu)_12_]^+^ aggregate. However, due to the profound disorder, the central anion appears as an indefinable atom. In addition, parallel to **PH_2_@1**, the positions of the attached hydrogen atoms in **CH_3_@1** cannot be determined. As the X‐ray crystallography supplies no definite information about the central anion, detailed spectroscopic information was consulted in this regard. On first inspection, the ^1^H NMR spectrum of solution of these crystals in deuterated cyclohexane [C_6_D_12_] revealed merely a singlet for the O*t*Bu protons. However, the signal is slightly shifted to lower field in respect to pure NaO*t*Bu in the same solvent (**1**, 1.29 ppm; NaO*t*Bu, 1.18 ppm). A similar shift is observed in solutions of deuterated benzene [C_6_D_6_] (**1**, 1.38 ppm; NaO*t*Bu, 1.29 ppm), which speaks against the presence of pure NaO*t*Bu. Closer inspection of the ^1^H NMR spectra revealed a second weak singlet (intensity ∼1 : 40) in the far high field (C_6_D_12_: −3.36 ppm; C_6_D_6_: −3.23 ppm). The non‐identity with NaO*t*Bu suggests that the *t*Bu signal and the high field signal belong to the same molecule. This data would be in accordance with a mixed sodium alkoxide/hydride aggregate. Comparable lithium aggregates were reported by Thomas et al., which were characterized by ^2^H and ^7^Li NMR spectroscopy as Li_10_H(O*t*Bu)_9_ (LiH ⋅ 9LiO*t*Bu) or Li_12_H(O*t*Bu)_11_ (LiH ⋅ 11LiO*t*Bu).^[36]^ However, the NMR experiments revealed ^13^C satellites with a coupling of 96 Hz at the high field signal and a cross peak in the HSQC spectrum (^1^H/^13^C, in C_6_D_12_: −3.36 ppm/−21.3 ppm; in C_6_D_6_: −3.23 ppm/−21.3 ppm). The presence of a methyl entity was revealed by a 1 : 3 : 3 : 1 quartet in a proton coupled ^13^C NMR spectrum (Figure 2).

This interpretation was substantiated by a positive‐phased signal in a ^13^C DEPT135 NMR experiment (with a pulse sequence optimized for ^1^J(^13^C,^1^H)=100 Hz), and the occurrence of CH_3_D^[37]^ after a destructive hydrolysis of **1** with D_2_O. The composition of **CH_3_@1** qualifies it as a proper alkyl/alkoxy mixed aggregate, though the endohedral position of the methide group should hamper its basicity.^[13]^ In fact, no solvolysis is observed in a ^1^H NMR of **CH_3_@1** in [D8]toluene.^[38]^ In contrast, a ^1^H NMR measurement in [D8]THF of **CH_3_@1** demonstrated its complete destruction by this coordinating solvent.

**Scheme 2:**
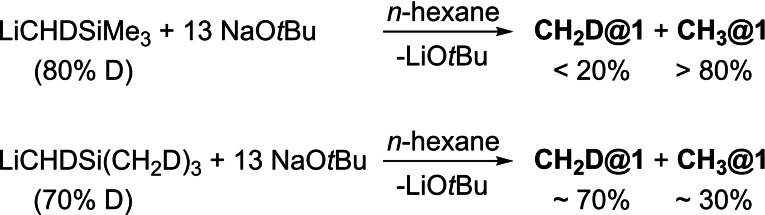
The outcomes of reactions of α‐deuterated LiCHDSiMe_3_ or α‐ and γ‐deuterated LiCHDSi(CH_2_D)_3_. In the first case the deuterium content of **CH_2_D@1** was depleted, the latter reaction led to a deuterium‐rich product.

A possible source of the methyl anion is the Me_3_SiCH_2_ group, the destruction of which would be in accordance with the lack of results of the corresponding Lochmann‐Schlosser chemistry. To test this hypothesis, we used α‐mono‐deuterated trimethylsilylmethyllithium [LiCHDSiMe_3_] (degree of deuteration of 80 %). However, after the reaction with excess NaO*t*Bu (yield: 0.27 g, 46 %), only small amounts of the mono‐deuterated **CH_2_D@1** (<20 %) beside **CH_3_@1** could be detected in the ^1^H NMR with secondary isotopic shift^[39]^ on nuclear shielding of ^2^ΔH(D)=+0.035 ppm. In contrast, when α‐ and γ‐deuterated trimethylsilyl‐methyllithium [LiCHDSi(CH_2_D)_3_] with 70 % deuteration was used, the degree of deuteration in **CH_2_D@1** was retained (Scheme 2).

These results clearly point towards the trimethylsilylmethyl group as source of the methyl anion in **CH_3_@1**. Accordingly, both α and γ positions are involved in this reaction. However, an isomerization of these positions by proton exchange following metalations might be involved. Experiments using bis(trimethylsilyl)methyl sodium^[40]^ confirmed these findings.

The observed ^1^J(^1^H‐^13^C) coupling constant of 96 Hz, which is comparable to the corresponding coupling in methyllithium [LiMe] with 98 Hz,^[41]^ suggests a low s‐orbital contribution of the carbon atom to the C−H bonds. The ^2^J(H−H) coupling of 10 Hz (derived from the corresponding ^2^J(H−D) coupling of 1.5 Hz) is lower than the value for methyllithium with 13 Hz.^[42]^ It is a remarkable fact that **CH_3_@1** offers the opportunity to study an alkali metal methide compound in solution by high‐resolution NMR spectroscopy, without the presence of a donor solvent. Carbon‐sodium interactions are considered predominantly ionic; the coordination of the central methide by 13 highly mobile sodium atoms in **CH_3_@1** makes the description as highly polar multi‐centered covalent interaction even less applicable.

The IR and Raman spectra of both **CH_3_@1** and **CD_3_@1** (the latter was obtained by reaction of fully deuterated trimethylsilyl methyllithium [LiCD_2_Si(CD_3_)_3_] with excess NaO*t*Bu; yield: 0.14 g, 36 %), show significant differences from NaO*t*Bu. However, no absorption band could be assigned to the corresponding vibrations of the CH_3_ or CD_3_ anions.

Neither spectroscopic data nor X‐ray diffractometry allow to draw valid conclusions about the conformation of the methyl anion. Regarding the low inversion barrier of the free methide anion^[43]^ and highly dynamic environment, it is reasonable to assume a fluxional unit.^[44]^

**Scheme 3:**
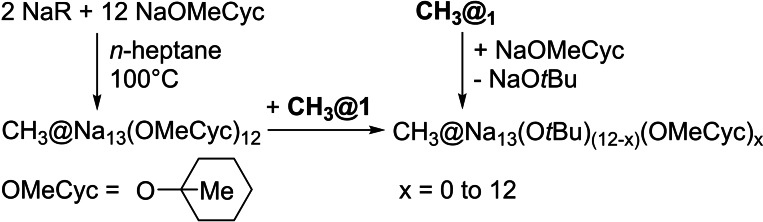
Synthesis of a methylcyclohexanolate (OMeCyc) analogue of **CH_3_@1** and alternative introduction of methylcyclohexanolate groups into the alkoxy shell of **CH_3_@1**.

Earlier attempts to produce **CH_3_@1** from sub‐ideal ratios of NaR and NaO*t*Bu (e. g., 1/4) regularly led to a second high field CH_3_ singlet signal of lower intensity in the ^1^H NMR spectrum at −3.25 ppm in C_6_D_12_ or −3.13 ppm in C_6_D_6_. When present, this signal is accompanied by two other singlets (in C_6_D_12_: −2.36 ppm and −0.03 ppm; in C_6_D_6_: −2.08 ppm and 0.37 ppm), similar to the signals of pure NaR^[20]^ (in C_6_D_12_: CH_2_: −2.23 ppm, SiMe_3_: 0.05 ppm; in C_6_D_6_: CH_2_: −2.44 ppm, SiMe_3_: 0.15 ppm). This result is consistent with the replacement of a peripheral O*t*Bu group by a CH_2_SiMe_3_ group, associated with a low field shift of the ^1^H NMR signal of the central methide anion. The possibility of another unknown central anion causing the lower field signal was ruled out by the addition of NaR to a pure sample of **CH_3_@1**, which intentionally replicated the signal pattern. The low solubility of NaR in alkanes prevents the addition of more than one R group to **CH_3_@1**. The partial or complete replacement of O*t*Bu groups in **CH_3_@1** was achieved by another sodium alkoxide, sodium 1‐methylcyclohexanolate^[45]^ [NaOMeCyc] (Scheme 3). The addition of NaOMeCyc to solutions of **CH_3_@1** transformed the original methide singlet in the ^1^H NMR by a new signal of apparently high‐multiplicity (e. g. nonet or higher), showing a striking resemblance to the ^2^H NMR signal of Li_10_H(O*t*Bu)_9_ and Li_12_H(O*t*Bu)_11_, caused by the coupling with ^6^Li.^[36]^ The individual peak distances change with the used solvent (in Hertz at 400 MHz: C_6_D_6_: 9.6 Hz; C_6_D_12_: 4.6 Hz), which is not consistent with a proton‐proton coupling but with a roughly proportional change of the chemical shift by a step‐by‐step exchange of O*t*Bu groups with OMeCyc groups in **CH_3_@1**.

**Figure 3:**
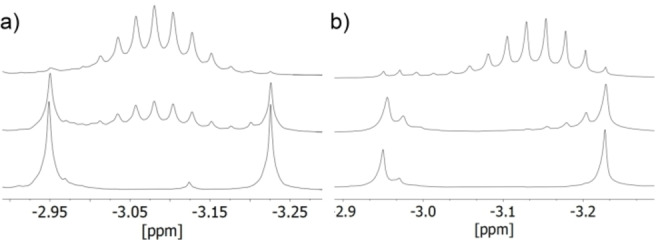
Evolution of a mixture of **CH_3_@1** and CH_3_@Na_13_(OMeCyc)_12_ in deuterated benzene observed by ^1^H NMR spectroscopy at 50 °C. The two initial singlet resonances are transformed into a group of roughly equidistant signals; from bottom to top: a) without additional NaO*t*Bu, 0 min, 39 min, 87 min; b) with additional NaO*t*Bu, 0 min, 60 min, 450 min.

[CH_3_@Na_13_(OMeCyc)_12_] is formed in analogy to **CH_3_@1** by a reaction of NaR with an excess of NaOMeCyc, as confirmed by ^1^H NMR spectroscopy (OMeCyc: 0.9–1.8 ppm). When this compound is mixed with **CH_3_@1** in C_6_D_6_, an exchange of alkoxy groups between these compounds can be observed by ^1^H NMR spectroscopy (Figure 3).

Up to eleven new signals appear at an elevated temperature (50 °C) between the two original methide ^1^H NMR signals at −2.95 ppm (CH_3_@Na_13_(OMeCyc)_12_) and −3.23 ppm (**CH_3_@1**) after several minutes. It is possible to assign these 13 roughly equidistant resonances to mixed alkoxy species of the formula CH_3_@Na_13_(O*t*Bu)_12‐x_(OMeCyc)_x_, with x=0 (**CH_3_@1**) to x=12 (CH_3_@Na_13_(OMeCyc)_12_). The intensities of the peaks, each representing several conformational isomers, follow a statistical Gauss contribution; the position of the maximum depends on the ratio of the starting materials. So far, it is not clear how changes in the alkoxy shell of **CH_3_@1** influence the NMR chemical shift of the methide group. Possible influential properties are the molecular weight of participating groups, group basicity, and/or changes in interactions with the sodium cations affecting their dynamic movement. However, the gradual change in the composition of alkoxy sphere in **CH_3_@1** resulting in a proportional change of the chemical shift of the methide anion is consistent with its central position inside the spherical arrangement consisting of twelve alkoxide units. To test whether the exchange of alkoxide group proceeds through a dissociative or associative process, additional NaO*t*Bu was added to freshly produced mixtures of **CH_3_@1** and CH_3_@Na_13_(OMeCyc)_12_, which were supposed to accelerate the reaction in case of an associative path. Beside a significant deceleration of the alkoxide‐exchange, changes in the evolution of the signal patterns are also observed (Figure 3a). Without the addition of extra alkoxide, the new signals appear directly in the middle between to original signals, while the addition of the alkoxide leads to new signals appearing close to the original signals. In the former case the instant exchange of multiple alkoxide groups takes place, directly producing species with similar numbers of both alkoxides and seemingly the product of a temporary fusion. The addition of extra alkoxide favors a step‐by‐step exchange of single alkoxide units, slowly enriching the two original species with more of these units (Figure 3b).

As the source of the methide anion is the trimethylsilylmethyl group [R], its formation requires the cleavage of a C−Si bond^[46]^ (nucleophilic demethylation). A possible mechanism is the elimination of methyl sodium^[47]^ from trimethylsilylmethylsodium [NaR], leaving behind a dimethylsilaethylene,^[48]^ which undergoes dimerization or polymerization.^[49]^ An alternative route is a nucleophilic attack of an alkoxy group on tetramethylsilane, which is formed by hydrolysis of NaR. The use of functionalized trimethylsilyl compounds [Me_3_SiX] in reactions with excess NaO*t*Bu would demonstrate that other anionic units can be released in situ by nucleophilic substitution^[50]^ and trapped in mixed aggregates [**X@1**] as well. In this case spherical mixed aggregates emerge as ubiquitous structural motifs for the encapsulation of small organic and inorganic anions.

To monitor the occurrence of this reaction by NMR spectroscopy, we used ^13^C‐enriched trimethylsilylcyanide [Me_3_Si^13^CN] in a reaction with excess NaO*t*Bu in *n*‐hexane; heating resulted in a clear solution, which afforded colorless octahedral crystals of ^**13**^ **CN@1** (yield: 0.34 g, 56 %). X‐ray diffractometry revealed a cubic cell with the cell constant very close to **CH_3_@1** and **PH_2_@1** (a=19.430(3) Å, 173 K, Table 1).

**Table 1:** Cell parameters of the cubic cells of compounds **X@1**, X=CH_3_, Cl, Br, I, OCN, SCN, N_3_, NO_3_, as determined by X‐ray crystallography at 173 K.

Compound	Measured cell parameters		Central anion X	
	*a* [Å]	*V* [Å^3^]	Size [Å]	*V*^[a]^ [Å^3^]
**CH_3_@1**	19.4734(21)	7384.5(14)		26.7
**Cl@1**	19.4387(12)	7345.2(8)	3.34^[b]^	22.4
**Br@1**	19.4715(15)	7382.4(10)	3.64^[b]^	26.5
**I@1**	19.5296(10)	7448.7(6)	4.12^[b]^	32.5
**CN@1**	19.430(3)	7334.9(21)		27.3
**OCN@1**	19.3796(12)	7278.3(8)		33.4
**SCN@1**	19.4555(12)	7364.2(8)		43.7
**N_3_@1**	19.3517(16)	7246.9(10)		32.3
**NO_3_@1**	19.5148(5)	7431.8(3)		40.5

[a] Calculated volumes, see Supporting Information for details. [b] Anion sizes from ref. [55] (doubled radii).

In analogy to **CH_3_@1** and **PH_2_@1**, the structure reveals a disorderly icosahedral arrangement of O*t*Bu groups and thirteen sodium atoms on twenty positions. The central electron density can be interpreted as diatomic unit, but the profound disorder of the molecule prevents a reliable assignment. ^1^H NMR in C_6_D_6_ revealed an O*t*Bu singlet at 1.39 ppm. The corresponding ^13^C NMR spectrum shows a resonance at 164.7 ppm for the CN group beside the two O*t*Bu signals at 37.7 (OC*Me*_3_) and 66.6 ppm (O*C*Me_3_). The ^13^C resonance of ^13^CN is very similar to the corresponding sodium salt in D_2_O (164.8 ppm).^[51]^ Crystals of the natural isotope compound **CN@1** (yield: 0.81 g, 67 %) were obtained using the same protocol as for ^**13**^ **CN@1**. The IR and Raman spectra^[52]^ of **CN@1** and ^**13**^ **CN@1** are almost identical to **CH_3_@1**. However, additional bands of the C≡N triple bond are observed at 2079 cm^−1^ (**CN@1**) or at 2036 cm^−1^ (^13^ **CN@1**), respectively.^[53]^ These results are consistent with the calculated stretching frequencies of the C≡N triple bond including the respective isotope, and are in agreement with the corresponding value of solid sodium cyanide with 2091 cm^−1^.^[54]^

**Scheme 4:**
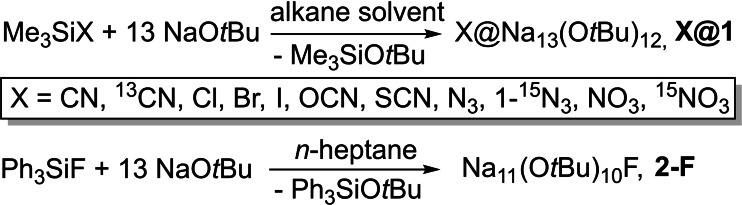
Synthesis of **X@1** and **2‐F** by reaction of functionalized trimethylsilyl compounds with excess NaO*t*Bu. Trimethylsilylcyanide, trimethylsilylazide, and trimethylsilyl nitrate were also used enriched with ^13^C and ^15^N, respectively.

**Figure 4:**
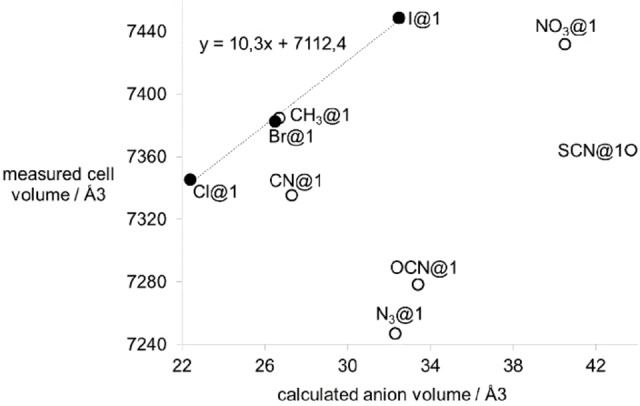
Graphical representation of the measured cell volumes of the compounds **X@1** and the calculated volumes of the corresponding anions. The halide compounds are shown as full circles with a fitted line (determination coefficient 0.9972), all other compounds as open circles.

Other trimethylsilyl compounds of inorganic ions were tested in similar reactions. However, the halides as obvious candidates do not allow the definite characterization of **X@1** (X=F, Cl,^[28]^ Br, I) by IR/Raman spectroscopy due to the absence of inherent vibration. NMR spectroscopy is a better option: all four nuclei are NMR responsive. While ^19^F NMR is a routine method in organic and inorganic chemistry, the heavier nuclei, ^35^Cl, ^79^Br, and ^127^I are rarely used in NMR spectroscopy. This is primarily due to the quadrupolar properties of these nuclei, which prevent sharp signals if the corresponding nucleus is not situated in a highly symmetric environment. The preparation of **Cl@1**, **Br@1**, and **I@1** was straight forward, following the procedure used for the synthesis of **CN@1** (Scheme 4), producing colorless octahedral crystals (yields: **Cl@1**, 0.87 g, 72 %; **Br@1**, 0.72 g, 58 %; **I@1**, 0.73 g, 56 %). X‐ray crystallography revealed cubic cells with similar cell constants compared to **PH_2_@1**, **CH_3_@1**, and **CN@1**. Plotting the corresponding cell volumes against the calculated anion‐volumes (Figure 4), places the resulting points for **Cl@1**, **Br@1**, and **I@1** close to a fitted line consistent with a proportional increase of the molecule size. The structure of **I@1** as the qualitative best structure is shown representatively in Figure 1. The heavy central iodide is not affected by the disorder, which leads to a comparatively good quality of the structure solution (*R*1=0.0602 with *I*>*2σ*).

**Figure 5:**
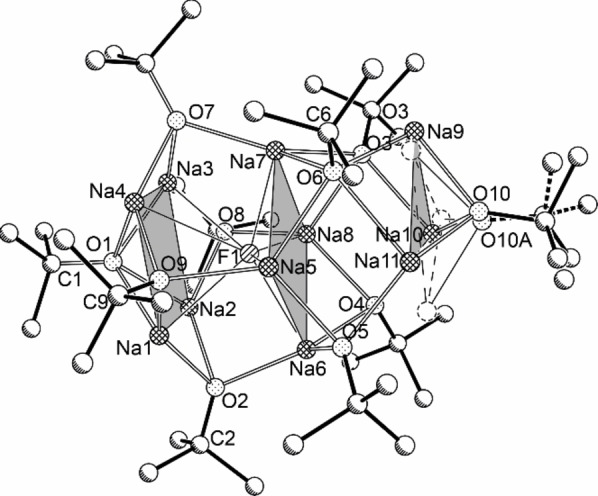
Molecular Structure of compound **2‐F**. Selected minor disordered units and hydrogen atoms are omitted for clarity. The two shaded areas emphasize the two squares and the triangles formed by sodium atoms. The sodium atoms of the sodium triangles are disordered, the alternative positions and the resulting triangle are shown by dotted circles and dashed lines.

The synthesis to obtain a fluoride compound was not successful in the case of Me_3_SiF. When Ph_3_SiF was used instead, large colorless blocks were obtained. X‐ray crystallography revealed the product as Na_11_F(O*t*Bu)_10_, **2‐F** (Figure 5, yield: 0.59 g, 60 %)

Compound **2‐F**, which crystallizes in the orthorhombic space group *Pbca*, is a structural analogue of a sodium alkoxide/hydroxide mixed aggregated reported by Grützmacher, Na_11_(OH)(O*t*Bu)_10_, **2‐OH** (space group *Pca2_1_*).^[56]^ **2‐OH** is described as a fusion of a [4.4.4.4] fenestrane, centered with the hydroxide anion and a distorted hexaprismane. For compound **2‐F**, a modified description is suggested: **2‐F** and **2‐OH** share a square antiprismatic arrangement of eight sodium atoms; one of the square faces (Na1, Na2, Na3, Na4) is μ_4_‐capped and four of the eight triangular faces are μ_3_‐capped by four oxygen atoms of O*t*Bu groups. The fluoride anion is situated inside the square antiprism close to the central square face (Na5, Na6, Na7, Na8; Na‐F 2.3300(9) −2.4399(9) Å), which is also part of a virtual distorted Na_8_‐cube, with the five residual faces μ_4_‐capped by the oxygen atoms of O*t*Bu groups. However, the positions of two of the peripheral four sodium atoms are generated by a positional disorder of Na11, which also affects the position of the two other sodium atoms (Na9 and Na10) of the same face. In fact, two Na_3_ triangles are superimposed (approximately occupation: 70/30 %), presenting a distorted Na_4_ trapezoid. Although the 11 sodium atoms are not dynamically distributed over 12 positions, compound **2‐F** gives an impression of how the formal addition of two additional NaO*t*Bu units and the replacement of fluoride by a larger and softer anion X^−^ leads to the transformation of the O*t*Bu square antiprism into a pentagonal antiprism of an icosahedron, resulting in the formation of the spherical and highly dynamic arrangement of **X@1**.

Similar to the compounds discussed before, **2‐F**, **Cl@1**, **Br@1**, and **I@1** are sufficiently soluble in C_6_D_6_ to allow their characterization by NMR spectroscopy. The ^1^H NMR spectra exhibited only singlet signals for the O*t*Bu protons (**2‐F**: 1.34 ppm, **Cl@1**: 1.39 ppm, **Br@1**: 1.39 ppm, and **I@1**: 1.38 ppm). The NMR spectra of the quadrupolar halides (^35^Cl, ^79^Br, ^127^I) yielded sharp resonances with linewidths comparable to the signals of the references in D_2_O (Na^35^Cl, Na^79^Br, K^127^I). This indicates a highly symmetric environment of the halide ions in respect to the NMR time‐scale, which is in accordance with a highly dynamic movement of the 13 sodium atoms sharing 20 positions. The chemical shift of **2‐F** in the ^19^F NMR with −223.3 ppm is very similar to NaF^[57]^ in solid state (−224.2 ppm; reference: CFCl_3_). In contrast, the signals of the heavier halide compounds are shifted considerably to the high field region (**Cl@1**: −90.7 ppm, **Br@1**: −141.4 ppm, **I@1**: −124.9 ppm) compared to the solid‐state MAS NMR spectra of the corresponding sodium salts (Na^35^Cl: −41.1 ppm; Na^79^Br: 1.29 ppm; Na^127^I: 226.7 ppm). These findings suggest that the differences in the structures of **2‐F** and **Cl@1**, **Br@1**, and **I@1** continue to exist in soluble phase.

Halide anions as central anions surrounded by a distorted spherical sodium/europium alkoxide aggregate^[58]^ (X=Cl) or a planar sodium amide “inverse crown”^[59]^ (with central halide X=Cl, Br, I, or central oxide^[60]^) have been reported before as structurally well‐defined compounds; neither of these examples showed dynamic movement of the sodium atoms.

The same protocol, which was successful in producing cyanide and halide compounds of **@1**, is also applicable to produce the pseudohalide compounds **OCN@1**, **SCN@1**, and **N_3_@1** (Scheme 4). The resulting octahedral crystals (yields: **OCN@1**, 0.84 g, 69 %; **SCN@1**, 0.58 g, 47 %; **N_3_@1**, 0.86 g, 71 %) were studied by NMR and IR/Raman spectroscopy. All three compounds produced singlet O*t*Bu resonances in the ^1^H NMR spectra (**OCN@1**: 1.37 ppm; **SCN@1**: 1.35 ppm; **N_3_@1**: 1.35 ppm). The three linear three‐atomic units showed characteristic bands in the vibrational spectra (ia: inactive; not observed; IR/Raman: **OCN@1**: ν_as_ 2197/2200 cm^−1^; ν_s_ 1303/1304 cm^−1^; δ 627/‐ cm^−1^; **SCN@1**: ν_as_ 2094/2096 cm^−1^; ν_s_ 781/781 cm^−1^; δ not observed; **N_3_@1**: ν_as_ 2078/ia cm^−1^; ν_s_ ia/1351 cm^−1^; δ 639/ia cm^−1^), which are in good accordance with the spectra of the corresponding sodium salts.[^61^, ^62^] By replacing Me_3_SiN_3_ with Me_3_Si(1‐^15^N_3_) as primary material, it was possible to obtain the terminally ^15^N‐enriched compound **1‐^15^N_3_@1** (yield: 0.43 g, 70 %). The vibrational spectra of **1‐^15^N_3_@1** confirmed the presence of an azide anion by a bathochromic shift of the corresponding bands (IR/Raman: ν_as_ 2066/ia cm^−1^; ν_s_ ia/1331 cm^−1^; δ 634/ia cm^−1^).

As it became clear that large, linear anions such as thiocyanate, or chemically reactive anions such as azide can be accommodated by spherical mixed aggregates, the question was raised if even larger and less inert anions are candidates for inclusion in such aggregates. The nitrate anion with its planar four‐atomic arrangement and its oxidizing potential offers interesting additional properties. Trimethylsilyl nitrate^[63]^ [Me_3_SiNO_3_] is available by a reaction of silver nitrate [AgNO_3_] with trimethylsilyl chloride [Me_3_SiCl].^[64]^ The reaction of Me_3_SiNO_3_ with excess NaO*t*Bu produced colorless octahedral crystals of **NO_3_@1** (yield: 0.31 g, 28 %). C_6_D_6_ solutions of these crystal showed single resonances at 1.36 ppm in the ^1^H NMR spectrum (O*t*Bu). The central nitrate ion is identified by characteristic bands in the vibrational spectra (IR: ν_as_ 1401 cm^−1^; γ 834 cm^−1^; Raman: ν_s_ 1076 cm^−1^).[^61^, ^65^] The origin of these bands was confirmed by IR/Raman measurements of ^**15**^ **NO_3_@1**. The corresponding trimethylsilyl nitrate [Me_3_Si^15^NO_3_] was accessible by a reaction of ^15^N‐enriched ammonium ^15^N‐nitrate with bis(trimethylsilyl) sulfate.^[66]^ The ^15^N NMR signal of ^**15**^ **NO_3_@1** could be detected as a singlet resonance at −6.8 ppm.^[67]^ Isotopic substitution led to bathochromic shifts in the IR spectrum (ν_as_ 1369 cm^−1^; γ 815 cm^−1^); the symmetric ν_s_ vibration visible in the Raman spectrum at 1076 cm^−1^ is not affected.

With only some examples of anions encapsulates by a spherical cationic [Na_13_(O*t*Bu)_12_]^+^ unit (**1^+^**), it is premature to speculate about systematic influences on the properties of the corresponding anions. Possible effects of the highly dynamic Na_13_ shell on the electronic properties (e. g. comparable to spherical ring currents)^[68]^ of the anions reflected in the chemical shift observed by NMR spectroscopy cannot yet be specified. This is further complicated by a lack of corresponding data in literature (e. g., solid state NMR spectra of sodium salts of pseudo halides). However, the NMR data of the methide anion in **CH_3_@1** (^1^H and ^13^C), and the halide anions in **Cl@1**, **Br@1**, and **I@1** show characteristic high‐fields shifts of the corresponding resonances. Remarkably, the ^35^Cl, ^79^Br, and ^127^I NMR data suggests that the environment of the halide anions is highly symmetric in the NMR time‐scale, as it is reflected by the sharp singlet resonances of these quadrupolar nuclei allowing their easy detection by NMR spectroscopy. Nevertheless, the observed change of the ^1^H chemical shift following changes of the sodium alkoxide capsule demonstrates its significance for interactions of the central ion with the outside. The high sodium cation mobility is a consequence of the energetic equivalence of the 20 possible positions and low activation barriers between them. Compound **2‐F**, which is structurally well defined and considerably less disordered, also exhibits 12 possible positions for 11 localized sodium cations, resulting in the disorder of four sodium cations over three positions. Further studies will have to show whether structural vibrations/deformations of the alkoxy group arrangement support this dynamic behavior in **X@1**.

The impact of the encapsulation on the vibrational behavior of the central anions in **PH_2_@1** and **CH_3_@1** can be expected to be induced by the interaction with the highly dynamic environment of the sodium cations, too. The dynamic environment possibly hinders the detection of C−H bonds in **CH_3_@1** by IR/Raman spectroscopy by massive broadening of the corresponding bands. The vibrations of heavier atoms are affected in a much smaller scale. In this respect parallels to the corresponding sodium salts in solid or molten state can be drawn. The vicinity of equidistant 13 sodium cations (in averaged 20 positions) should result in a pulling or pressing effect onto the central anionic molecule, depending on the size (longest axis or diameter). However, the vibrations of the central anions observed by IR or Raman spectroscopy are very similar to those of sodium salts, solutions, or gas phase theoretical calculations of the corresponding anions. The assumption of a flexible Na_13_(OtBu)_12_^+^ capsule, which is adaptable to an ellipsoid deformation, would be in line with these observations. This deformation would also explain, why the introduction of larger linear anions has only a minor effect on the corresponding cell parameters of the compounds (Table 1, Figure 4). In the case of the halide compounds **Cl@1**, **Br@1**, and **I@1**, the plotted data of measured cell volumes and calculated anion volumes indicate a proportional dependence.

The flexibility and adaptability of the sodium alkoxide shell towards the encapsulated anion also play a pivotal role in the process of its trapping. The hydroxide or fluoride anions in compound **2‐OH** or **2‐F** are seemingly too small and/or too hard to allow an arrangement such as **X@1**. Both anions prefer smaller units, resulting in a lower coordination number of eight (anion/sodium cations). For anions such as chloride or larger, the electrostatic attraction of the ions is weak enough and the Pauli repulsion between the atomic cores is strong enough to allow an arrangement with a high coordination number (13, or 20 with an occupation of 65 %). Calculations involving point charges reveal that the electrostatic potential inside an averaged spherical arrangement similar to **1^+^** is comparably flat over a large diameter. This observation can be traced back to an additive overlay of the individual charges at the center of the structure. This eventually leads to a weaker increase of attractive forces when the central ion is shifted away from the center.

## Conclusion

The evolution of the original phosphide compound **PH_3_@1** by Grützmacher et al. into a whole family of compounds **X@1** was achieved by replacing the central ion with a range of halide anions, pseudohalide anions, methide, or nitrate. The in situ release of the corresponding anion from a silyl compound enable its trapping by an excess of sodium alkoxide. The presented examples range from strongly reducing and highly basic anions (methide) to anions of strong acids with oxidizing properties (nitrate). The number of atoms in the anionic center ranges from one (halide) up to four (methide and nitrate). If the size of the anion falls below the size of chloride, the mixed alkoxide aggregates adapt a different, well defined arrangement of the formula Na_11_X(O*t*Bu)_10_ (X=OH: **2‐OH**, and X=F: **2‐F**).

The relevance of alkoxide compounds of type **X@1** arises not solely from the fact that it is possibly present in small amounts in many reported syntheses using sodium alkoxide, without having been detected or recognized. This can include the use of sodium alkoxides for the synthesis of alkyl or silyl ethers, the formation of metal alkoxides from the corresponding halides, or the formation of the sodium alkyl compounds starting from alkyllithium and sodium alkoxides. In addition, the encapsulation can lead to an unintentional masking of anions on one hand or their unintended introduction into reaction mixtures on the other hand. The awareness of these effects may help to understand undesired reaction outcomes and to improve corresponding synthetic procedures. The results concerning **CH_3_@1** also explain the lack of Lochmann‐Schlosser superbase chemistry of the otherwise very useful and versatile trimethylsilylmethyl group.

The vagueness and lack of crystallographic accuracy of this class of alkali metal alkoxide compounds are richly compensated by several singular properties, which are rarely seen in this combination. The inverse‐micellar arrangement encapsulates anions in a way that enables their solubilization in non‐polar media; the release of the anion can be achieved in highly polar solvents such as THF. Furthermore, the encapsulation enables the study of such otherwise insoluble anions in solution by high resolution NMR or UV/Vis spectroscopy. This option is even more intriguing, when the in situ isolation and characterization of highly reactive and otherwise not isolable anions is rendered possible by this approach. However, the influence of the highly dynamic capsule on the spectroscopic properties is not yet fully understood.

The modification of both the central ion and the peripheral alkoxy groups offers the opportunity to evolve the spherical entities into larger structures, retaining the high sodium cation mobility for ion‐conducting applications. The replacement of the peripheral alkoxy groups by other available groups will introduce new properties, such as redox‐activity, or super‐basicity when organic alkyl groups are introduced.

## Experimental Section

**Crystallographic data**: Deposition numbers 2061407, 2061408, 2061409, 2061410, 2061411, and 2061412 contain the supplementary crystallographic data for this paper. These data are provided free of charge by the joint Cambridge Crystallographic Data Centre and Fachinformationszentrum Karlsruhe Access Structures service.

## Conflict of interest

The authors declare no conflict of interest.

## Supporting information

As a service to our authors and readers, this journal provides supporting information supplied by the authors. Such materials are peer reviewed and may be re‐organized for online delivery, but are not copy‐edited or typeset. Technical support issues arising from supporting information (other than missing files) should be addressed to the authors.

Supporting Information
