# Supplementary material for: Endohedral Mixed Aggregates: Sodium Alkoxide Cages with Organic or Inorganic Central Anions and Variable Hull
Source: Chemistry. 2021 May 28;27(49):12693–701. doi: 10.1002/chem.202100912 (PMC8453731; doi:10.1002/chem.202100912)
Supplement: Supplementary file 1 — Supporting Information [file CHEM-27-12693-s001.pdf]

Chemistry–A European Journal

Supporting Information

Endohedral Mixed Aggregates: Sodium Alkoxide Cages with Organic or Inorganic Central Anions and Variable Hull

Erkam Cebi and Jan Klett*

Table of contents:

**General Methods**	2
**Synthesis** of compounds **X@1**, **2-F**, and selected starting materials	2-7
**Table S1**: General syntheses of compounds **X@1**	7
**Crystallographic measurements**	
**Table S2**: Crystallographic data and refinement for compounds **X@1**, **2-F**	8
**Table S3**: Cell measurements of compounds **X@1** at 173 K	9
**NMR spectroscopy**	10-36
**Table S4**: NMR spectroscopic data of compounds **X@1**	10
**IR/Raman spectroscopy**	37-57
**Table S5-1**: General IR spectroscopic data of compounds **X@1** and **2-F**	56
**Table S5-2**: General Raman spectroscopic data of compounds **X@1** and **2-F**	56
**Table S5-3**: IR and Raman spectroscopic data of central anions of compounds **X@1**	57
**Theoretical measurements of anions sizes**	58
**Table S6**: Measured cell volumes of compounds **X@1** and calculated anion sizes	58
**Electrostatical considerations**	59-60
**References**	61

General Methods.

n-Hexane, *n*-heptane, and deuterated solvents were dried with potassium and distilled. All synthetic work was carried out under an inert argon atmosphere using standard Schlenk and glove-box techniques. Sodium *t*-butoxide was purchased from Sigma-Aldrich and sublimed in vacuum before use, $K^{13}CN$ and $Na(1\text{-}^{15}N_3)$ were purchased from Deutero GmbH, $NH_4{}^{15}NO_3$ was purchased from ISOTEC/Sigma-Aldrich. $LiCH_2SiMe_3$ was purchased from Sigma-Aldrich, $NaCH_2SiMe_3$ was prepared following literature procedure,[1] using *n*-hexane as solvent.

^{1}H, ^{2}H, ^{7}Li, ^{13}C, ^{15}N, ^{19}F, ^{35}Cl, ^{79}Br, ^{127}I, COSY, and HSQC NMR spectra were recorded on a Bruker AV 400 spectrometer, or on a Bruker Avance III 600 spectrometer. All spectra were referenced to the resonances of the deuterated solvent used or to reference samples, respectively.

Infrared spectra were recorded on a BioRad Excalibur FTS 3100 spectrometer and a Varian 3100 FT-IR Excalibur Series with nujol mulls between CsBr plates, a Bruker Alpha FT-IR spectrometer with ATR unit containing a diamond crystal in the inert atmosphere of a glove box. Raman spectra were measured on a Nicolet 5700 FT-IR spectrometer combined with NXR 9650 FT-Raman Module equipped with a 1064 nm laser, a Microstage Microscope, and a NXR Genie Ge-detector using single crystals in glass capillaries under inert gas. Spectra are plotted with: Spectragryph, software for optical spectroscopy, V1.2.15 by Dr. F. Menges.

Single crystals for XRD analyses were mounted in inert oil under protective atmosphere. Data for X-ray crystal structure determination were obtained with a STOE STADIVARI, a STOE IPDS II diffractometer, and a Bruker APEX-II CCD using graphite-monochromated Mo−Kα radiation (λ=0.71073 Å). All structures were refined to convergence against F^2 using programs from the SHELX family.

Syntheses:

Synthesis of $CH_3@1$ [$CH_3@Na_{13}(OtBu)_{12}$]:

In *n*-hexane: Freshly prepared Trimethylsilylmethylsodium, NaR, (0.110 g, 1.0 mmol) and freshly sublimated sodium tert-butoxide, NaO*t*Bu, (1.250g, 13.0 mmol), were suspended in *n*-hexane (4 mL). The temperature was carefully increased to 60°C until the solution became clear. After keeping the solution at 60°C for several minutes, it was allowed to cool slowly. Storing the solution for more 24 h at room temperature afforded large yellowish octahedral crystals of compound **$CH_3@1$** (yield: 0,148 g, 39 %). [$CH_3@Na_{13}(OtBu)_{12}$], **$CH_3@1$**: ^{1}H NMR (400 MHz, 294 K, [D12]cyclohexane, TMS): δ = 1.26 (s, 9H; O*t*Bu), -3.36 ppm (s, 3H, CH_3); ^{1}H NMR (400 MHz, 294 K, [D6]benzene, TMS): δ = 1.38 (s, 9H; O*t*Bu), -3.23 ppm (s, 3H, CH_3); ^{13}C NMR (101 MHz, 298 K, [D6]benzene, TMS): δ = 66.6 (O*C*Me$_3$), 37.6 (OC*Me$_3$*), -21.6 ppm (*C*H$_3$).

In *n*-heptane: Freshly prepared Trimethylsilylmethylsodium, NaR, (0.220 g, 2.0 mmol) and freshly sublimated sodium tert-butoxide, NaO*t*Bu, (1.250g, 13.0 mmol), were suspended in *n*-heptane (4 mL). The temperature was carefully increased to 100°C until the solution became clear. After keeping the solution at 100°C for 10 h. To increase the yield, the solution was allowed to cool slowly. Storing the solution for more 24 h at room temperature afforded large yellowish octahedral crystals of compound **$CH_3@1$** (yield: 0,70 g, 60 %).

Synthesis of CH_2D@1 [CH_2D@Na_{13}(O*t*Bu)$_{12}$]:

Freshly prepared partially α-deuterated trimethylsilylmethyllithium, $LiCHDSiMe_3$, (0.055 g, 0.5 mmol) and freshly sublimated sodium *tert*-butoxide, NaO*t*Bu, (0.625g, 6.5 mmol), were suspended in *n*-heptane (2 mL) by carefully increasing the temperature to 100°C until the solution became clear. The temperature of the solution was kept at 100°C for 10h, then the solution was allowed to cool slowly. Storing the solution for more 24 h at room temperature afforded large yellowish octahedral crystals of **CH_2D@1** (yield: 0,268 g, 46 %). [CH_2D@Na_{13}(O*t*Bu)$_{12}$], **CH_2D@1**: ^{1}H NMR (400 MHz, 294 K, [D12]cyclohexane, TMS): δ = 1.26 (s, 9H; O*t*Bu), -3.36 (s, 3H, CH_3). : 1H NMR (400 MHz, 294 K, [D6]benzene, TMS): δ = 1.38 (s, 9H; O*t*Bu), -3.23 (s, 3H, CH_3).

Synthesis of $LiCHDSiMe_3$:

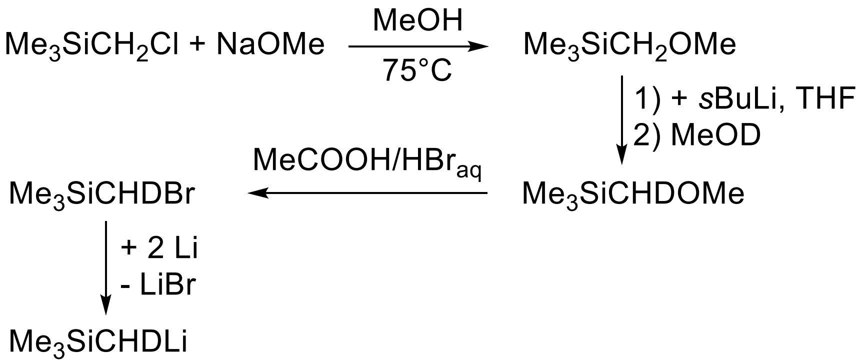

<u>Synthesis of methyl trimethylsilylmethyl ether [Me_3SiCH_2OMe]:</u> Trimethylsilylmethyl chloride [Me_3SiCH_2Cl] (40 ml, 35.2 g, 287 mmol) was added to a solution of sodium methoxide in methanol (25 wt-%, 90 ml, 85 g, 393 mmol). The mixture was stirred at 75°C for 3 days. Methanol was removed by fractionated distillation. The product was distilled at temperatures between 75-85°C, washed with water, and dried over Na_2SO_4. Yield: 24.9 g, 32.8 ml, 155,5 mmol, 54.2 %.

<u>Synthesis of α-mono-deuterated methyl trimethylsilylmethyl ether [$Me_3SiCHDOMe$]:</u>[2] A solution of $Me_3SiCHDOMe$ (9.2 ml, 7.0 g, 43.7 mmol) in THF (60 ml) was cooled to -80°C. A solution of *s*BuLi in cyclohexane (1.4 M, 46 ml, 64.4 mmol, 1.47 equivalents) was added slowly over a period of 40 min. Then the solution was slowly allowed to warm to -25°C, were it was kept for further 45 min. After addition of methylcyclohexane (20 ml) volatile components were removed in vacuum in a temperature range -30°C to -10°C until 50 ml mixture were left. After addition of deuterated methanol [MeOD] (4.0 ml) at -20°C the mixture was warmed to room temperature and diluted hydrochloric acid (10 %) was added. The organic and aqueous phases were separated. ^{1}H NMR (400 MHz, 294 K, [D]chloroform, TMS): δ = 3.34 (Me), 3.08 (CHD), 0.04 ppm ($SiMe_3$).

<u>Synthesis of α-mono-deuterated bromotrimethylsilylmethane [$Me_3SiCHDBr$]:</u> The solution of α-mono-deuterated methyl trimethylsilylmethyl ether in methylcyclohexane was heated with glacial acetic acid (25 ml) and hydrobromic acid (25 ml) at 80°C for 48h. The mixture was washed with water eight times and subsequently dried with Na_2SO_4. ^{1}H NMR (400 MHz, 294 K, [D]chloroform, TMS): δ = 2.46 (CHD), 0.14 ppm ($SiMe_3$). ^{13}C NMR (101 MHz, 294 K, [D]chloroform, TMS): δ = 18.2 (CHD), -2.5 ppm ($SiMe_3$).

Synthesis of α-mono-deuterated trimethylsilylmethyllithium [$LiCHDSiMe_3$]: A solution of α-mono-deuterated bromotrimethylsilylmethyl in methylcyclohexane (8 ml) was mixed with diethylether (20 ml) and 0.2 g lithium was added. After the refluxing stopped, the mixture was heated for 30 min. The mixture was filtered and the volatile compounds removed in vacuum. $LiCHDSiMe_3$ was obtained by sublimation (120°C at 10^{-2} mbar). Yield: ~ 0.7 g.

Synthesis of $LiCHDSi(CH_2D)_3$:

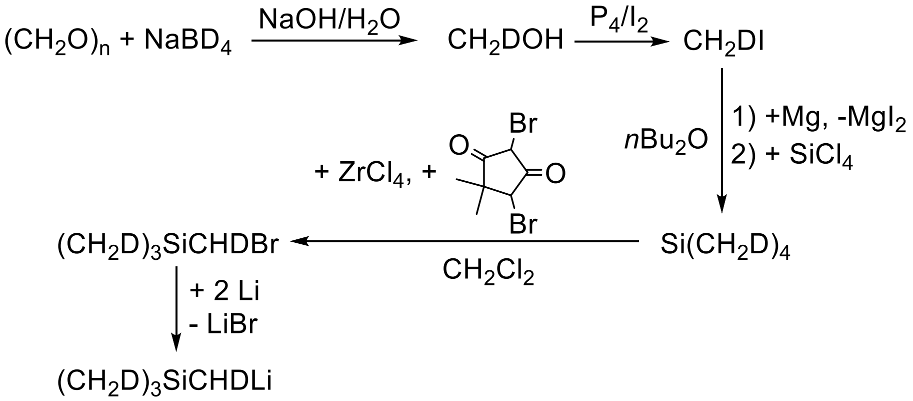

Synthesis of mono-deuterated methanol [CH_2DOH]: Sodium borodeuterid [$NaBD_4$] (5.0 g, 97 mmol) was added to a solution of sodium hydroxide [NaOH] (0.8 g, 20 mmol) in water (200 ml) at 0°C. Solid paraformaldehyde [$(CH_2O)_n$] (2.87 g, 96 mmol, 4 eq) was added slowly under stirring over ½ h while keeping the temperature at 0°C. Then the solution was allowed to warm to room temperature and stirred for 3d. The solution was acidified with concentrated hydrochloric acid (11 ml), the product was distilled off and dried with Na_2SO_4 and distilled again. Yield: 3.6 ml, 4.1 ml, 109 mmol, 28 %.

Synthesis of mono-deuterated iodomethane [CH_2DI]:[3] A mixture of red phosphorus (3.0 g, 97 mmol) in water (20 ml) was cooled to 0°C and solid iodine [I_2] (20.0 g, 78.8 mmol) was added slowly keeping this temperature. After warming to room temperature mono-deuterated methanol [CH_2DOH] (3.6 g, 109 mmol) was added slowly. This mixture was kept at reflux (oil bath: 65°C) for 2h, then the product was distilled off. Yield: 5.37 g, 2.3 ml, 37.6 mmol, 34.5 %)

Synthesis of tetra-deuterated tetramethylsilane [$Si(CH_2D)_4$]: To a mixture of magnesium (13 g, 535 mmol) in di-*n*-butyl ether [*n*Bu_2O] (80 ml) was slowly added a mixture of mono-deuterated iodomethane [CH_2DI] (5.73 g, 125 mmol) in di-*n*-butyl ether (20 ml) over ½ h, the temperature was kept below 30°C. The mixture was stirred for another 1 h, then a mixture of tetrachlorosilane [$SiCl_4$] (1.1 ml, 1.6 g, 9.5 mmol) in in di-*n*-butyl ether (10 ml) was added at room temperature over ½ h. After stirring for 1h, the product was distilled off (oil bath temperature 120°C) and collected at -78°C and dried with concentrated sulfuric acid.

Synthesis of tetra-deuterated trimethylsilylmethylbromide [$(CH_2D)_3SiCHDBr$]:[4] Tetra-deuterated tetramethylsilane (3.2 g, 35 mmol) and 1,3-dibromo-5,5-dimethylhydantoin (10.0 g, 35 mmol) were added to a suspension of zirconium(IV) chloride (1.0 g, 11 mmol) in dichloromethane (40 ml) at room temperature. The mixture was stirred under white light irradiation for 4d. All solvents were removed under vacuum and separated in a fractionated distillation.

Synthesis of $LiCD_2Si(CD_3)_3$:

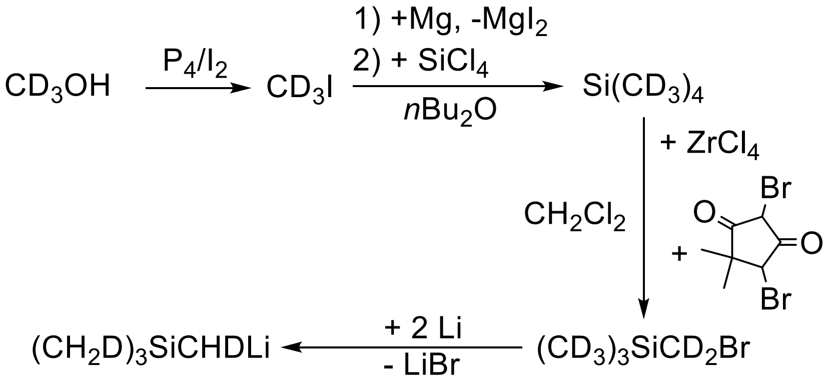

Synthesis of tri-deuterated iodomethane [CD_2I]:[3] A mixture of red phosphorus (6.0 g, 194 mmol) in water (40 ml) was cooled to 0°C and solid iodine [I_2] (40.0 g, 158 mmol) was added slowly keeping this temperature. After warming to room temperature mono-deuterated methanol [CD_3OH] (10 ml, 8.9 g, 247 mmol) was added slowly. This mixture was kept at reflux (oil bath: 65°C) for 2h, then the product was distilled off. Yield: 18.6 g, 8.0 ml, 162 mmol, 65.6 %)

Synthesis of tetra-deuterated tetramethylsilane [$Si(CD_3)_4$]: To a mixture of magnesium (40 g, 1.6 mol) in di-*n*-butyl ether [*n*Bu_2O] (400 ml) was slowly added a mixture of mono-deuterated iodomethane [CD_3I] (115 g, 793 mmol) in di-*n*-butyl ether (100 ml) over 3 h, the temperature was kept below 30°C. The mixture was stirred for another 1 h, then a mixture of tetrachlorosilane [$SiCl_4$] (33.1 g, 195 mmol) in in di-*n*-butyl ether (50 ml) was added at room temperature over 2 h. After stirring for 1h, the product was distilled off (oil bath temperature 120°C) and collected at -78°C and dried with concentrated sulfuric acid. Yield: 11.3 g, 113 mmol, 57 %.

Synthesis of deuterated tetramethylsilane [$Si(CD_3)_4$]: To a mixture of magnesium (10 g, 411 mmol) in di-*n*-butyl ether [*n*Bu_2O] (75 ml) was slowly added a mixture of deuterated iodomethane [CD_3I] (7.5 ml, 18.1 g, 125 mmol) in di-*n*-butyl ether (20 ml) over ½ h, the temperature was kept below 30°C. The mixture was stirred for another 1 h, then a mixture of tetrachlorosilane [$SiCl_4$] (3.6 ml, 5.32 g, 31.3 mmol) in in di-*n*-butyl ether (15 ml) was added at room temperature over ½ h. After stirring for 1h, the product was distilled off (oil bath temperature 120°C) and collected at -78°C and dried with concentrated sulfuric acid. Yield: 3.0 g, 30.0 mmol, 96 %).

Synthesis of fully deuterated trimethylsilylmethylbromide [$(CD_3)_3SiCD_2Br$]:[4] Deuterated tetramethylsilane (11.3 g, 113 mmol) and 1,3-dibromo-5,5-dimethylhydantoin (32.3 g, 113 mmol) were added to a suspension of zirconium(IV) chloride (2.6 g, 11 mmol) in dichloromethane (120 ml) at room temperature. The mixture was stirred under white light irradiation for 4d. All solvents were removed under vacuum and separated in a fractionated distillation.

Synthesis of CD_3@1 [CD_3@Na_{13}(O*t*Bu)$_{12}$]:

Sodium *tert*-butoxide, NaO*t*Bu (0.38 g, 4.0 mmol), and fully deuterated trimethylsilylmethyl-lithium, $LiCD_2Si(CD_3)_3$ (0.053 g, 0.5 mmol), were suspended in *n*-heptane and stirred an heated to 100°C until the solution became clear. The temperature of the solution was kept at 100°C for 10 h, then the solution was allowed to cool slowly. Storing the solution for more 24 h at room temperature afforded large yellowish octahedral crystals of **CD_3@1** (0.144 g, 0.12 mmol, 36 % based on NaO*t*Bu). **CD_3@Na_{13}(O*t*Bu)$_{12}$**, **CD_3@1**: ^{1}H NMR (400 MHz, 294 K, [D12]cyclohexane, TMS): δ = 1.38 (s, 9H; O*t*Bu).

Synthesis of NaOMeCyc [NaO(c-C_6H_{10}Me]: Sodium 1-methylcyclohexanolate (NaOMeCyc), was obtained through the reaction of 1-methylcyclohexanol (10.0 g, 87.6 mmol) and an excess of sodium metal (3.0 g, 131.4 mmol) dispersed in dry *n*-heptane (250 mL). The mixture was allowed to reflux for 7d until the complete dissolution of the initially forming crystals. The clear solution was allowed to cool to room temperature, and the remaining sodium metal was removed manually. The solvent was removed by distillation. By sublimation of the crystalline residue at 240°C ($1x10^{-3}$ mbar) NaOMeCyc was yielded as colorless crystalline solid. (10.8 g, 79.3 mmol, 91%). ^{1}H-NMR (400 MHz, 294 K, [D6]benzene, TMS), δ = 1,68 - 1,36 (m, 10H, C_6H_{10}), 1,16 ppm (s, 3H, *Me*). ^{13}C NMR (101 MHz, 298 K, [D6]benzene, TMS): δ = 66.81 (O*C*C_5H_{10}Me), 47.05 (s, 2C, β-CH_2), 33.78 (Me), 27.20 (δ-CH_2), 25.39 ppm (γ-CH_2).

Synthesis of CH_3@Na_{13}(OMeCyc)$_{12}$]: Trimethylsilylmethylsodium, NaR, (0,113 g, 0.5 mmol) and freshly sublimated 1-methylcyclohexanolate (NaOMeCyc, (0.883 g, 6.5 mmol), were suspended in *n*-heptane (4 mL). The temperature was carefully increased to 100°C until the solution became clear and was kept at 100°C for 10h. The solution was allowed to cool slowly to room temperature. Storing for more 24 h afforded large yellowish cubic crystals of CH_3@Na_{13}(OMeCyc)$_{12}$ (yield: 0,292 g, 35 %). **[CH_3@Na_{13}(OMeCyc)$_{12}$]**: ^{1}H NMR (400 MHz, 294 K, [D12]cyclohexane, TMS): δ = 1.65-1.15 (m, 13H; *Me*$C_6$$H_{10}$), -3.21 (s, 3H, CH_3). **CH_3@Na_{13}(OMeCyc)$_{12}$]**: ^{1}H NMR (400 MHz, 294 K, [D6]benzene, TMS): δ = 1.78 – 1.21 (m, 13H; *Me*$C_6$$H_{10}$), -2.99 (s, 3H, CH_3).

General syntheses of X@1 [X@Na_{13}(O*t*Bu)$_{12}$] (X = CN, ^{13}CN, Cl, Br, I, OCN, SCN, N_3, 1-$^{15}N_3$, NO_3, $^{15}NO_3$): NaO*t*Bu was suspended in *n*-heptane and commercial trimethylsilyl compound (Me_3Si-X), was added slowly, according the table below. Only the addition of trimethylsilylcyanide produced a clear solution. The temperature of the suspension was increased to 100°C, stirring was continued until the solution became clear. This solution was kept at 100°C for 10 h, (some solutions already yielded crystals). After cooling slowly and storing for more than 24 h at room temperature afforded large colourless octahedral crystals.

Table S1: General syntheses of compounds **X@1**.

Compound	NaO*t*Bu	Me_3SiX	*n*-heptane	Yield
**CN@1**	1.25 g, 13.0 mmol	0.14 ml, 1.0 mmol	10 mL	0.81 g, 0.670 mmol, 67 %
**^{13}CN@1**	0.65 g, 6.5 mmol	0.07 ml, 0.5 mmol	5 mL	0.338 g, 0.281 mmol, 56 %
**Cl@1**	1.25 g, 13.0 mmol	0.13 ml, 1.0 mmol	10 mL	0.871 g, 0.720 mmol, 72 %
**Br@1**	1.25 g, 13.0 mmol	0.13 ml, 1.0 mmol	10 mL	0.724 g, 0.577 mmol, 58 %
**I@1**	1.26 g, 13.1 mmol	0.14 ml, 1.0 mmol	10 mL	0.730 g, 0.561 mmol, 56 %
**OCN@1**	1.25 g, 13.0 mmol	0.14 ml, 1.0 mmol	10 mL	0.842 g, 0.692 mmol, 69 %
**SCN@1**	1.25 g, 13.0 mmol	0.14 ml, 1.0 mmol	10 mL	0.580 g, 0.470 mmol, 47 %
**N_3@1**	1.26 g, 13.1 mmol	0.15 ml, 1.0 mmol	10 mL	0.862 g, 0.708 mmol, 71 %
**1-$^{15}N_3$@1**	0.65 g, 6.5 mmol	0.7 ml, 0.5 mmol	5 mL	0.429 g, 0.352 mmol, 70 %
**NO_3@1**	1.25 g, 13.0 mmol	0.12 ml, 0.9 mmol	10 mL	0.307 g, 0.248 mmol, 28 %
**$^{15}NO_3$@1**	0.39 g, mmol	0.04 ml, 0.3 mmol	7 mL	0.04 g, 0.03 mmol, 10 %

Synthesis of Na_{11}(O*t*Bu)$_{12}$F, 2-F: Sodium *tert*-butoxide, NaO*t*Bu, (1.240 g, 12.9 mmol) was suspended in *n*-heptane (10ml) and Fluorotriphenylsilane (0.272 g, 0.98 mmol) was added. The suspension was heated to 100°C under stirring until the solution became clear. The temperature of the solution was kept at this temperature for seven days, then the solution was allowed to cool slowly. Storing the solution for more 24 h at ambient temperature afforded colourless crystals of **Na_{11}(O*t*Bu)$_{10}$F** (0.587 g, 0.586 mmol, 60 % based on Fluorotriphenylsilane). ^{1}H NMR (400 MHz, 294 K, [D6]benzene, TMS): δ = 1.33 ppm (s, 9H; O*t*Bu); ^{19}F NMR (376 MHz, 298 K, [D6]benzene, $CFCl_3$): δ =223.29 ppm.

Table S2: Crystallographic data and refinement for compounds **X@1** and **2-F** (X = CH_3, CN, I, SCN, NO_3).

Compound	CH_3@1	CN@1	2-F	I@1	SCN@1	NO_3@1
Formula	$C_{49}H_{111}O_{12}$	$C_{49}H_{108}NO_{12}$	$C_{40}H_{90}FO_{10}$	$C_{48}H_{108}IO_{12}$	$C_{49}H_{108}NO_{12}S$	$C_{48}H_{108}NO_{15}$
M_r [g mol^{-1}]	1190.65	1201.65	1002.54	1302.56	1233.63	1237.64
Crystal size [mm^3]	0.4x0.4x0.4	0.42x0.29x0.15	0.88x0.68x0.44	1.76x1.25.0.75	0.56x0.42x0.35	0.64x0.47x0.14
Crystal system	cubic	cubic	orthorhombic	cubic	cubic	cubic
Space group	$Pa\overline{3}$	$Pa\overline{3}$	*Pbca*	$Pa\overline{3}$	$Pa\overline{3}$	$Pa\overline{3}$
a [Å]	19.633(2)	19.3673(7)	22.685(5)	19.530(2)	19.397(2)	19.515(2)
b [Å]			19.877(4)			
c [Å]			27.057(5)			
α [°]	90	90	90	90	90	90
β [°]			90			
γ [°]			90			
V [Å^3]	7568(3)	7264.6(4)	12201(4)	7449(3)	7299(2)	7432(3)
Z	4	4	8	4	4	4
ρ_{calcd} [g cm^{-3}]	1.043	1.099	1.092	1.162	1.123	1.107
$\mu(Mo_{K\alpha})$ [mm^{-1}]	0.134	0.14	0.142	0.550	0.169	0.142
T [K]	193	120	173	173	120	173
$2\theta_{max}$ [°]	28.635	26.78	29.685	30.587	30.818	29.716
measured refl.	141181	19699	203886	20793	100593	53911
independent refl.	3133	2907	16638	3186	3757	3448
R(int)	0.1475	0.0875	0.0396	0.0478	0.0986	0.0887
refined parameters	415	235	736	258	377	283
*R*1 (*R*1 all data)	0.1295 (0.2035)	0.1102 (0.1585)	0.0402 (0.0566)	0.0602 (0.1441)	0.1307 (0.1760)	0.1276 (0.1733)
*wR*2 (*wR*2 all data)	0.4060 (0.4773)	0.3134 (0.3603)	0.1223 (0.1284)	0.1685 (0.2243)	0.3863 (0.4361)	0.3283 (0.3586)
GOOF	1.605	1.173	0.993	1.001	1.179	1.121
max, min peaks [eÅ^{-3}]	0.45, -0.41	0.463, -0.251	0.349, -0.354	0.93, -0.78	0.56, -0.45	0.65, -0.66
CCDC Number	2061407	2061408	2061409	2061410	2061411	2061412

Table S3: Crystallographic data for compounds **X@1** (X = CH_3, CN, Cl, Br, I, OCN, SCN, NO_3) at 173 K.

Compound	CH_3@1	CN@1	Cl@1	Br@1	I@1	OCN@1	SCN@1	N_3@1	NO_3@1
M_r [g mol^{-1}]	1191.27	1202.25	1211.68	1256.13	1303.14	1218.25	1233.63	1218.25	1238.24
Crystal size [mm^3]	0.6x0.5x0.2		1.4x1.1x0.7	0.8x0.6x0.4	1.8x1.2x0.7				0.6x0.5x0.3
a [Å]	19.4734(21)	19.430(3)	19.4387(12)	19.4715(15)	19.5296(10)	19.3796(12)	19.4555(12)	19.3517(16)	19.5148(5)
V [Å^3]	7384.5(14)	7334.9(21)	7345.2(8)	7382.4(10)	7448.7(6)	7278.3(8)	7364.2(8)	7246.9(10)	7431.8(3)
μ($Mo_{Kα}$) [mm^{-1}]	0.03	0.03	0.02	0.15	0.550	0.26	0.00	0.04	0.04
T [K]	173	173	173	173	173	173	173	173	173
2$θ_{max}$ [°]	22.11	19.04	24.13	20.38	30.59	20.23	21.87	19.03	28.46
measured refl.	2056	2404	8007	6054	20793	6596	7776	4637	24663

NMR spectroscopy

Table S4: NMR spectroscopic data for in [D6]benzene [ppm].

Compound	^{1}H		^{13}C			^{19}F	^{35}Cl	^{79}Br	^{127}I	^{15}N
	O*t*Bu	CH_3	O*t*Bu		central anion					
			*C*Me$_3$	C*Me*$_3$						
**CH_3@1**	1.38	3.23	66.6	37.6	-21.3					
**CN@1**	1.39	–								
**^{13}CN@1**	1.39	–	66.6	37.7	164.7					
**2-F**	1.33	–				-223.3				
**Cl@1**	1.39	–					-90.7			
**Br@1**	1.39	–						-141.0		
**I@1**	1.38	–							-124.7	
**OCN@1**	1.37	–								
**SCN@1**	1.35	–	66.6	37.2	132.9					
**N_3@1**	1.37	–								
**1-$^{15}N_3$@1**	1.37	–								
**NO_3@1**	1.36	–								
**$^{15}NO_3$@1**	1.37	–								-6.7

Figure S1-1: ^{1}H NMR of **CH_3@1**, in [D12]cyclohexane:

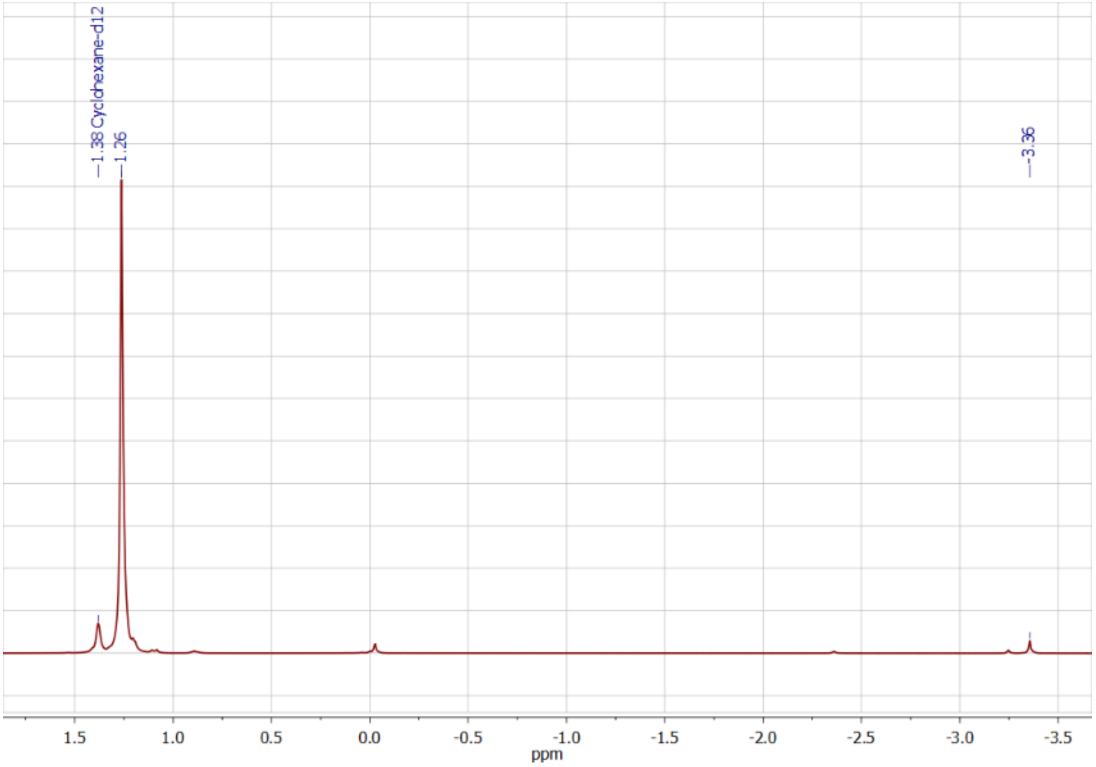

Figure S1-2: ^{1}H-^{13}C HSQC NMR of **CH_3@1**, in [D12]cyclohexane:

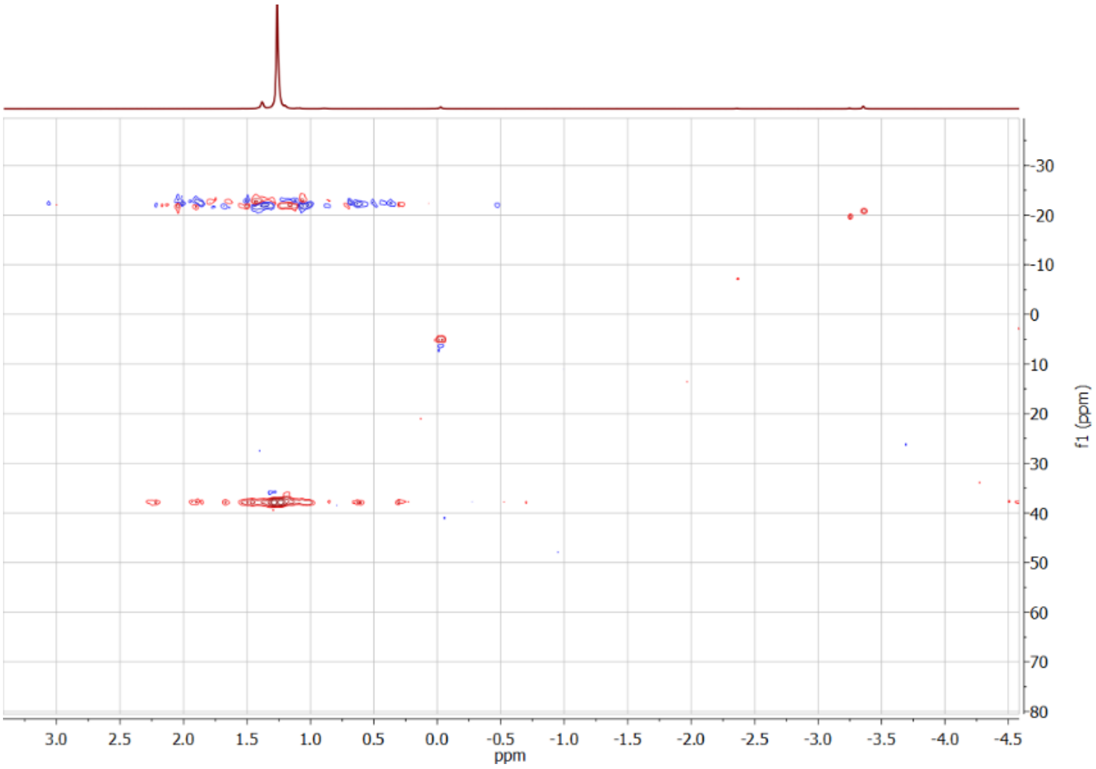

Figure S1-3: ^{1}H NMR of **CH_3@1**, in [D6]benzene:

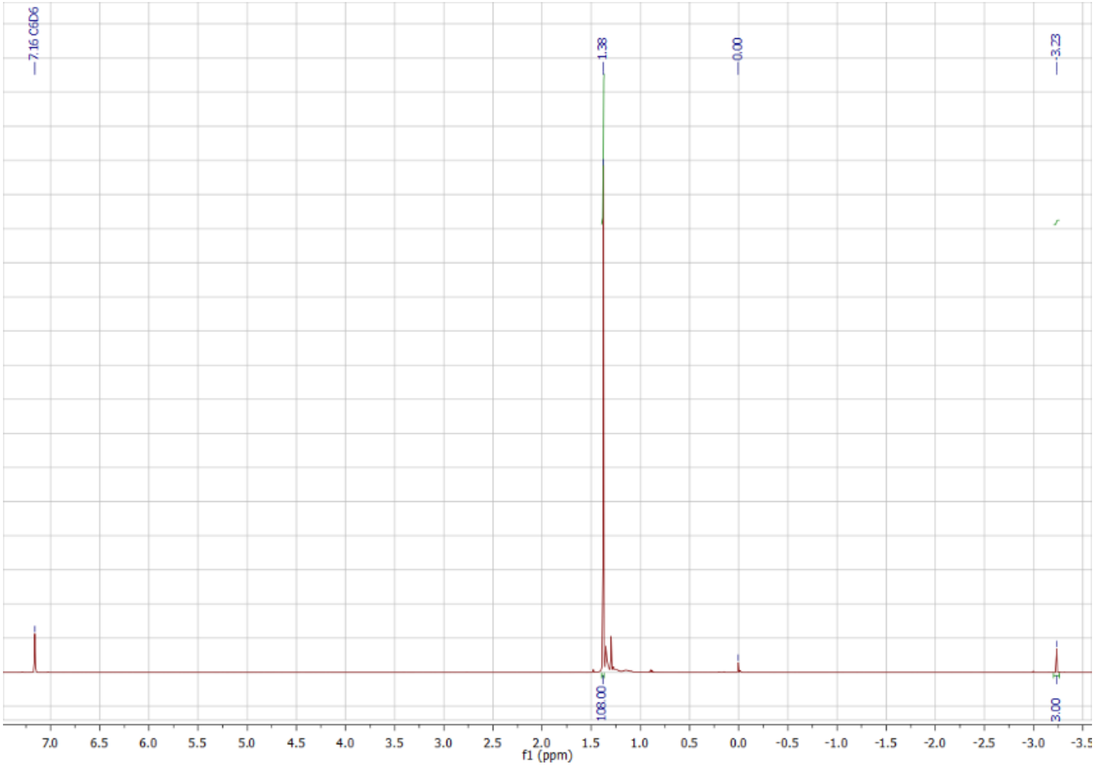

Figure S1-4: ^{13}C{^{1}H} NMR of **CH_3@1**, in [D6]benzene:

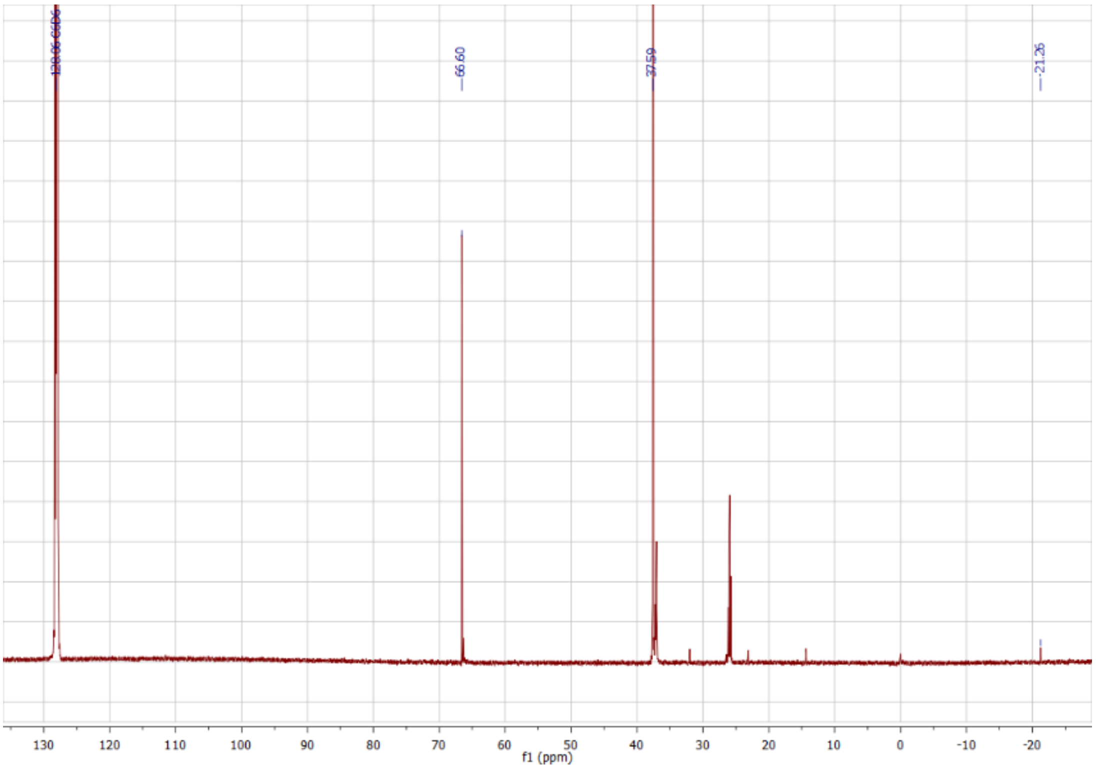

Figure S1-5: ^{13}C (^{1}H coupled) NMR of **CH_3@1**, in [D6]benzene:

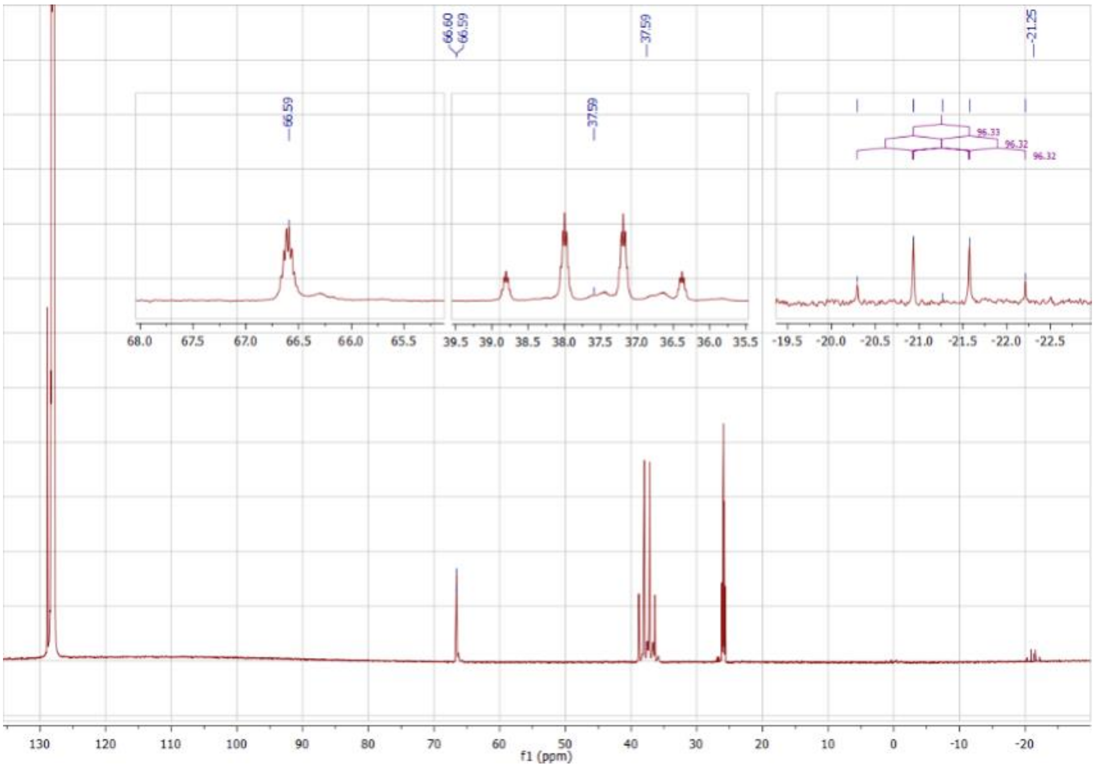

Figure S1-6: ^{1}H-^{13}C HSQC NMR of **CH_3@1**, in [D6]benzene:

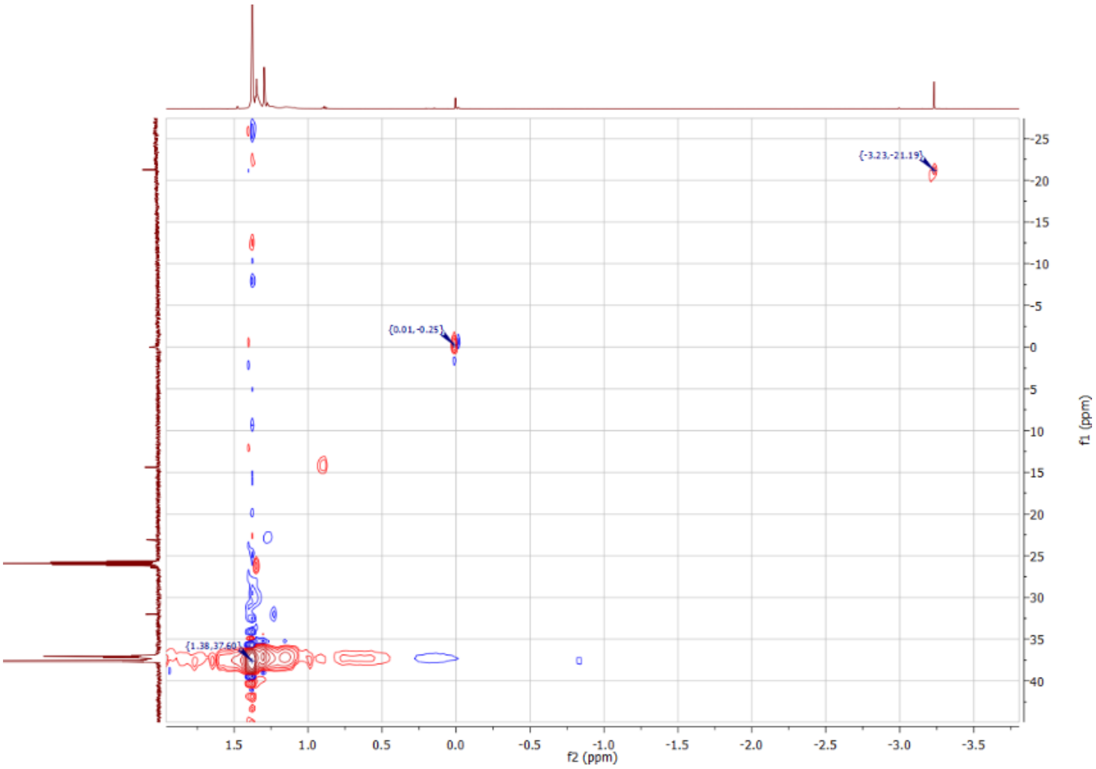

Figure S1-7: ^{1}H-^{13}C HSQC NMR of **CH_3@1**, in [D6]benzene with ^{1}H NMR and ^{1}H coupled ^{13}C NMR as traces (Figure 2):

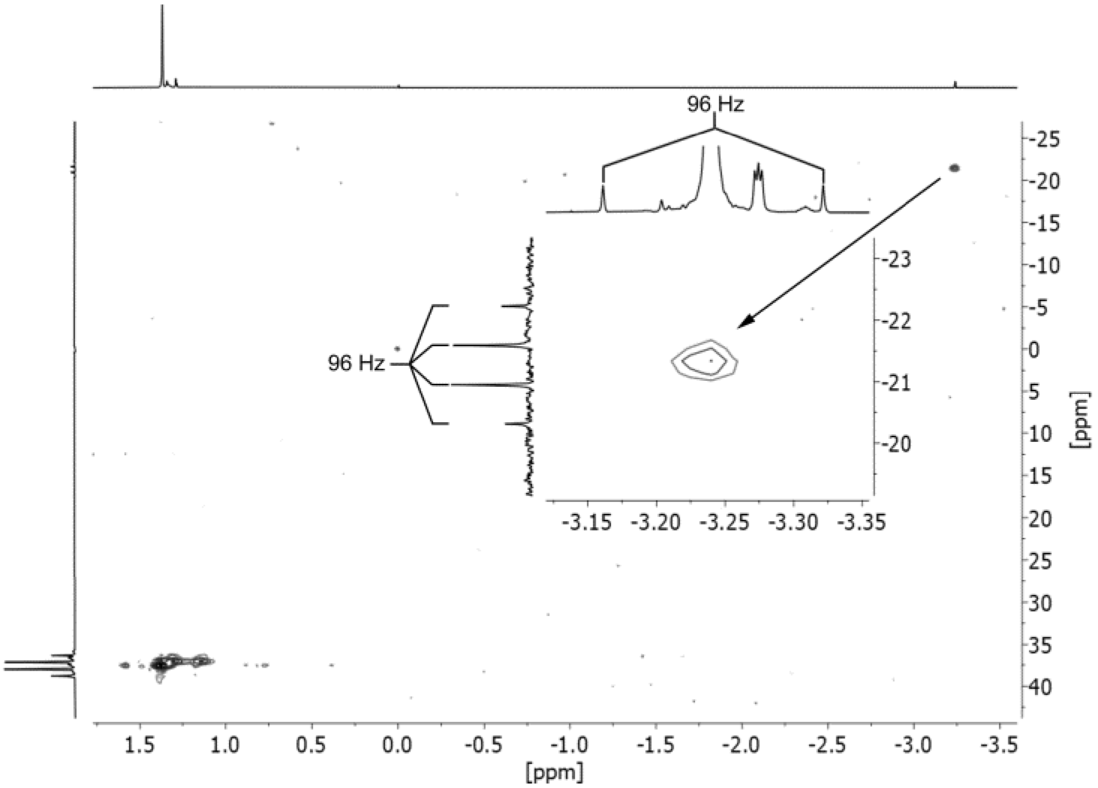

Figure S1-8: ^{1}H NMR of **CH_3@1**, in [D8]toluene:

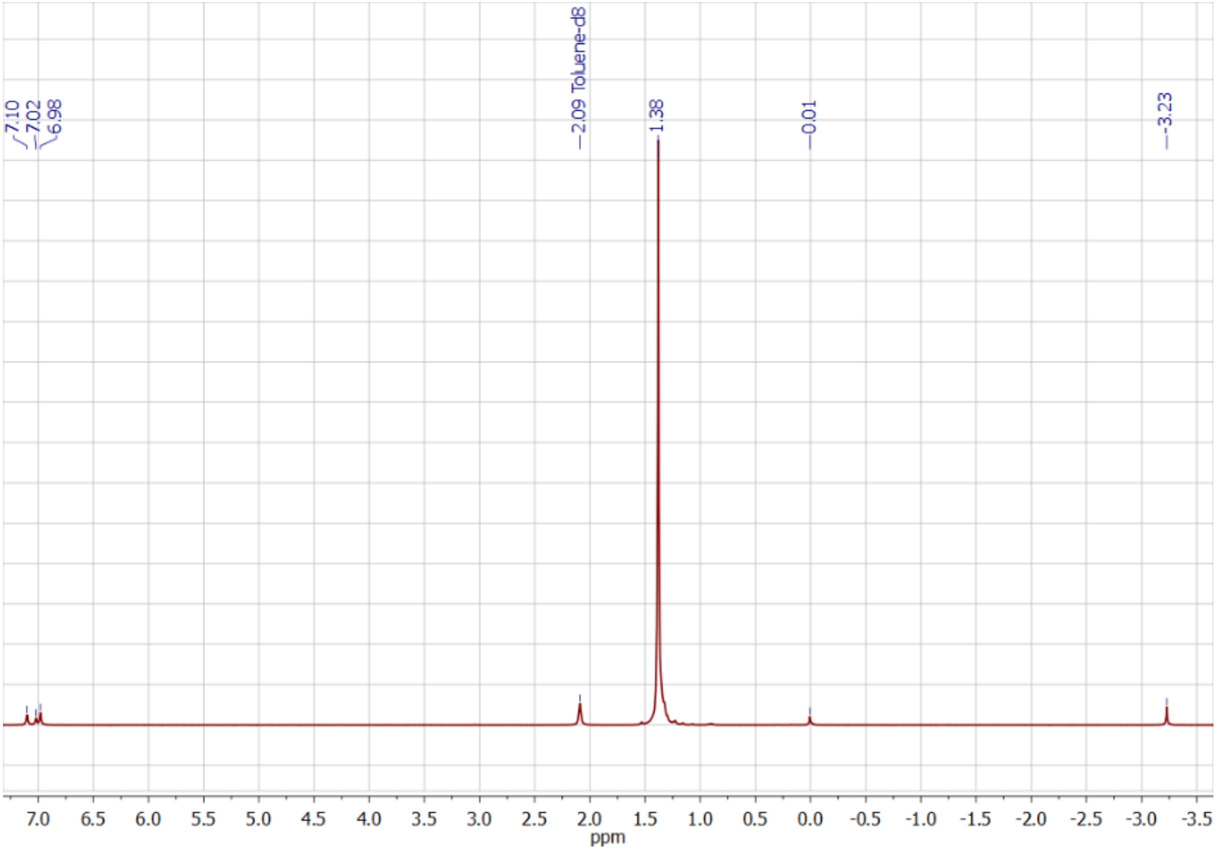

Figure S1-9: ^{1}H NMR of **CH_3@1**, in [D8]THF (with resonance of CH_3D at 0.17 ppm):

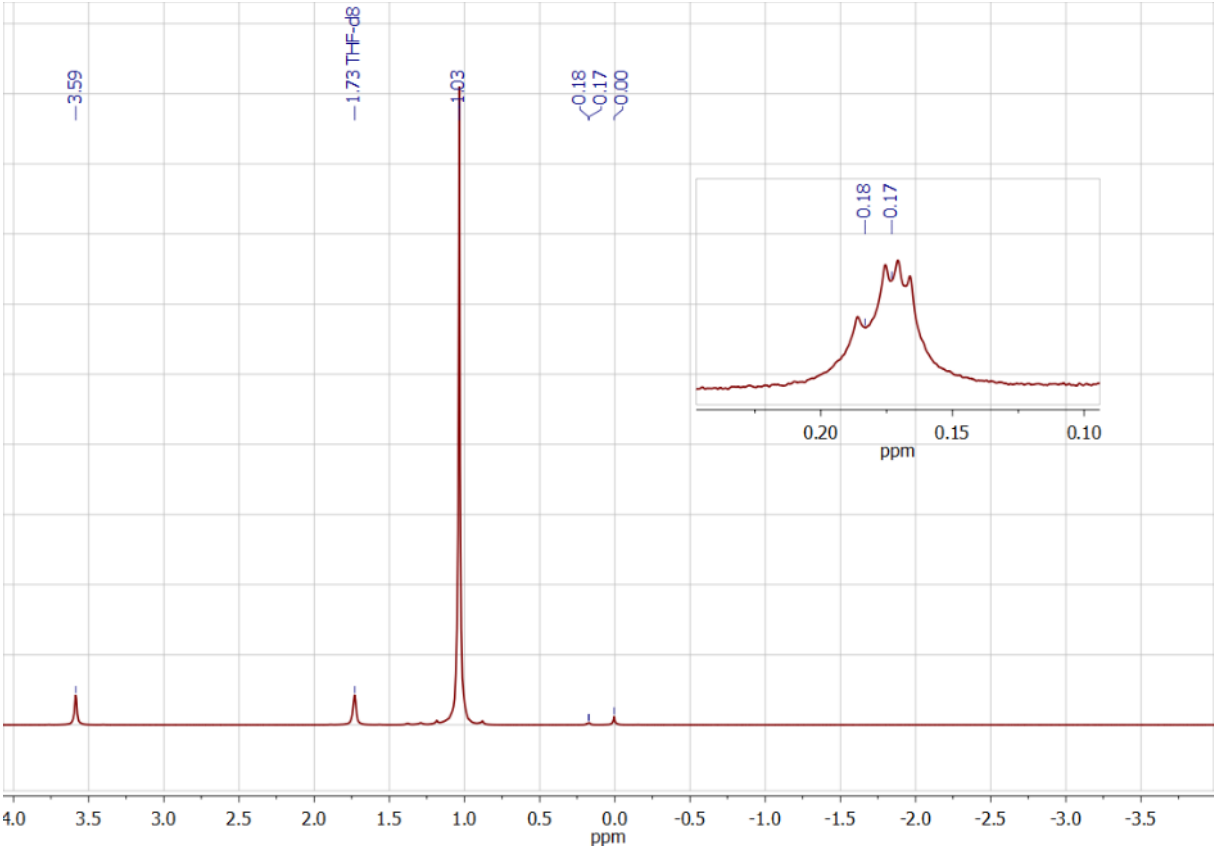

Figure S2-1: ^{1}H NMR of $LiCHDSiMe_3$ in [D6]benzene:

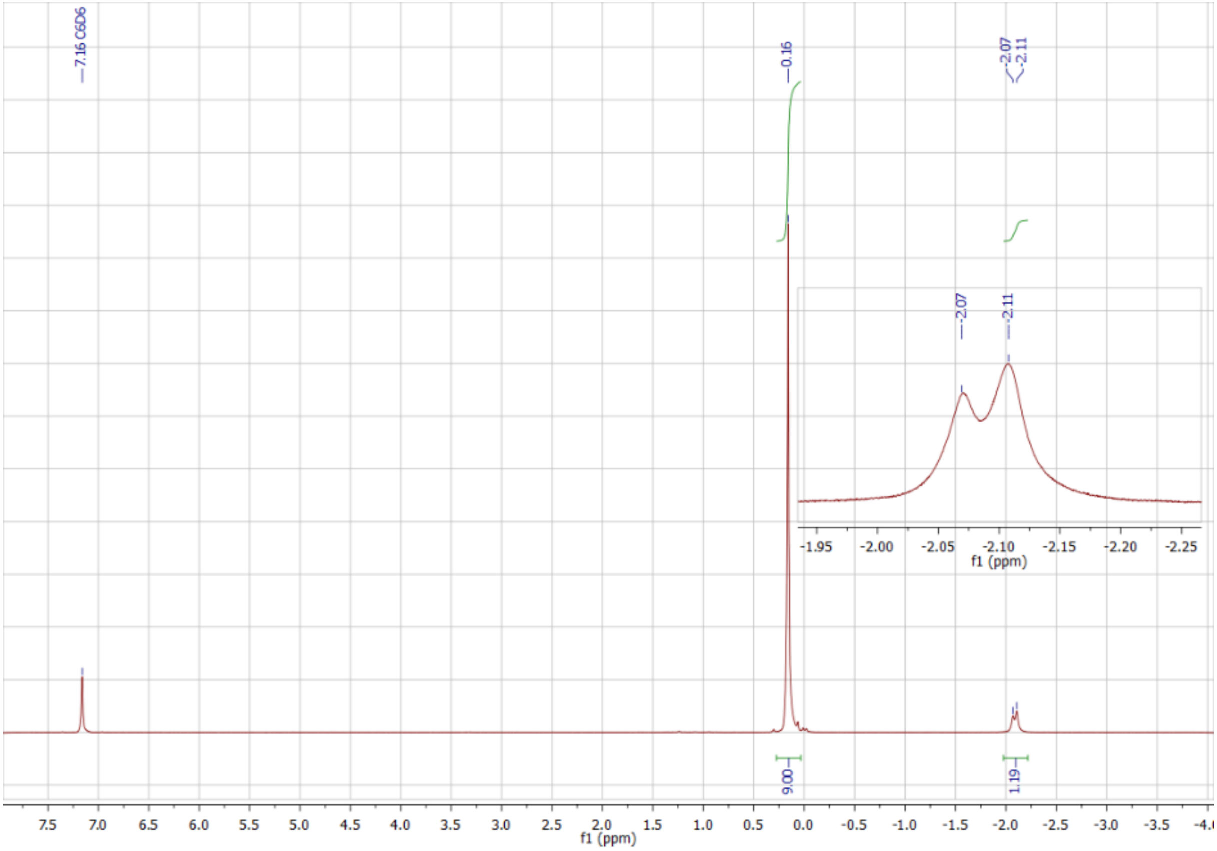

Figure S2-2: ^{1}H NMR of $LiCHDSiMe_3$ in [D6]benzene (detail):

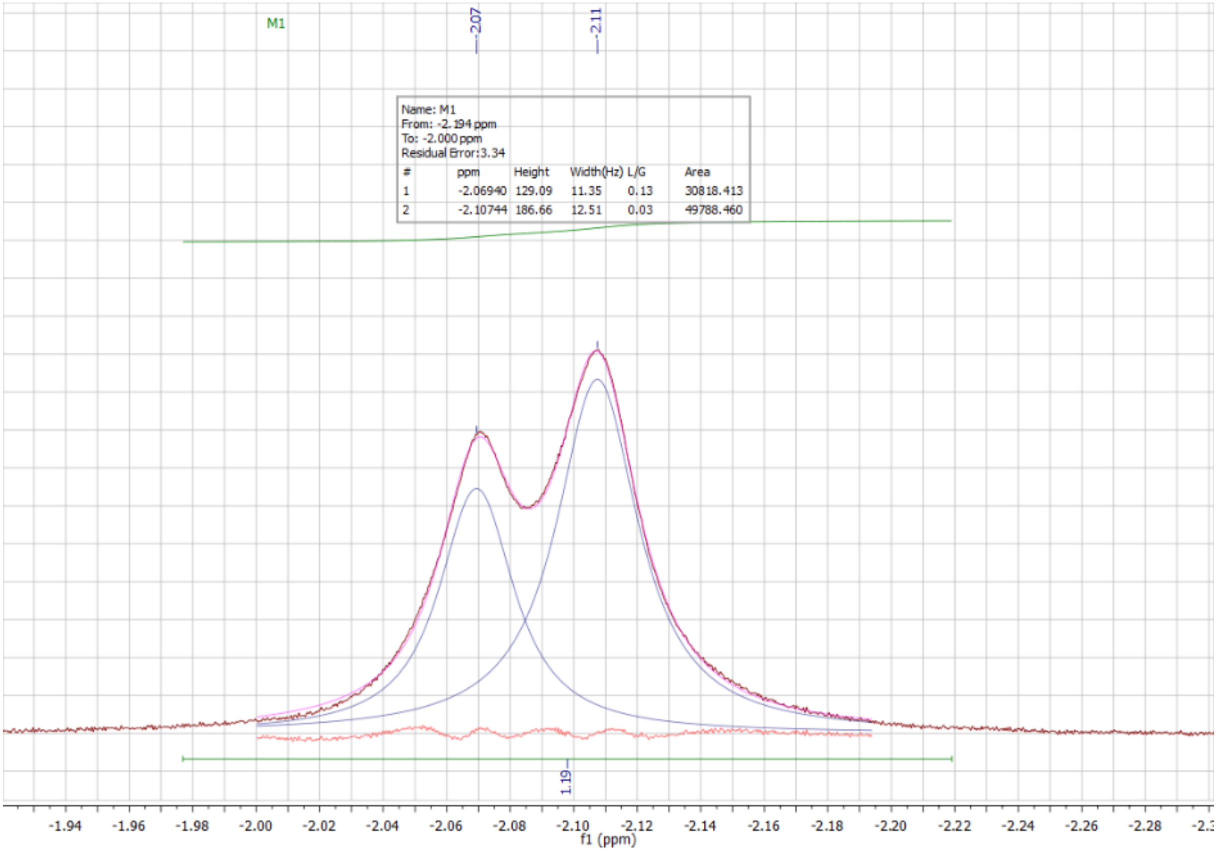

Figure S2-3: ^{7}Li NMR of LiCHDSiMe$_3$ in [D6]benzene:

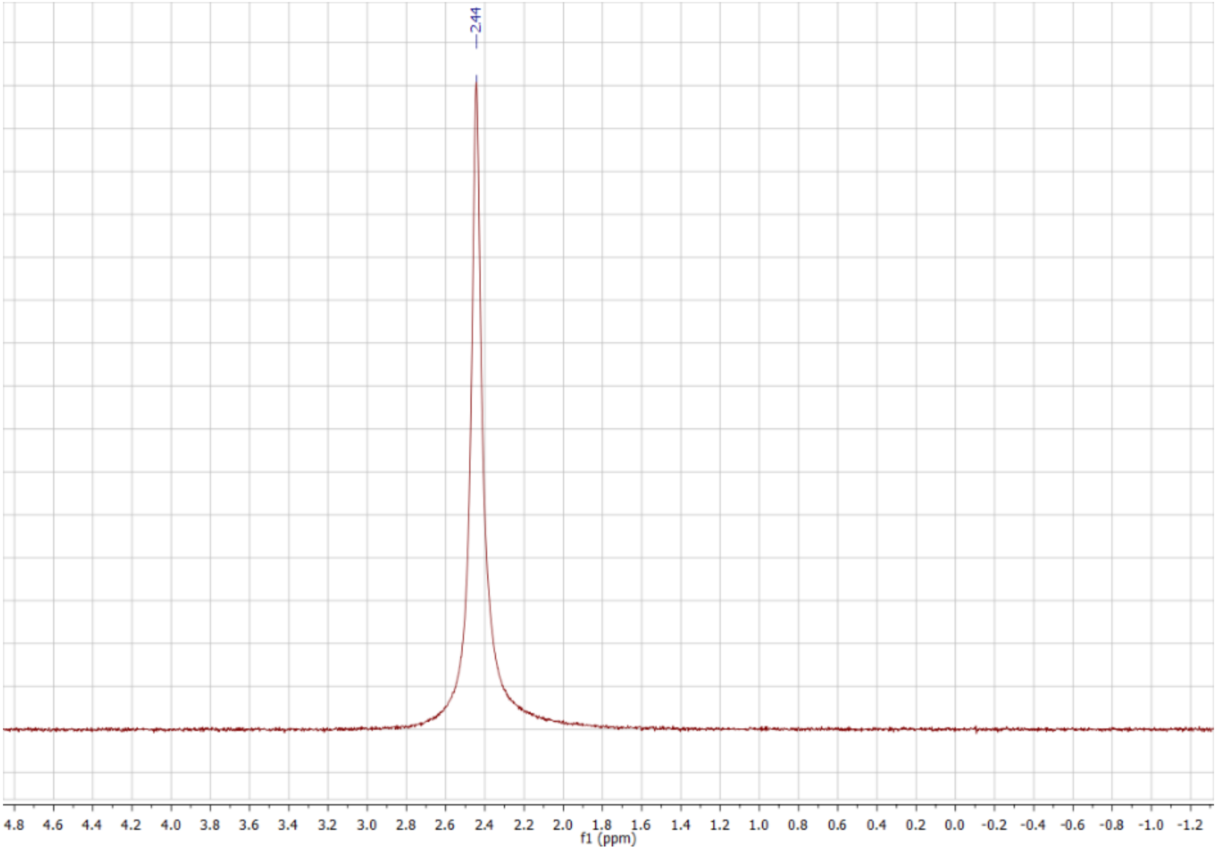

Figure S2-4: ^{1}H NMR of mixture of **CH$_3$@1** (3.23 ppm) and **CH$_2$D@1** (3.27 ppm) produced from LiCHDSiMe$_3$ in [D6]benzene (detail):

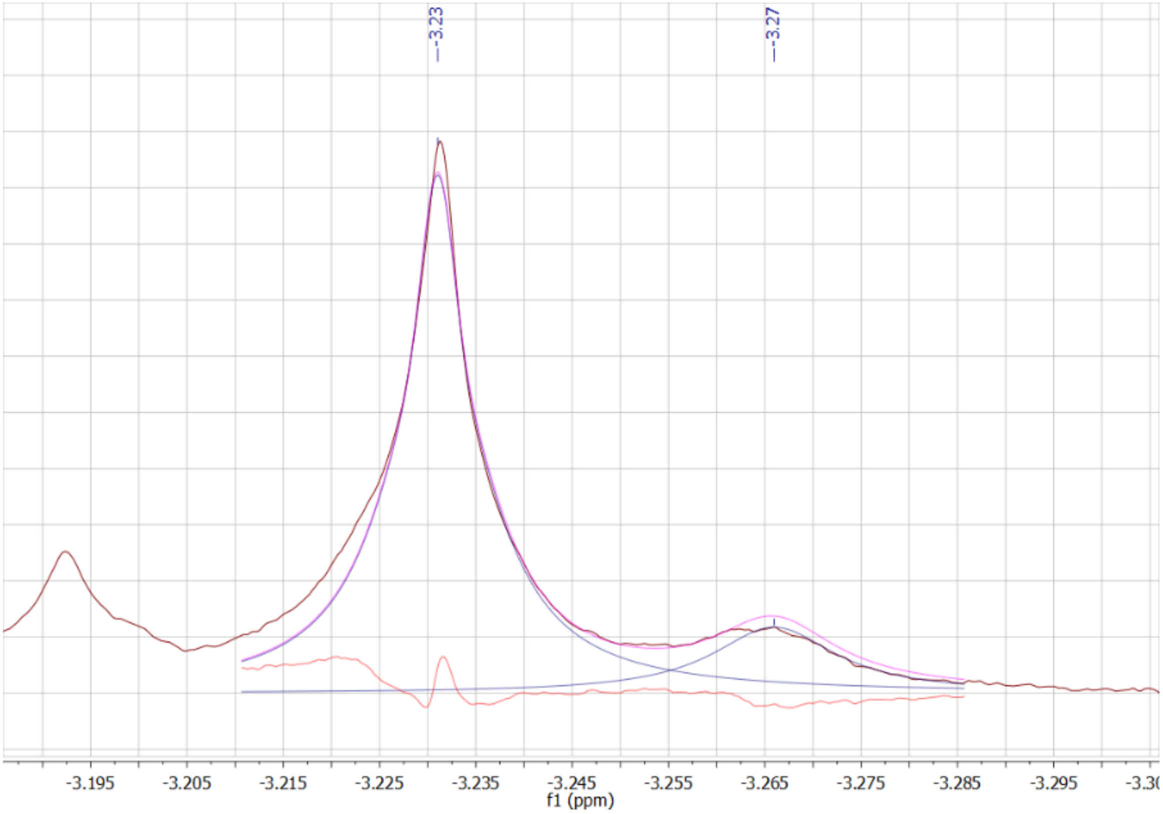

Figure S2-5: ^{1}H NMR of mixture of **CH_3@1** (3.36 ppm) and **CH_2D@1** (3.39 ppm) produced from $LiCHDSi(CH_2D)_3$ in [D12]cyclohexane (detail):

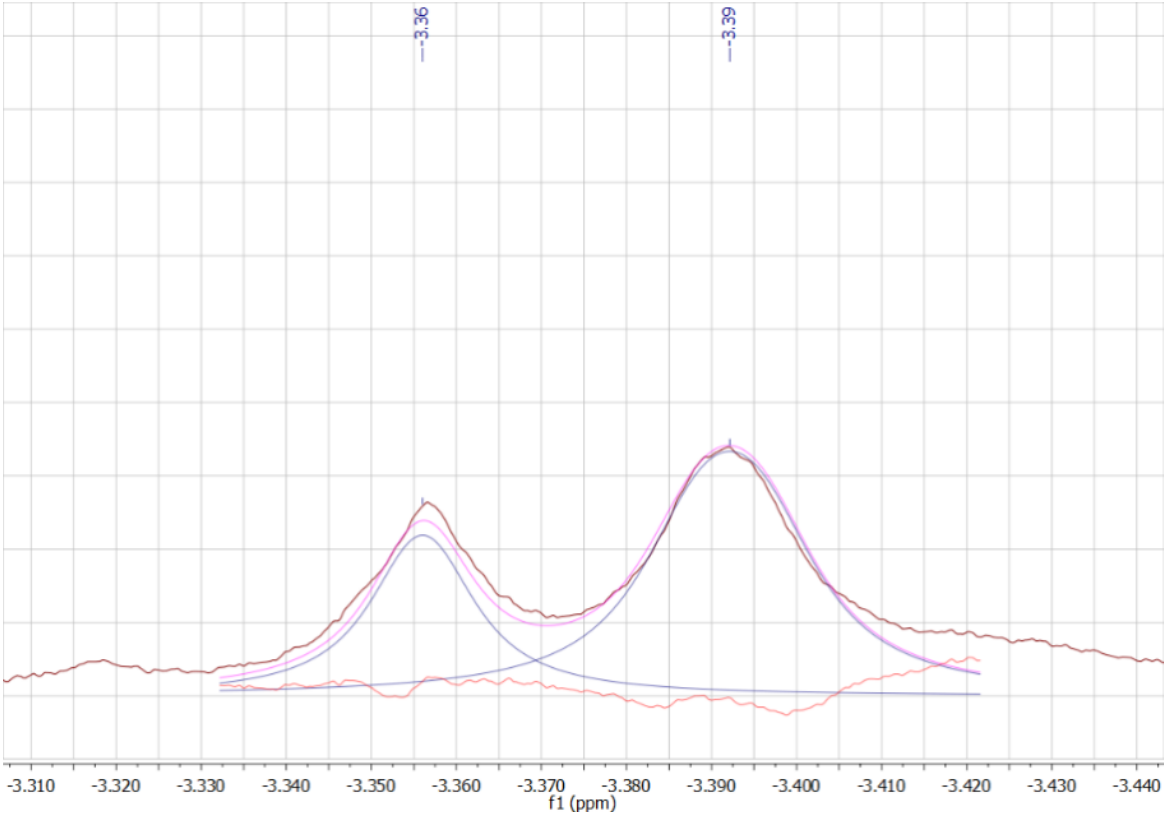

Figure S2-6: ^{2}H NMR **CH_2D@1** (3.36 ppm) produced from $LiCHDSi(CH_2D)_3$ in cyclohexane (detail):

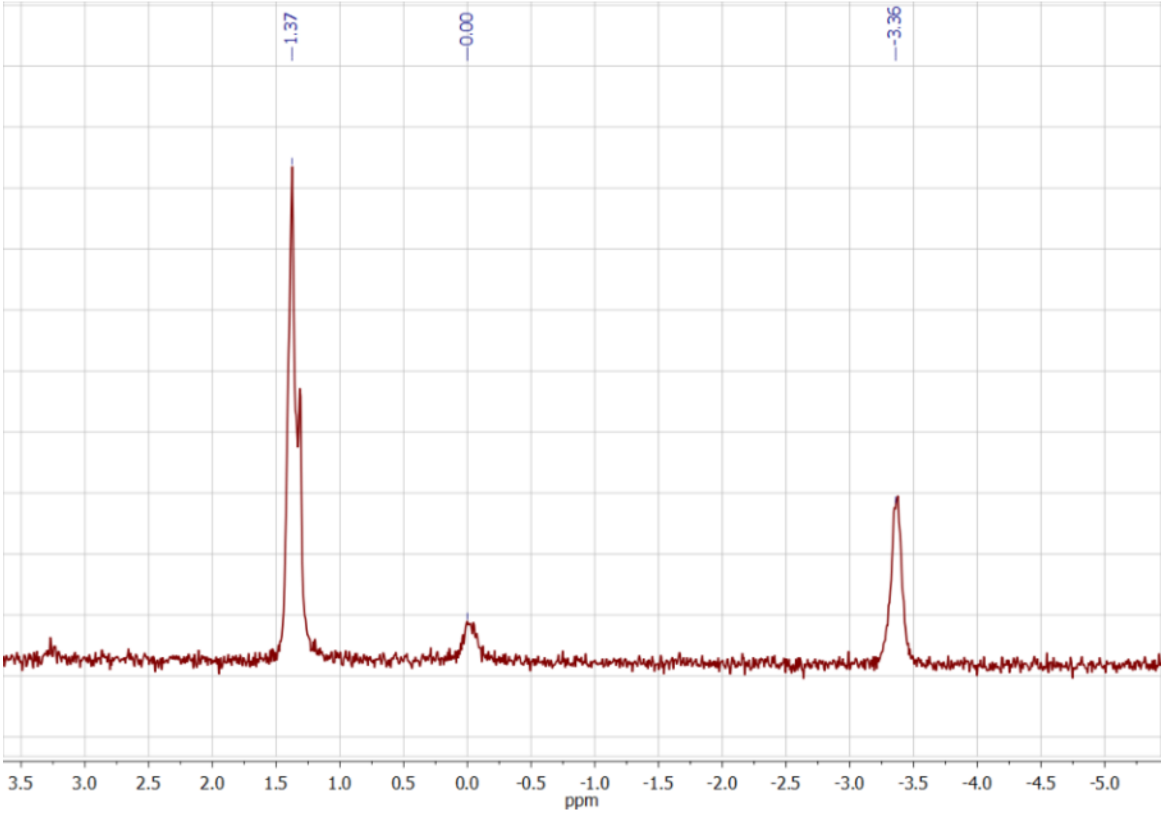

Figure S3-1: ^{1}H NMR of $NaCH_2SiMe_3$ in [D12]cyclohexane:

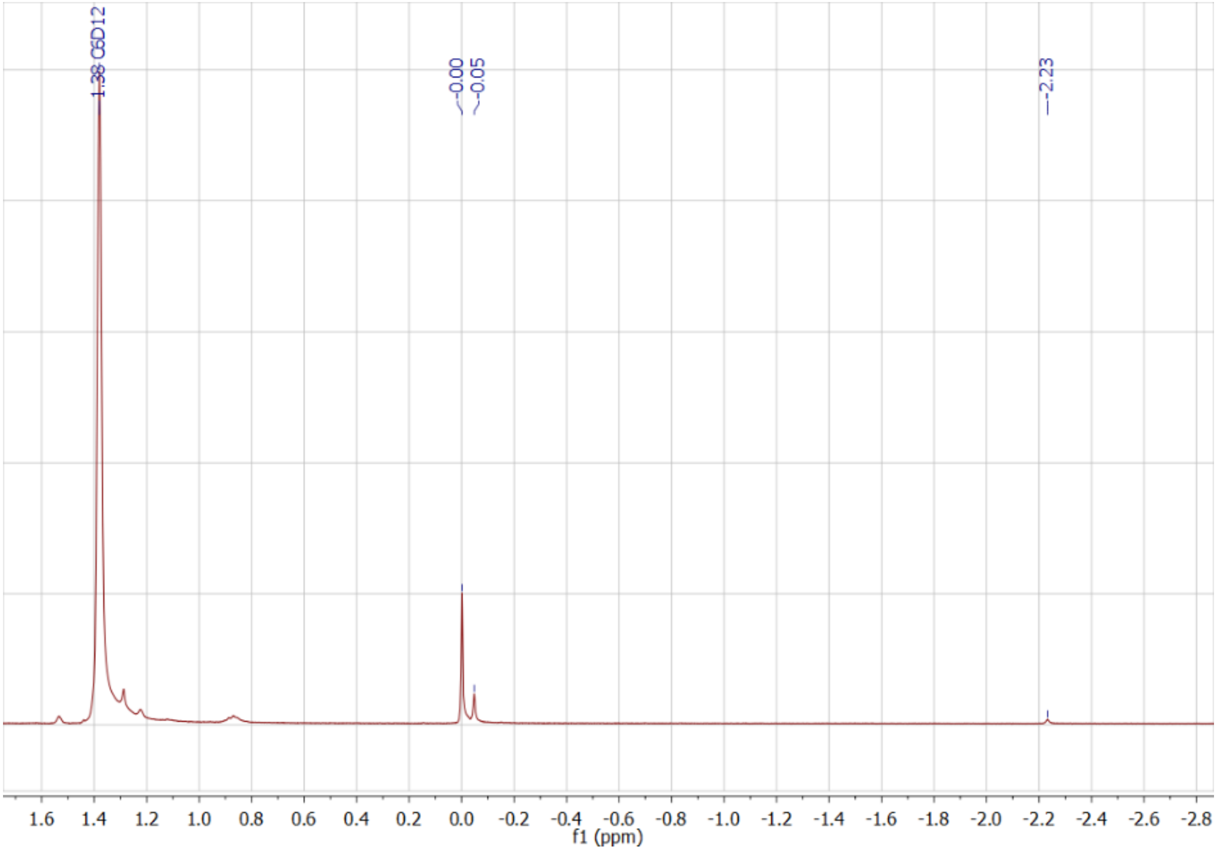

Figure S3-2: ^{1}H NMR of mixture of **CH_3@1** and $NaCH_2SiMe_3$ in [D12]cyclohexane:

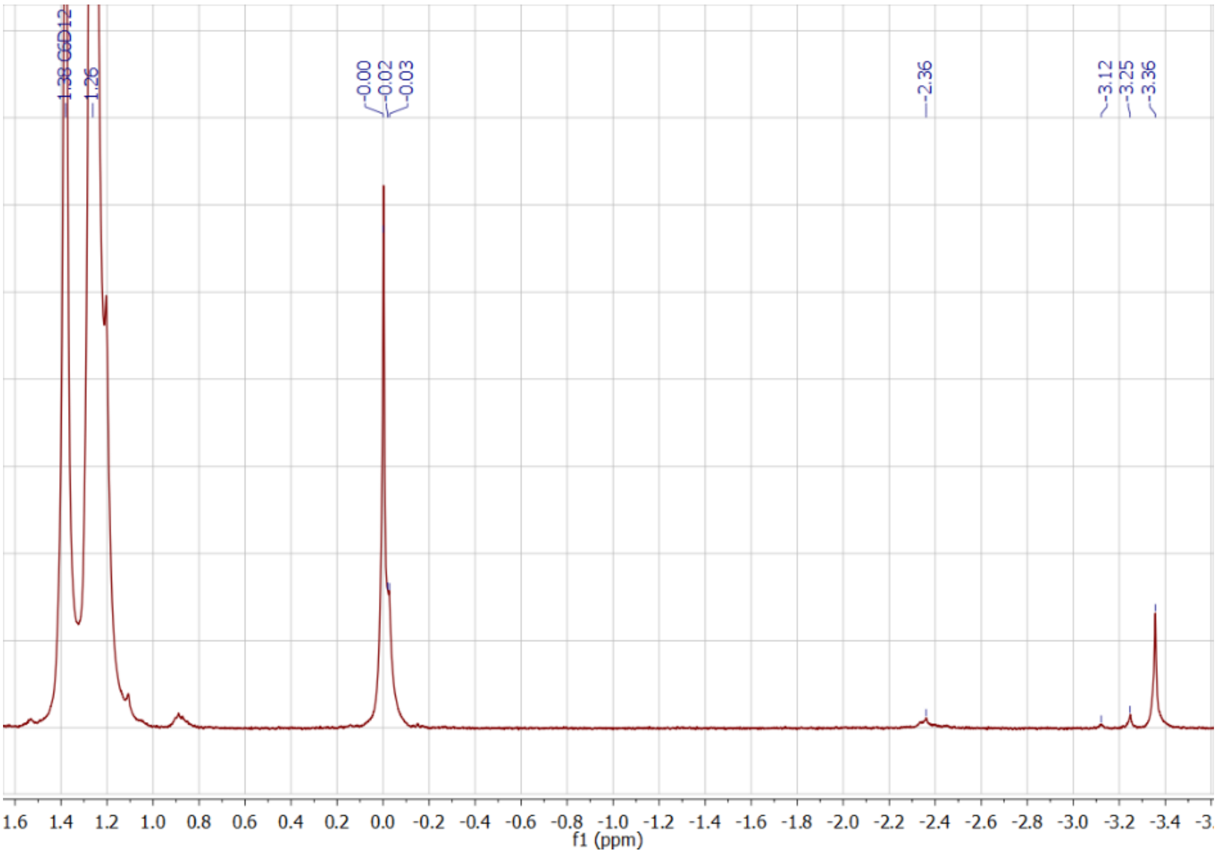

Figure S3-3: ^{1}H NMR of mixture of **CH_3@1** (3.23 ppm) and CH_3@Na_{13}(O*t*Bu)$_{11}$(CH_2SiMe_3) (3.13 ppm) in [D6]benzene:

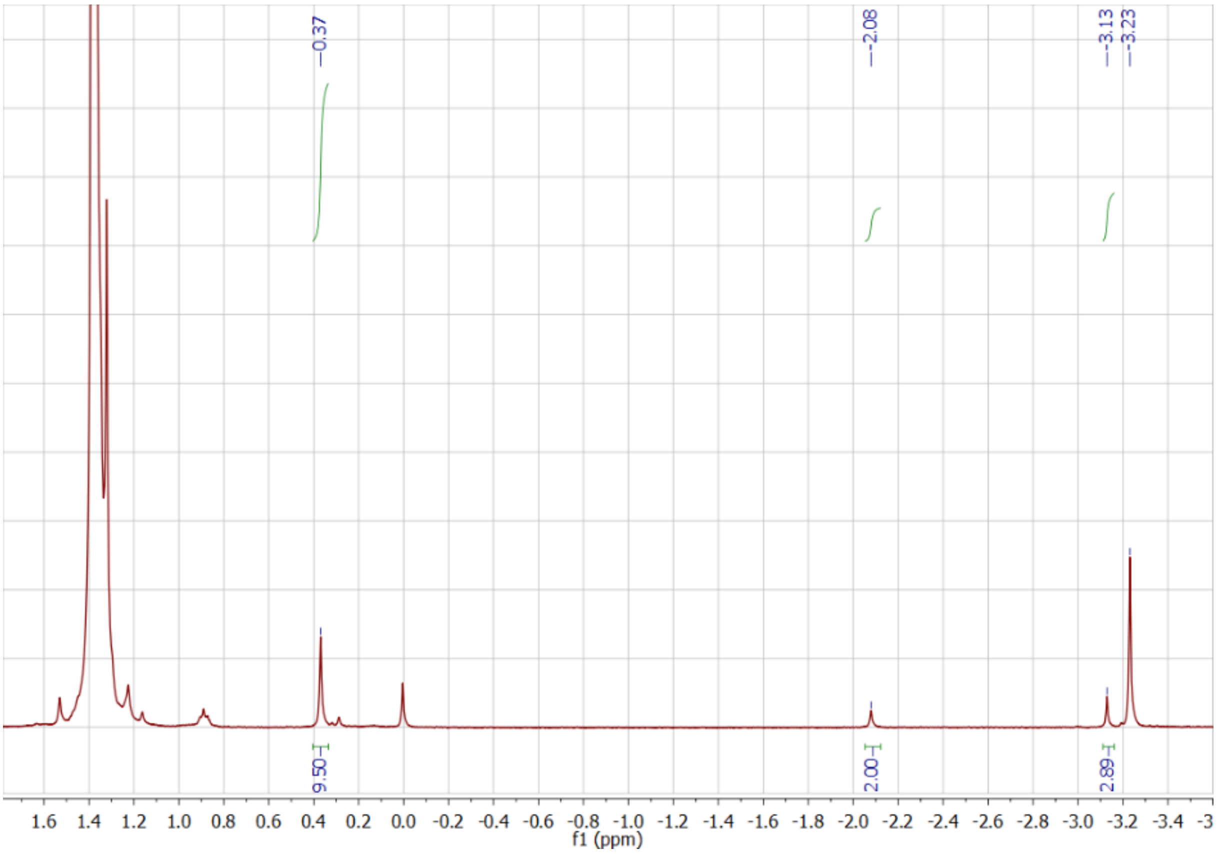

Figure S3-4: ^{1}H NMR of NaOMeCyc in [D6]benzene:

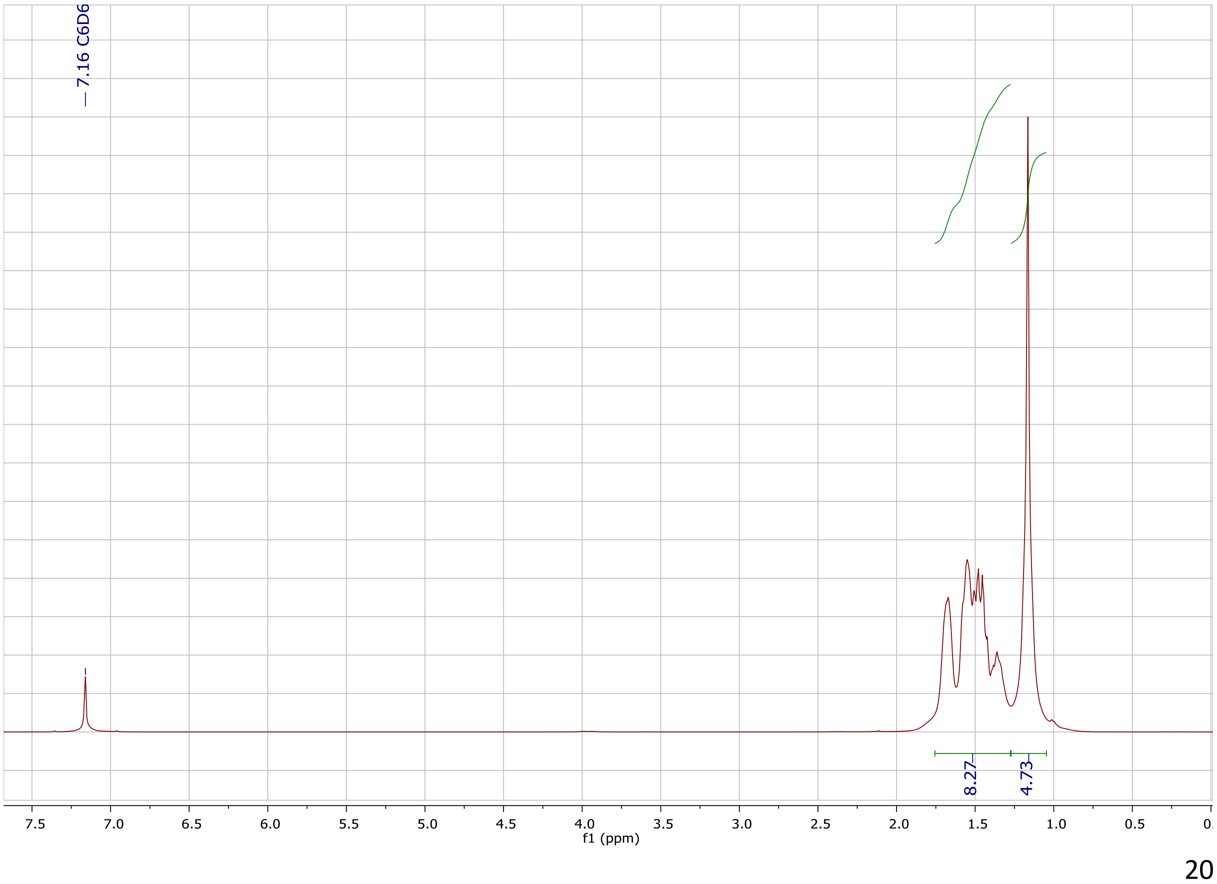

Figure S3-4: ^1H-^1H COSY NMR of NaOMeCyc in [D6]benzene:

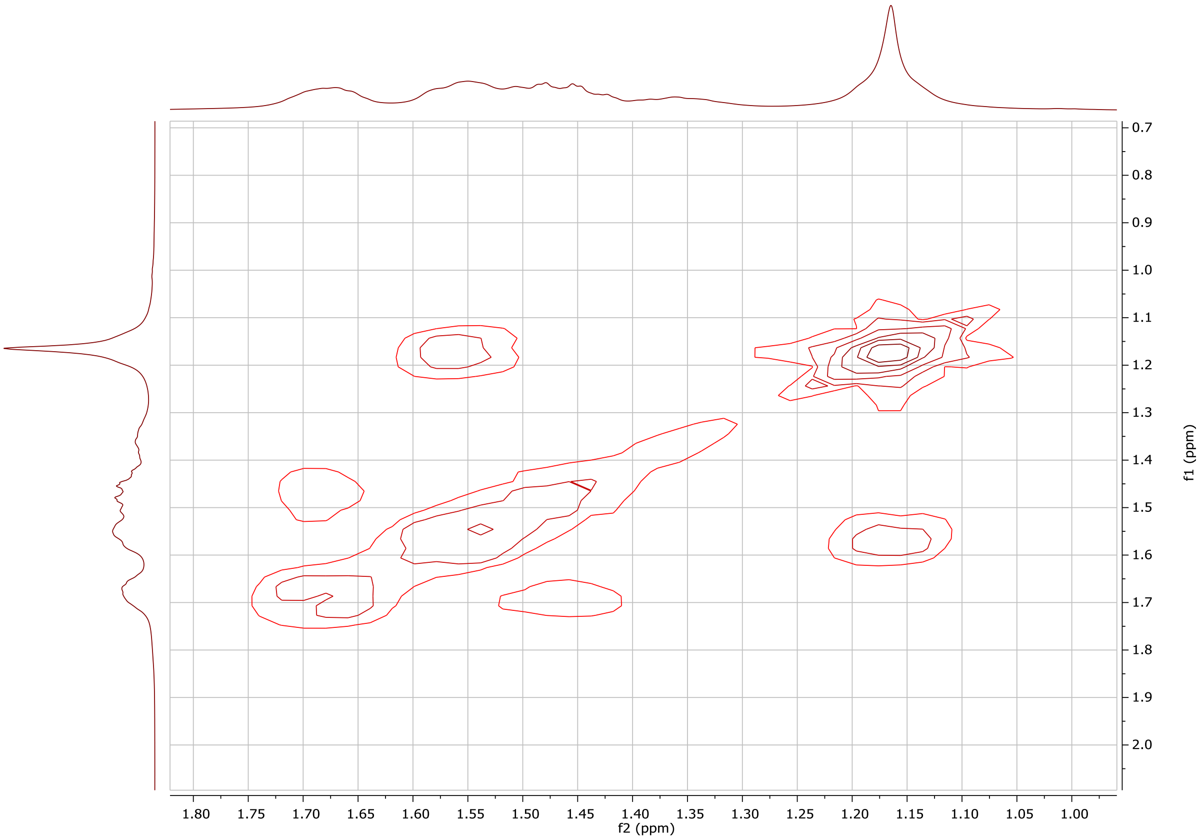

Figure S3-5: ^{13}C NMR of NaOMeCyc in [D6]benzene:

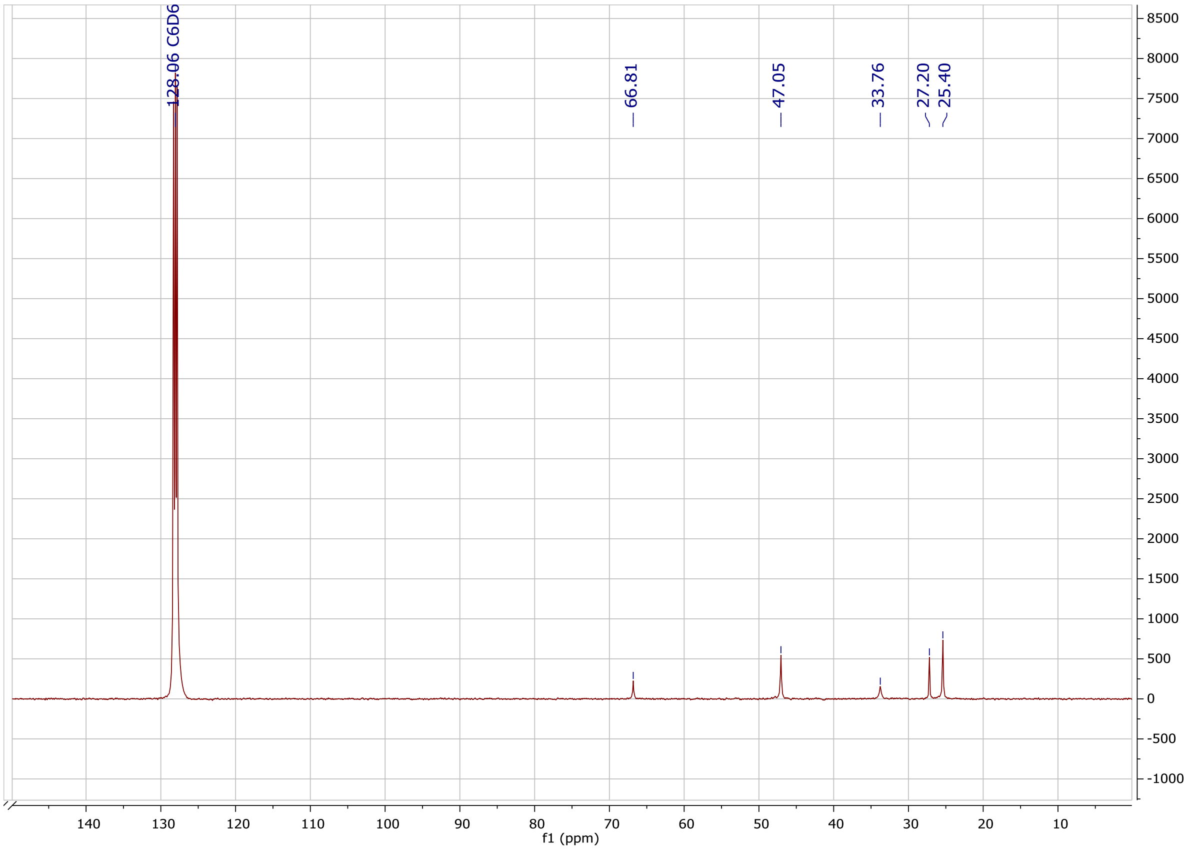

Figure S3-6: ^{1}H-^{13}C HSQC NMR of NaOMeCyc in [D6]benzene:

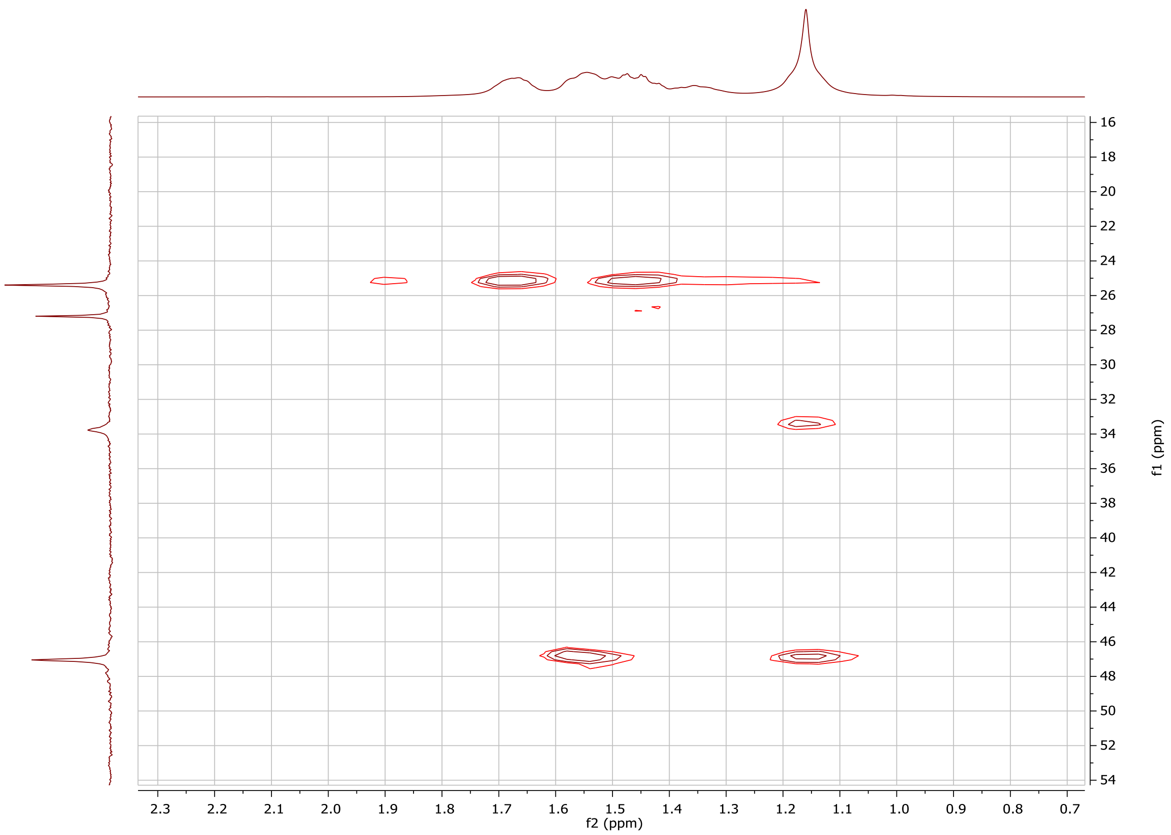

Figure S3-7: ^{13}C NMR of $CH_3@Na_{13}(OMeCyc)_{12}$ in [D6]benzene:

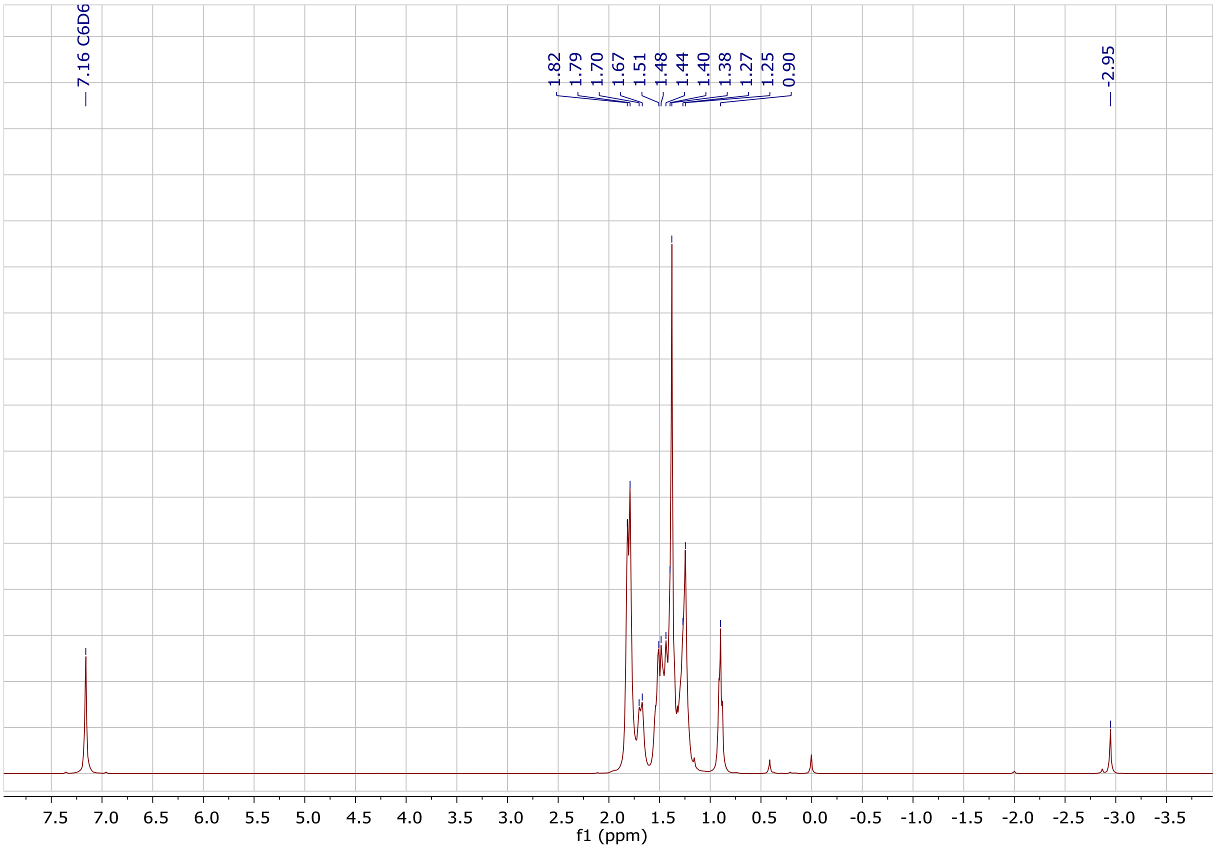

Figure S3-6: ^{1}H NMR of mixtures of **CH_3@1** and NaOMeCyc in [D6]benzene (left, signal distance ~9.6 Hz) and [D12]cyclohexane (right, signal distance ~4.6 Hz):

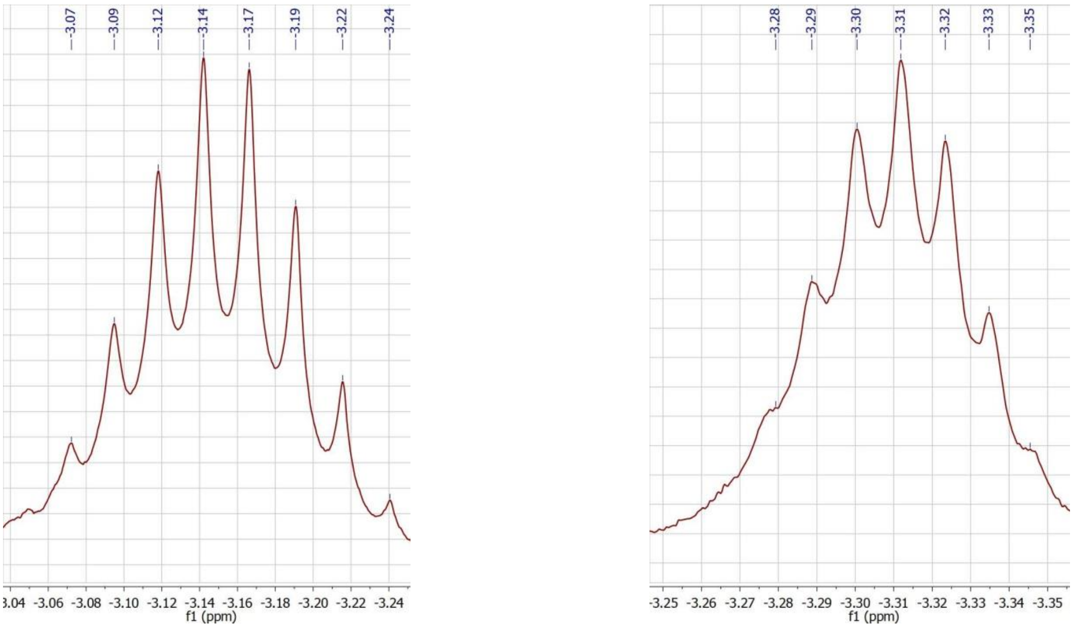

Figure S3-7: Development over time of mixtures of **CH_3@1** and CH_3@Na_{13}(OMeCyc)$_{12}$ observed by ^{1}H NMR in [D6]benzene at 50°C:

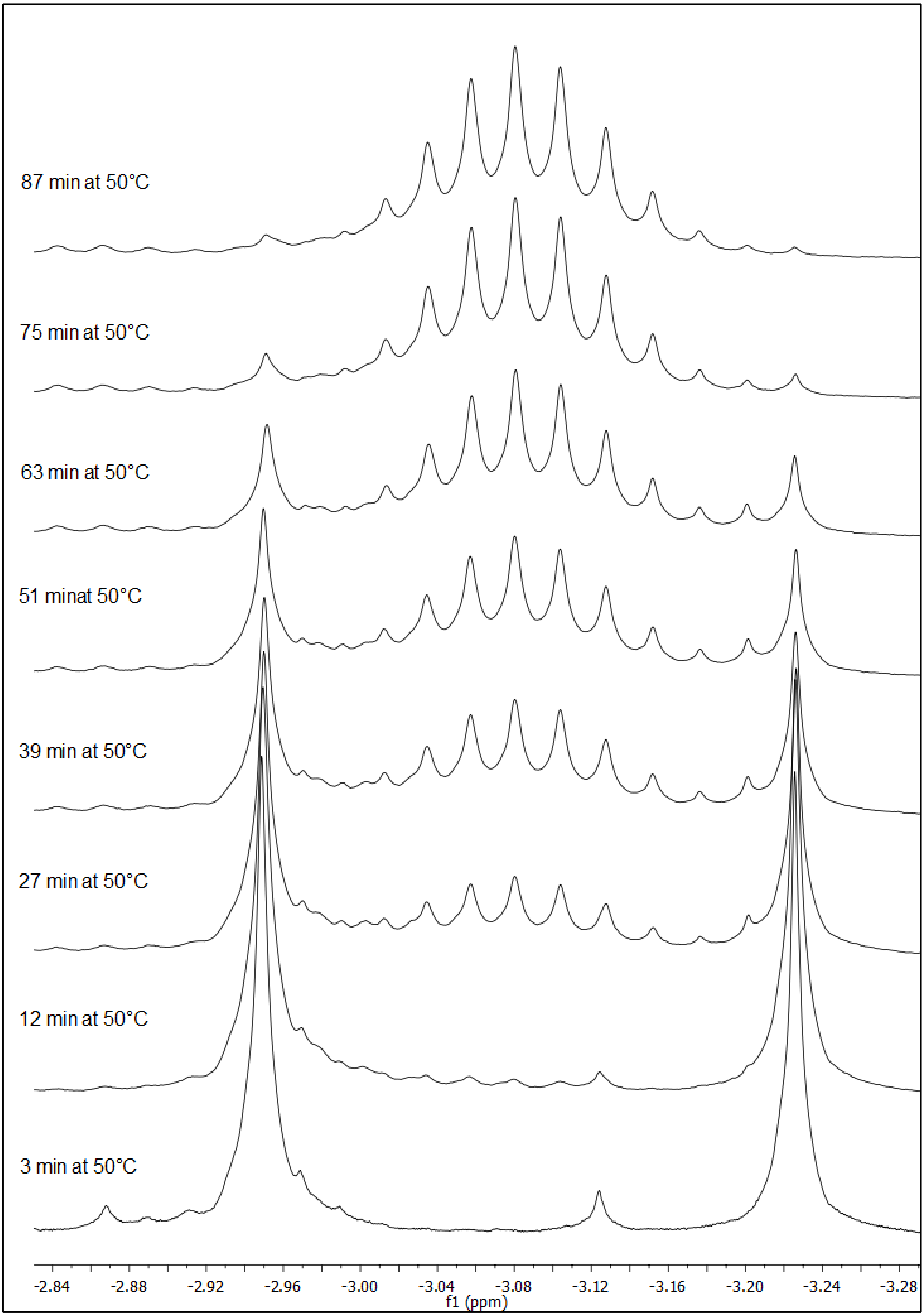

Figure S3-8: Development over time of mixtures of **CH_3@1** and CH_3@Na_{13}(OMeCyc)$_{12}$ with additional NaO*t*Bu added observed by ^{1}H NMR in [D6]benzene at 50°C:

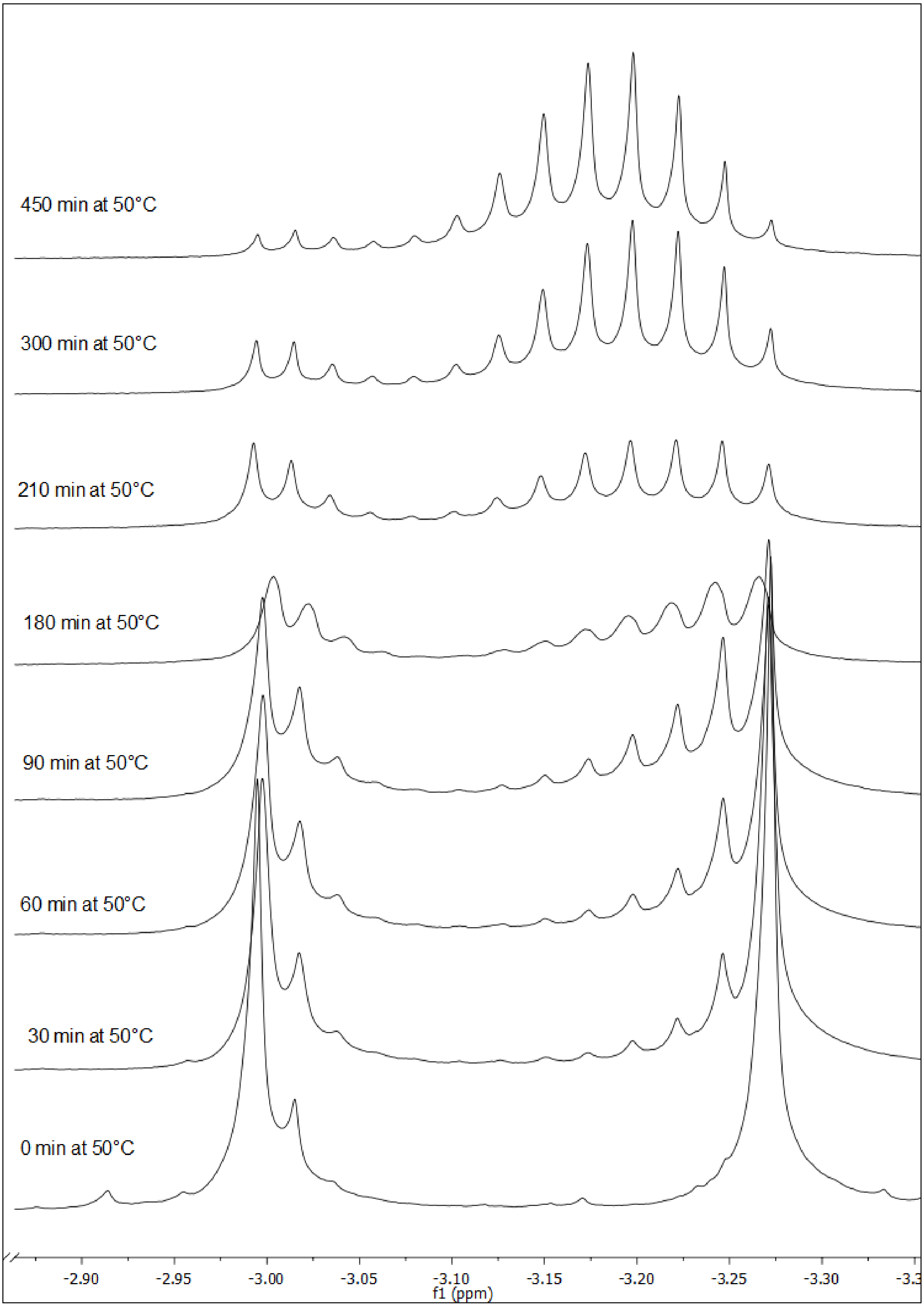

Figure S4-1: ^{1}H NMR of **CN@1** in [D6]benzene:

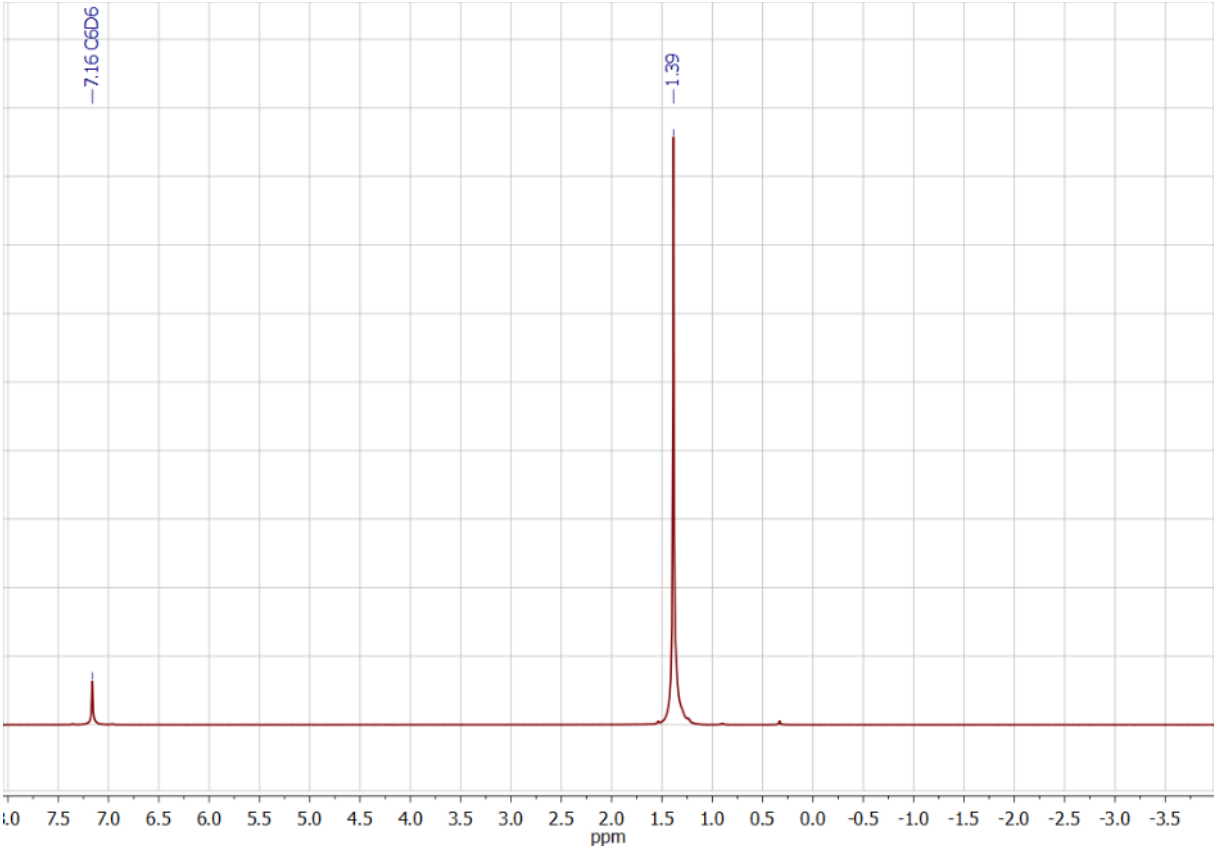

Figure S4-2: ^{1}H NMR of **^{13}CN@1** in [D6]benzene:

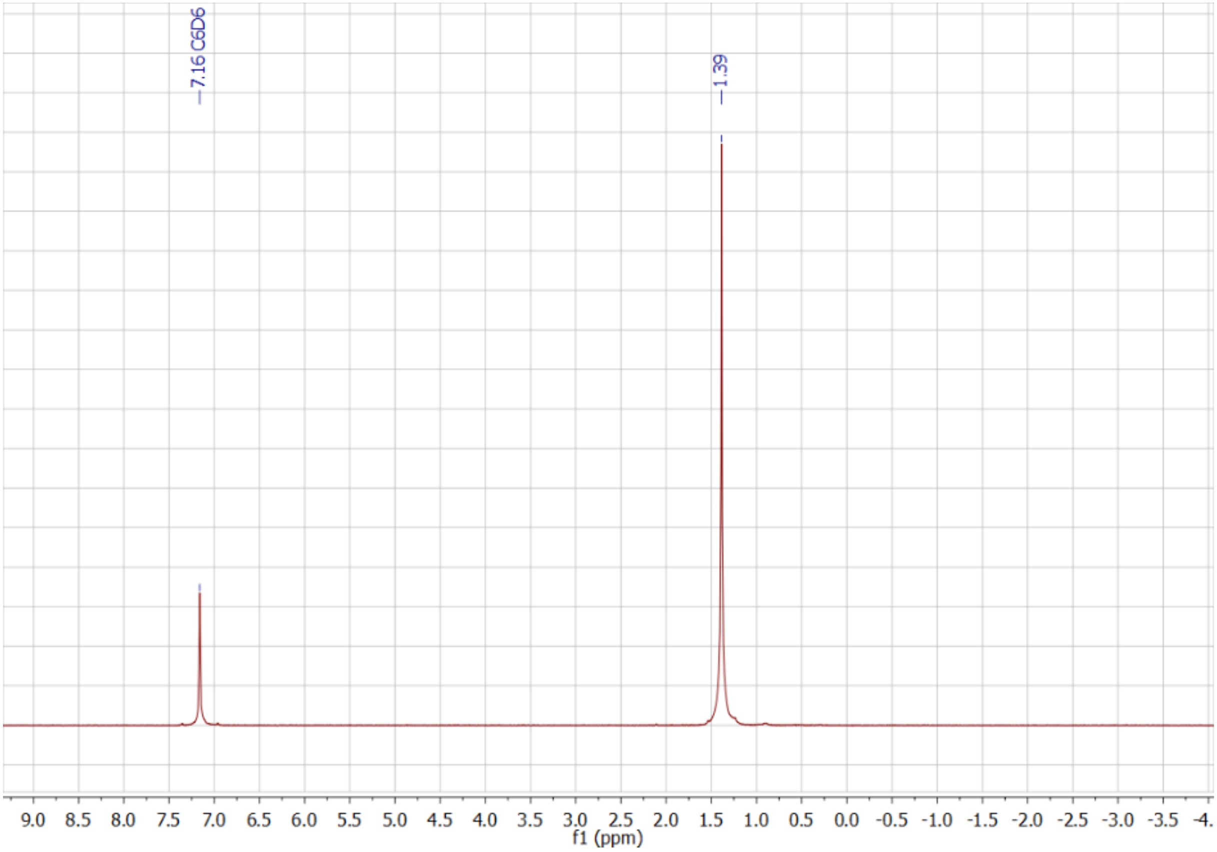

Figure S4-3: ^{13}C NMR of **^{13}CN@1** in [D6]benzene:

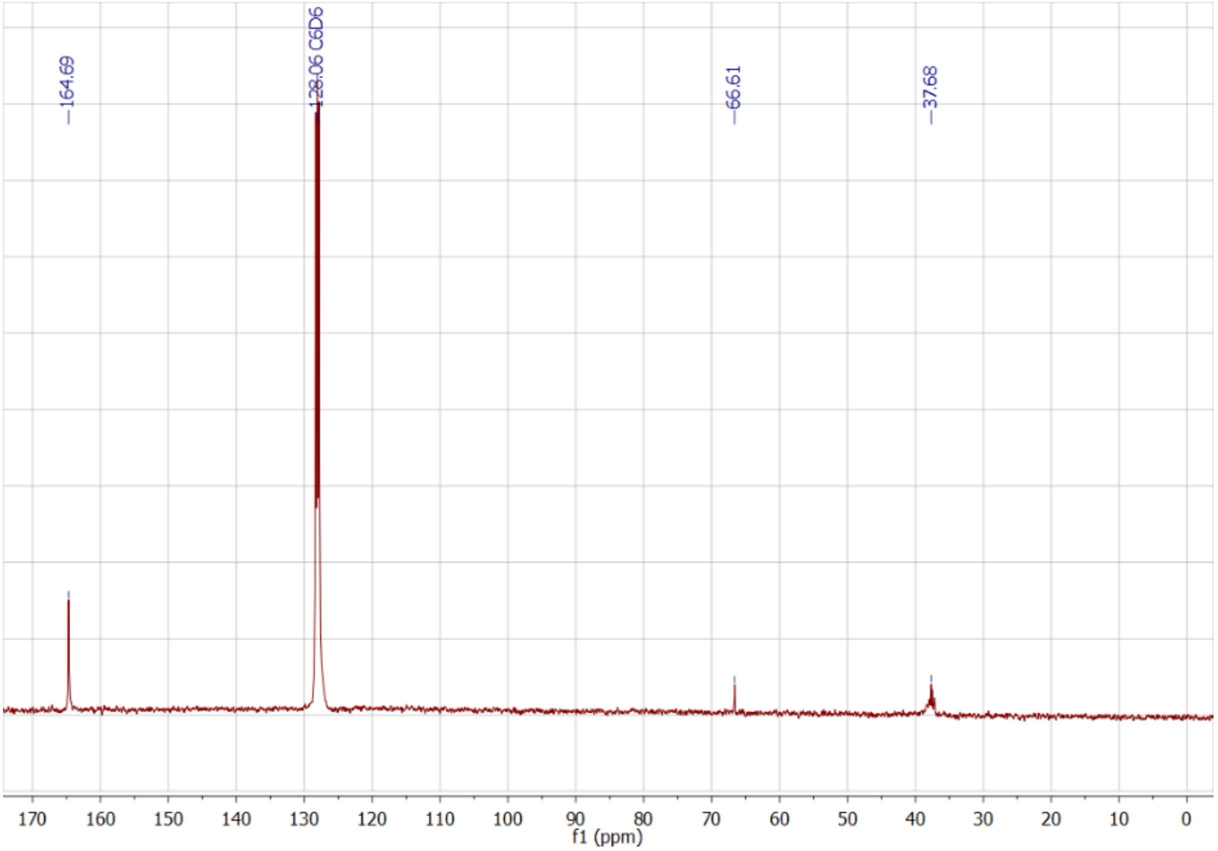

Figure S5-1: ^{1}H NMR of **2-F** in [D6]benzene:

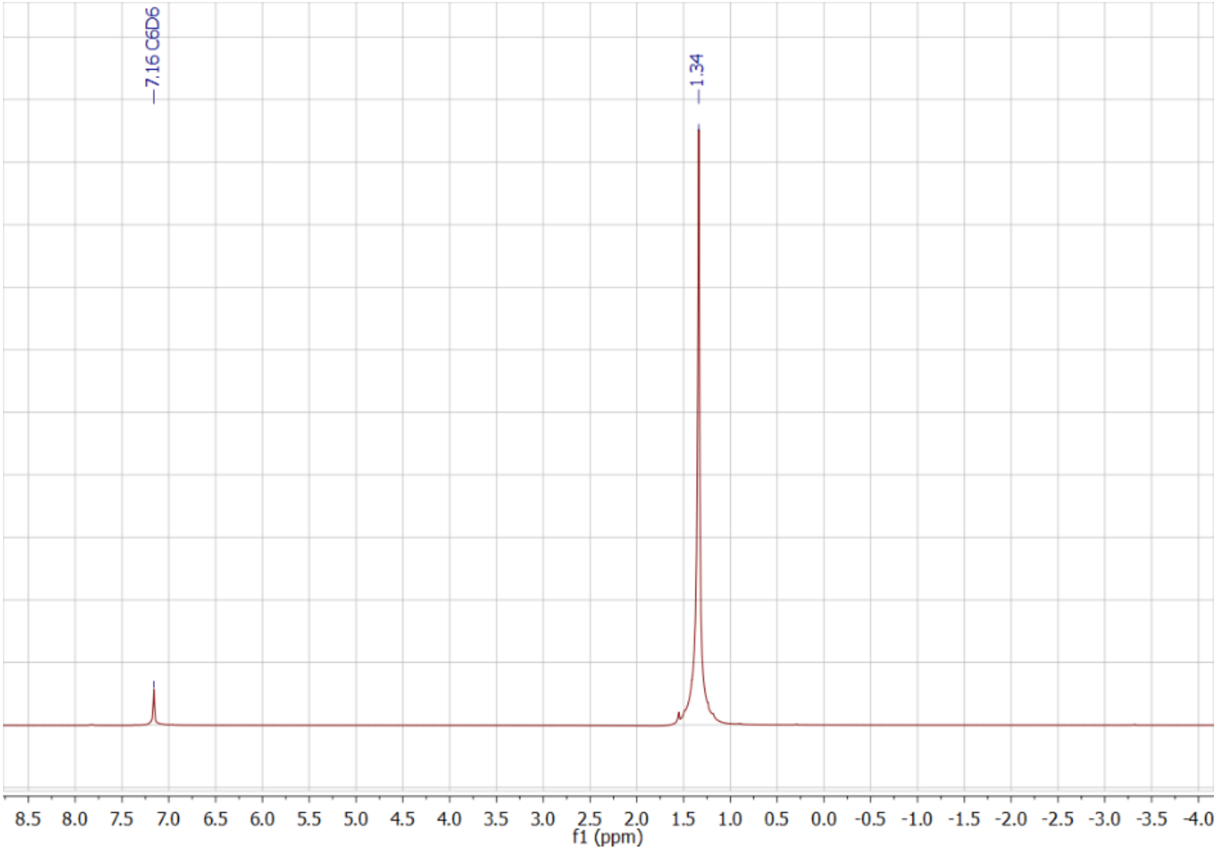

Figure S5-2: ^{19}F NMR of **2-F** in [D6]benzene:

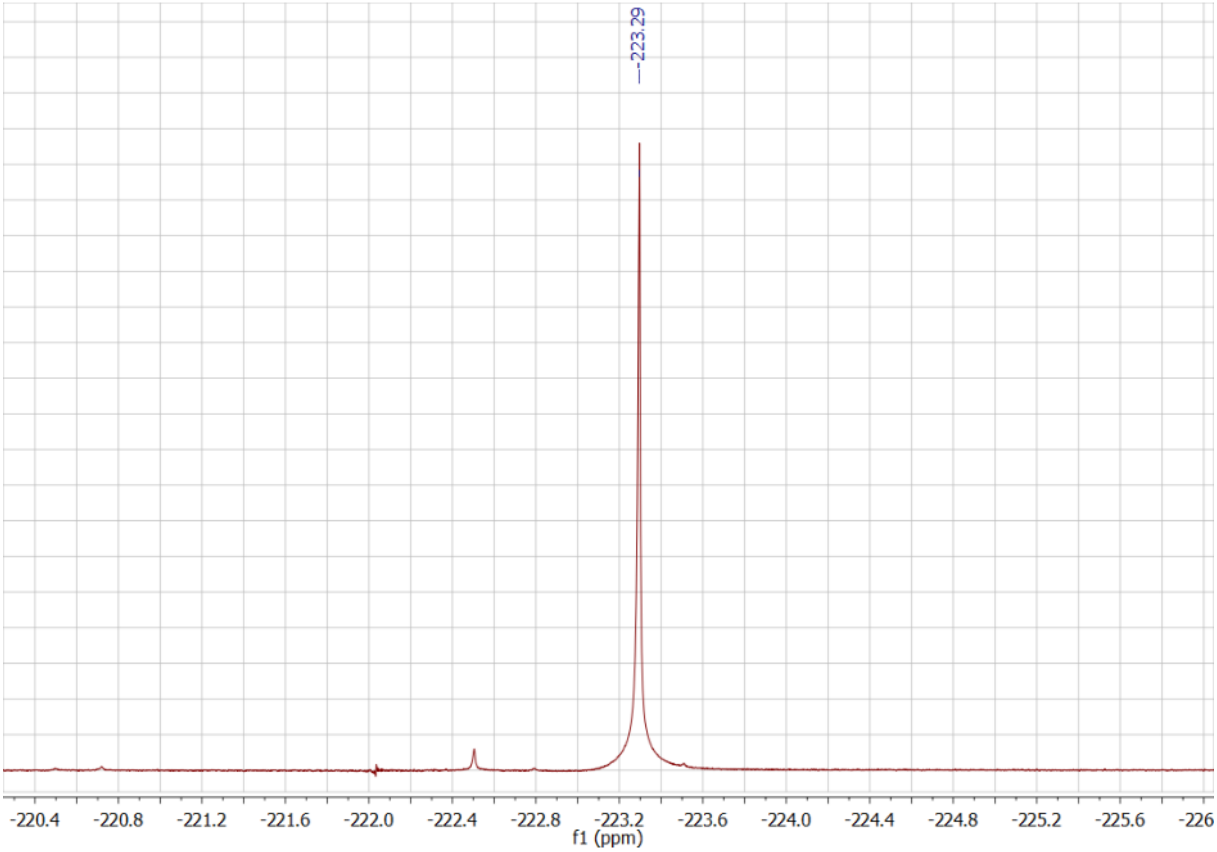

Figure S6-1: ^{1}H NMR of **Cl@1** in [D6]benzene:

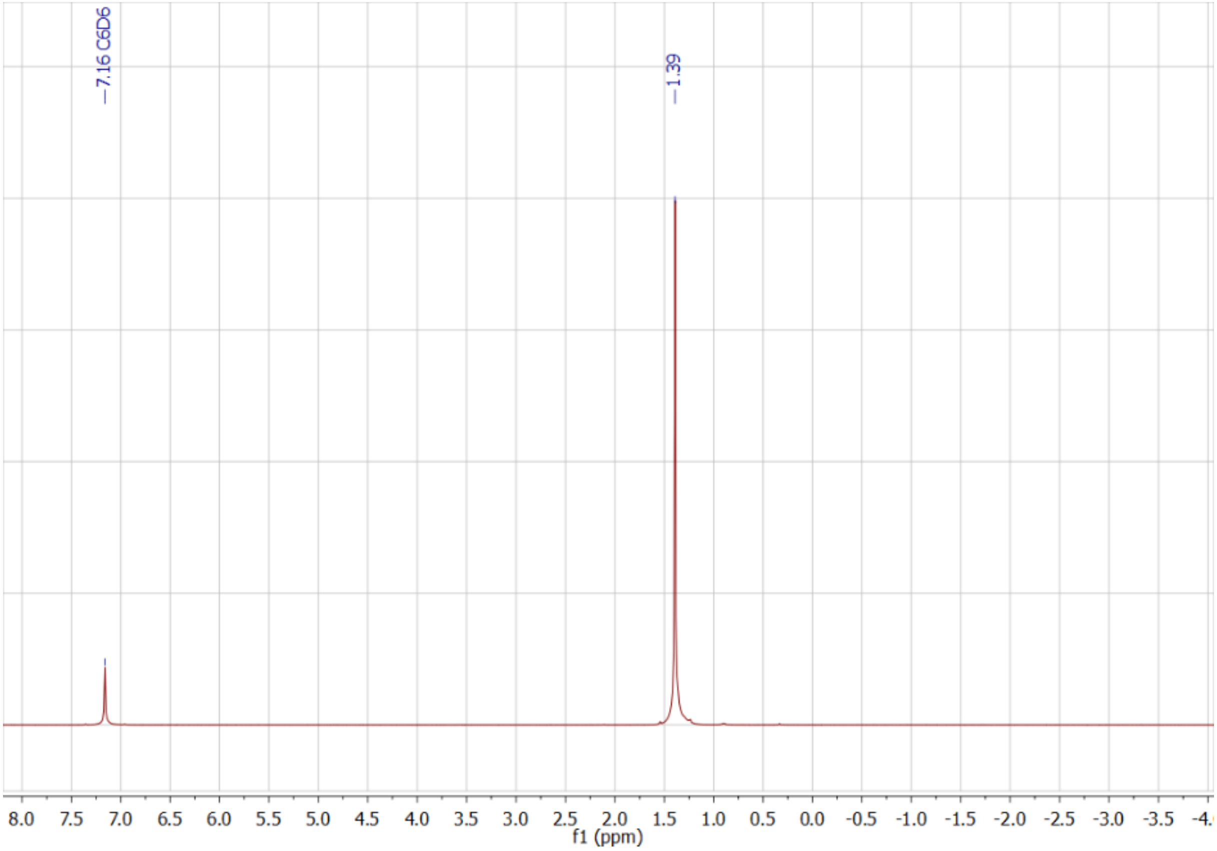

Figure S6-2: ^{35}Cl NMR of **Cl@1** in [D6]benzene:

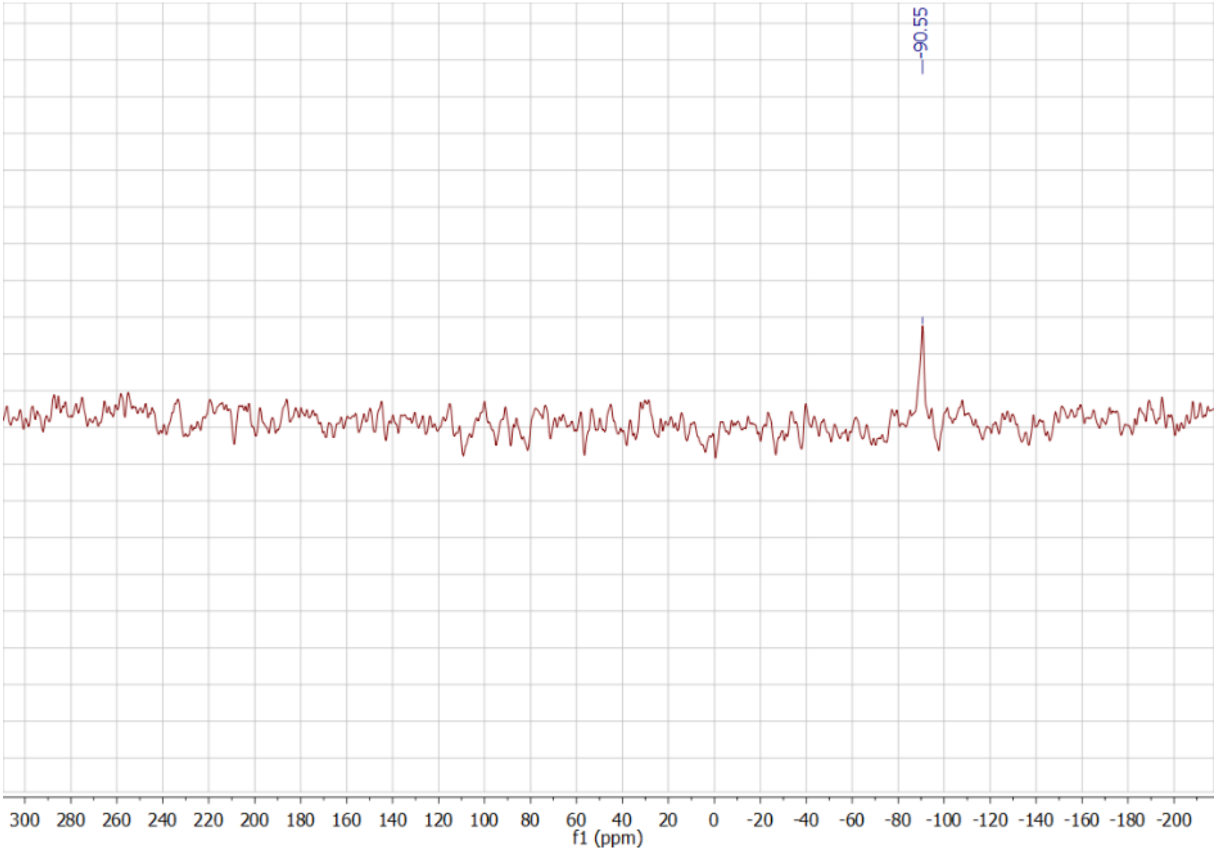

Figure S7-1: ^{1}H NMR of **Br@1** in [D6]benzene:

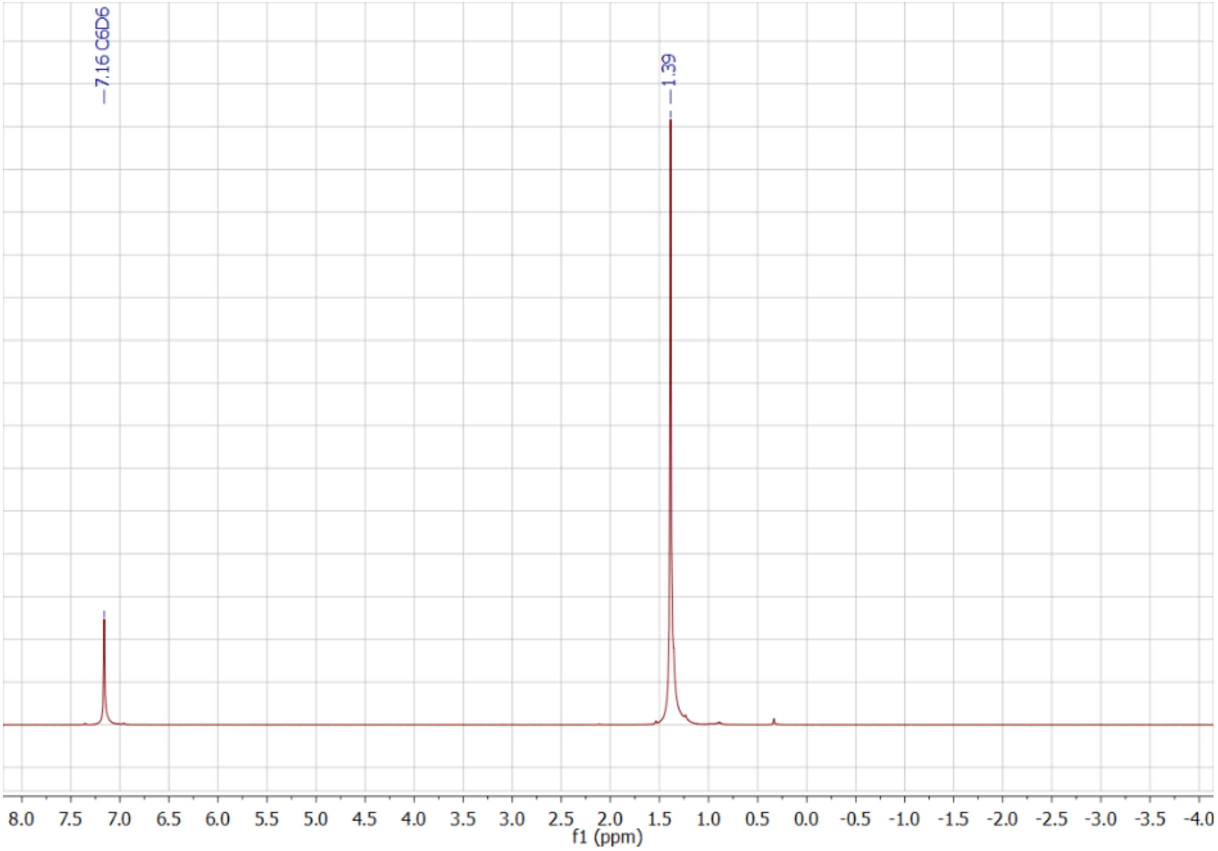

Figure S7-2: ^{79}Br NMR of **Br@1** in [D6]benzene:

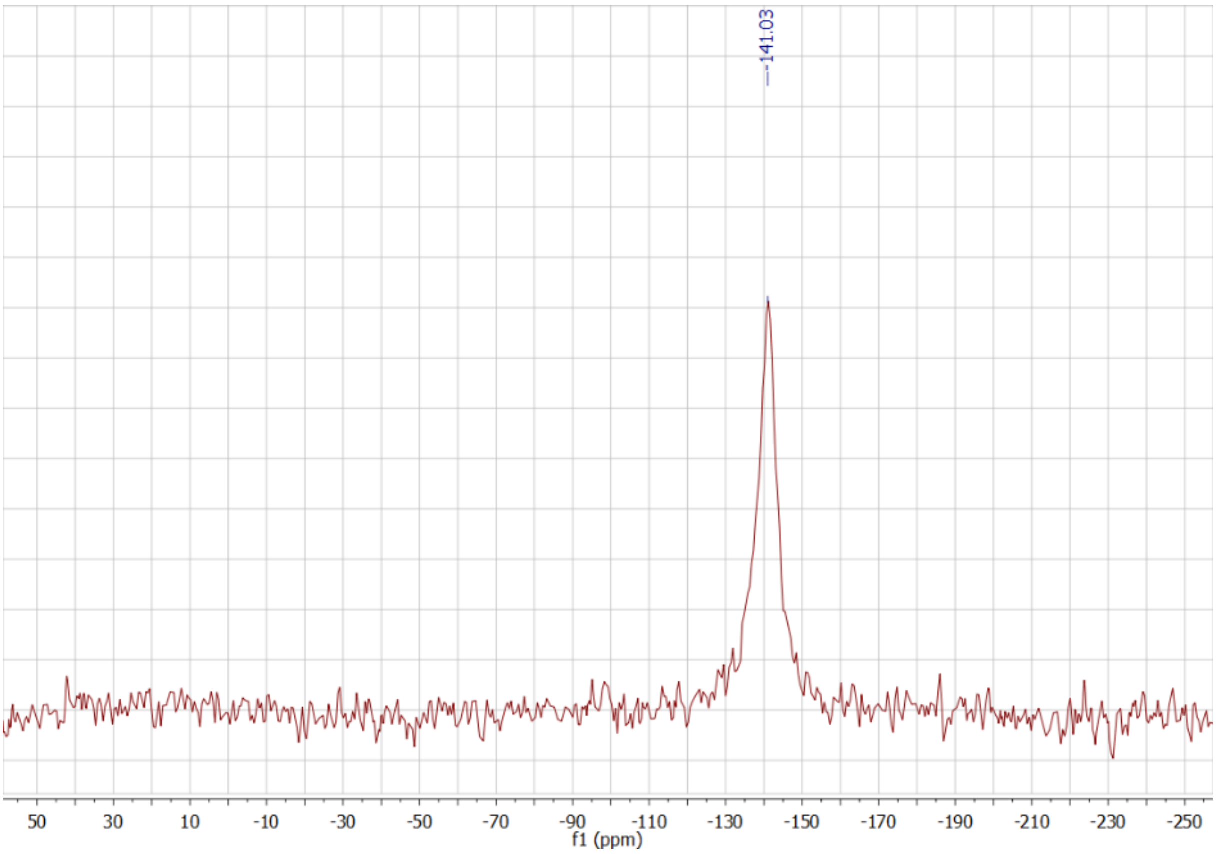

Figure S8-1: ^{1}H NMR of **I@1** in [D6]benzene:

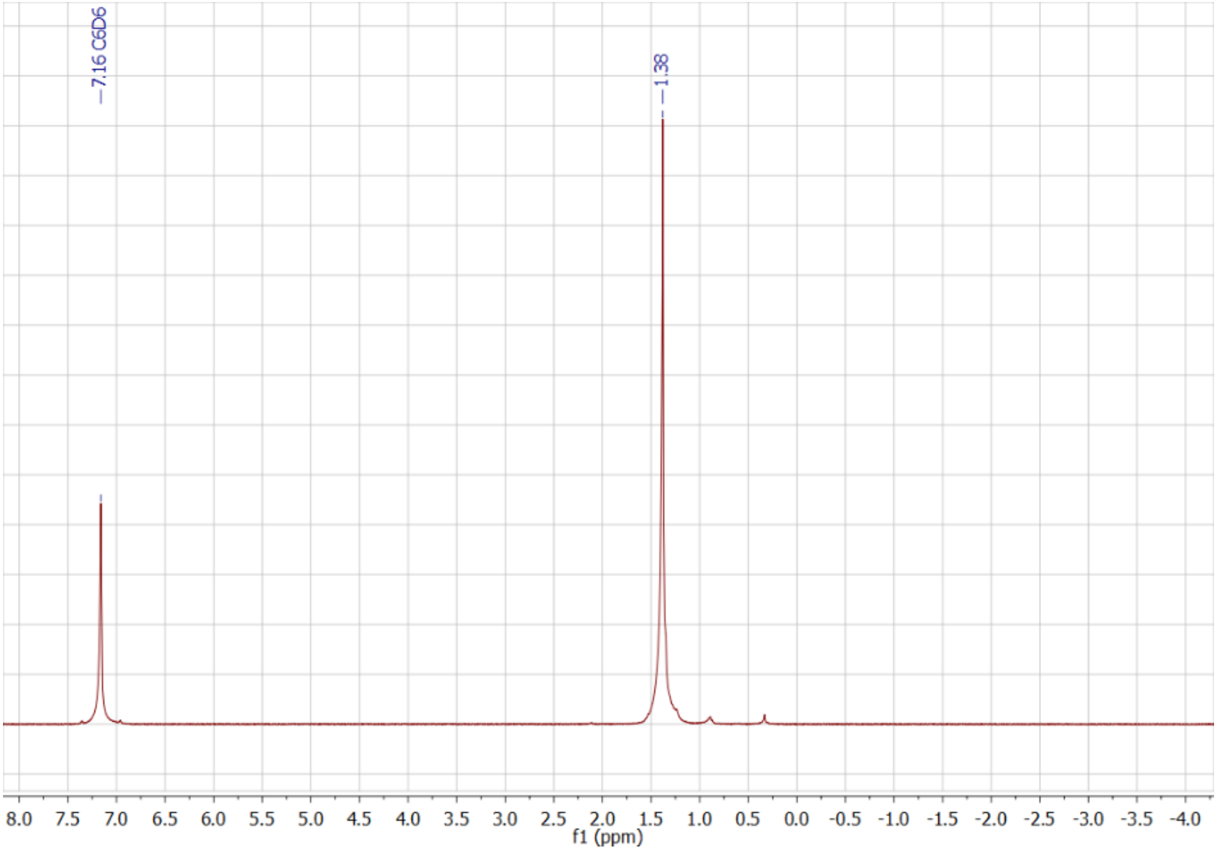

Figure S8-2: ^{127}I NMR of **I@1** in [D6]benzene:

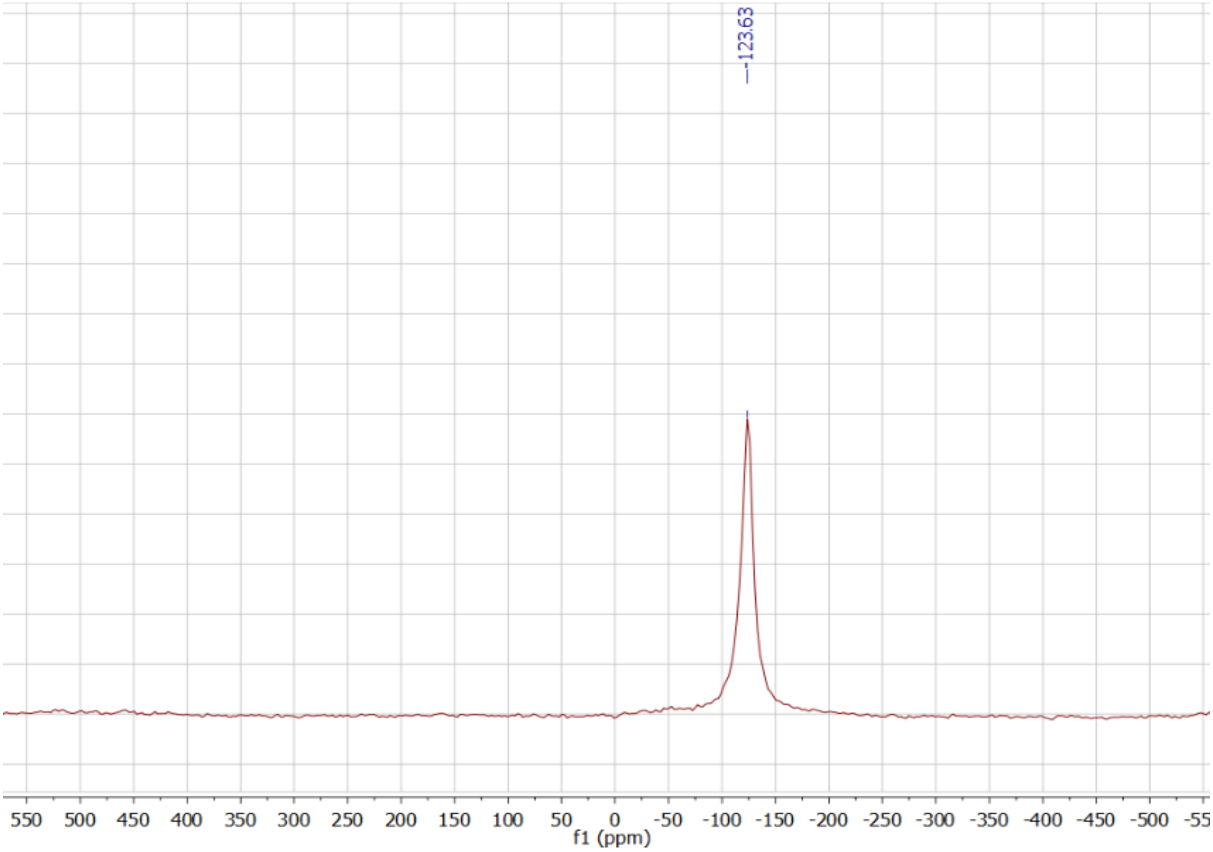

Figure S9-1: ^{1}H NMR of **OCN@1** in [D6]benzene:

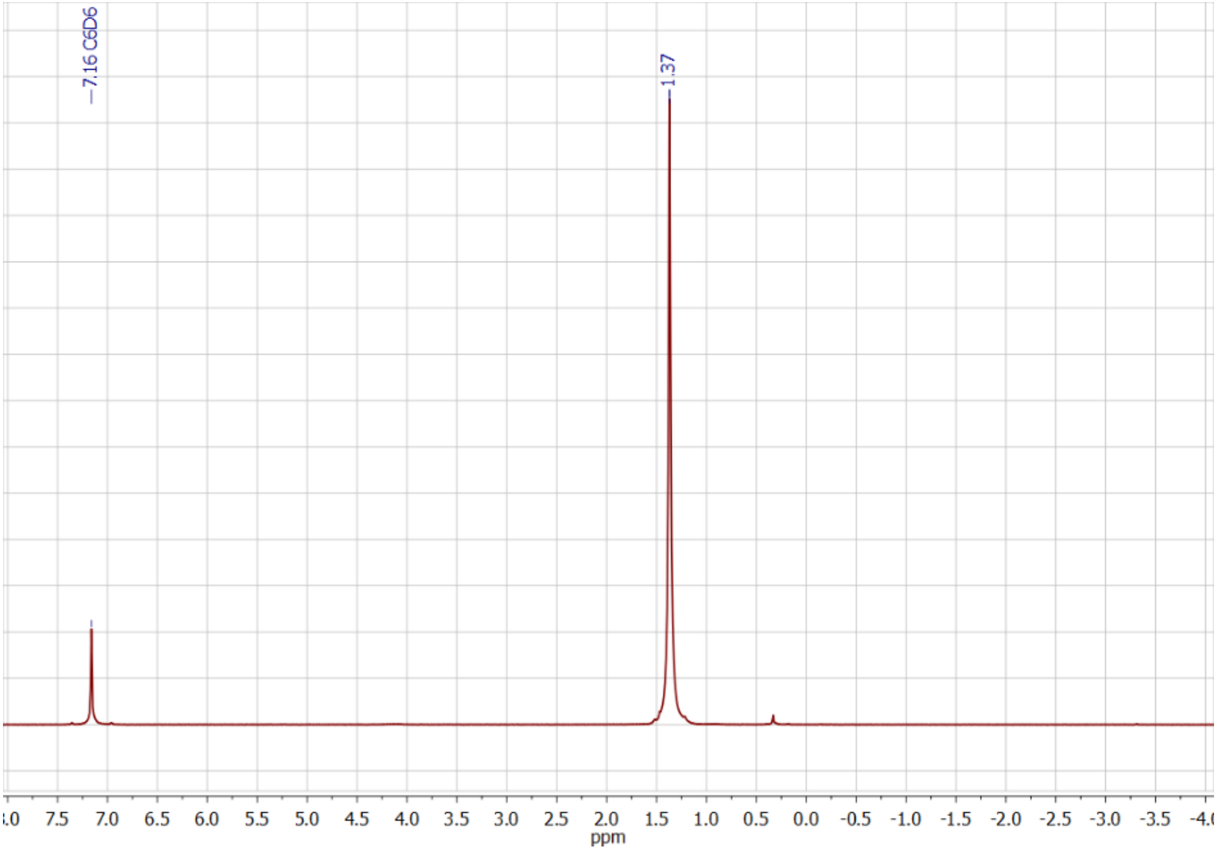

Figure S10-1: ^{1}H NMR of **SCN@1** in [D6]benzene:

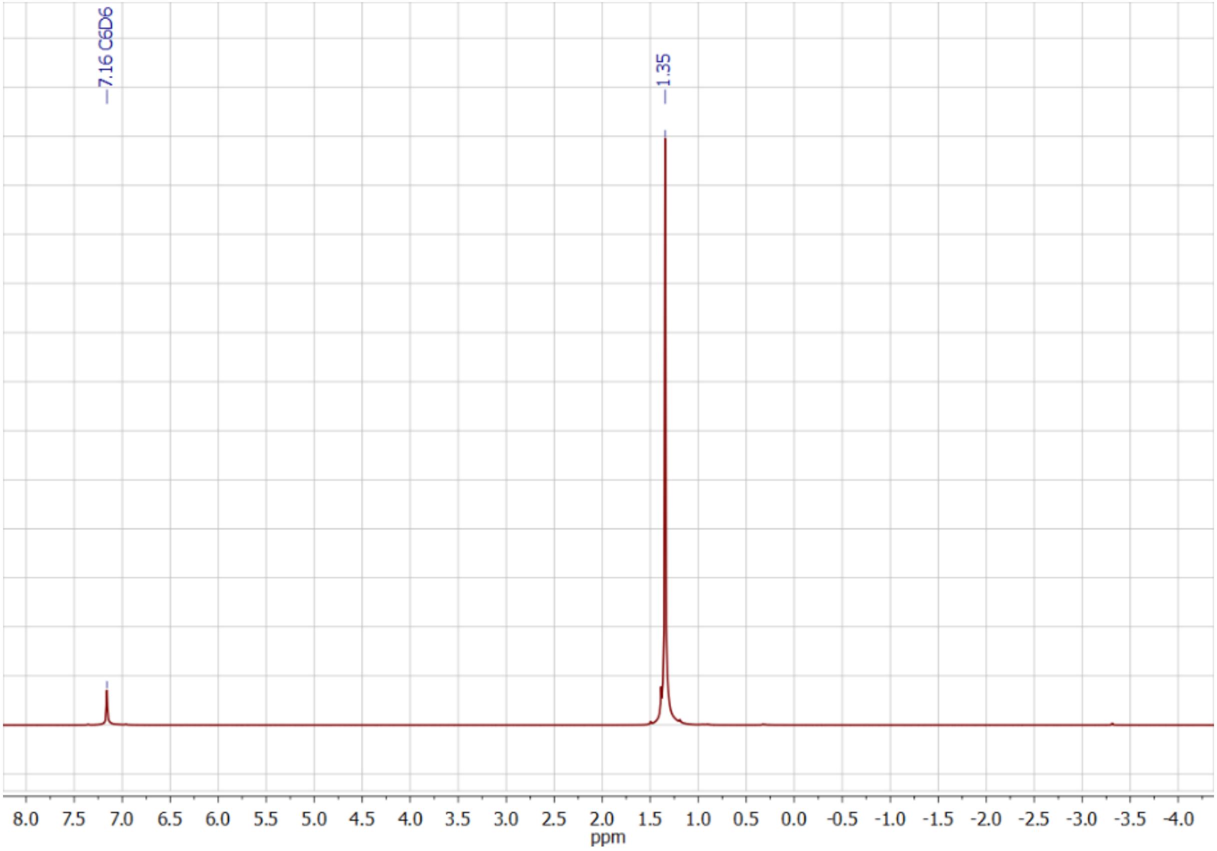

Figure S10-2: ^{13}C NMR of **SCN@1** in [D6]benzene: ?

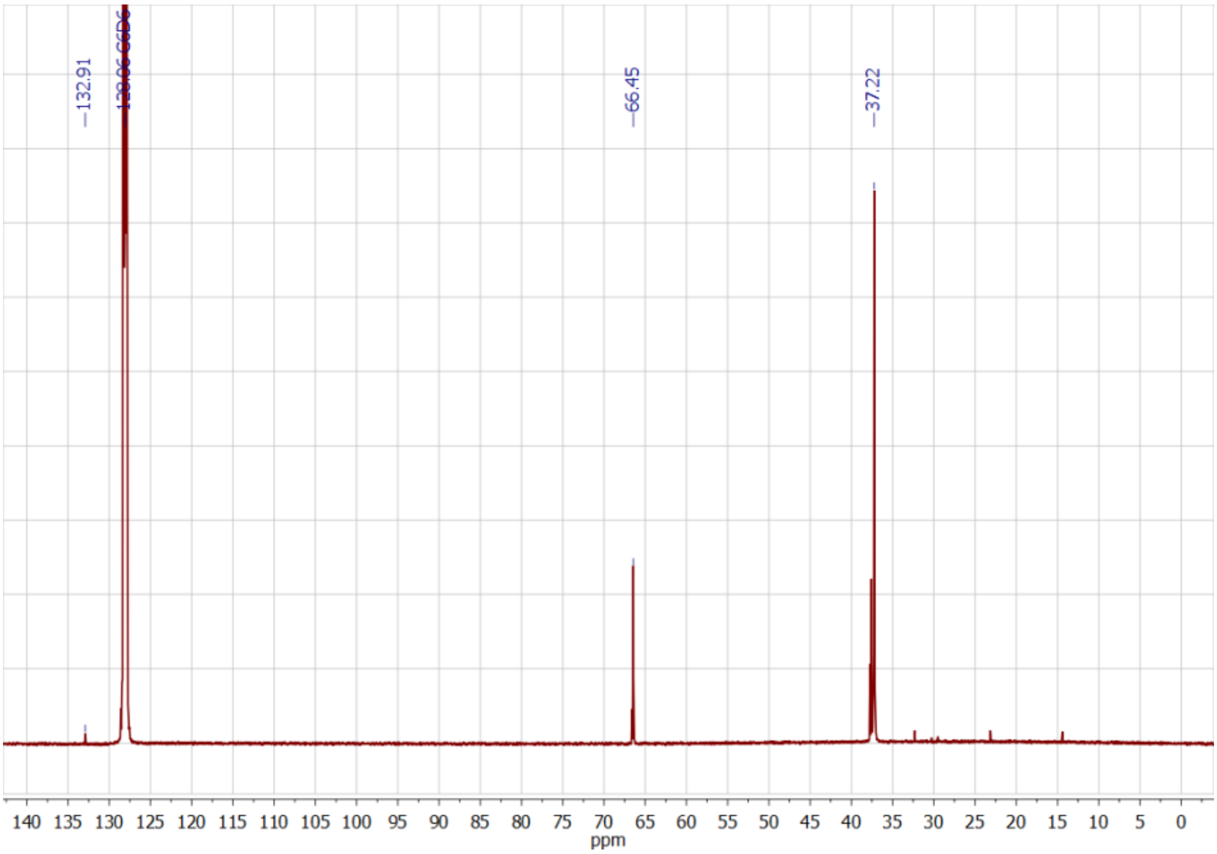

Figure S11-1: ^{1}H NMR of **N_3@1** in [D6]benzene:

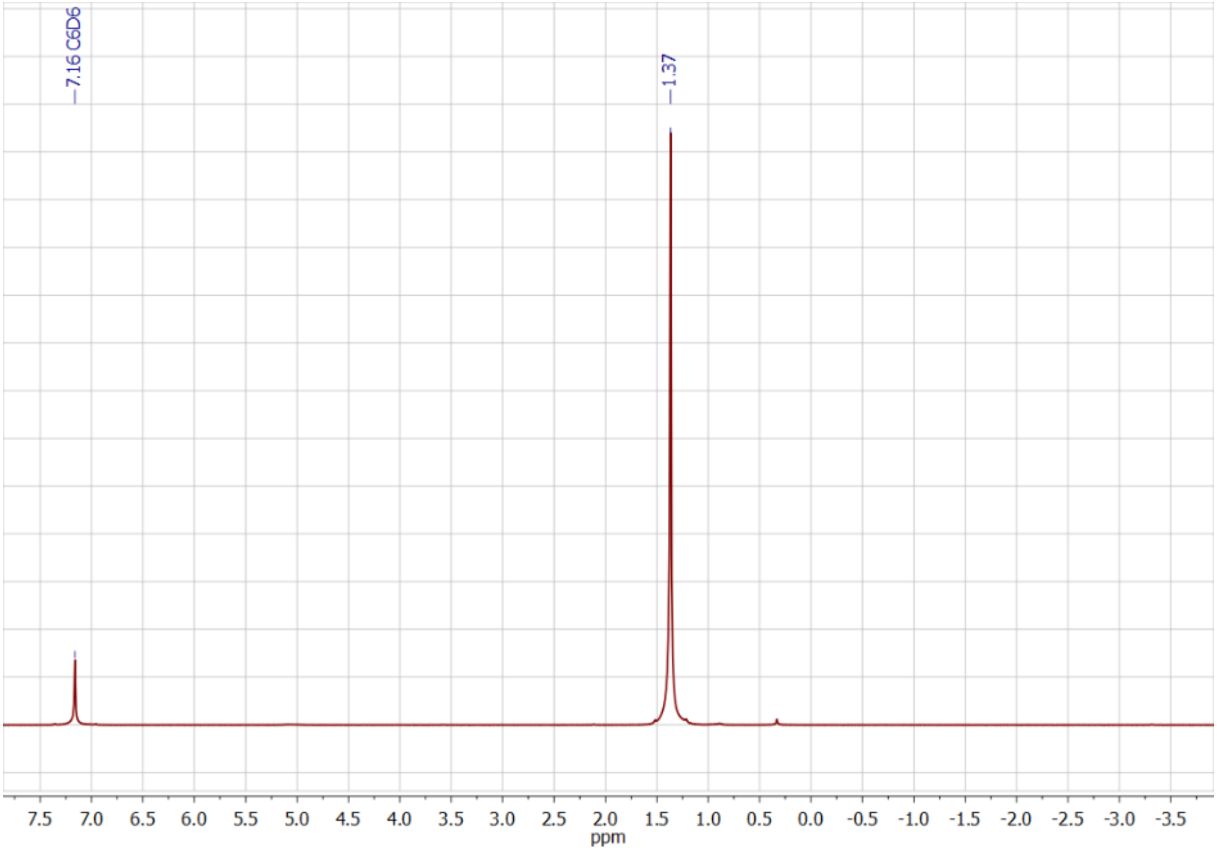

Figure S11-2: ^{1}H NMR of **1-$^{15}N_3$@1** in [D6]benzene:

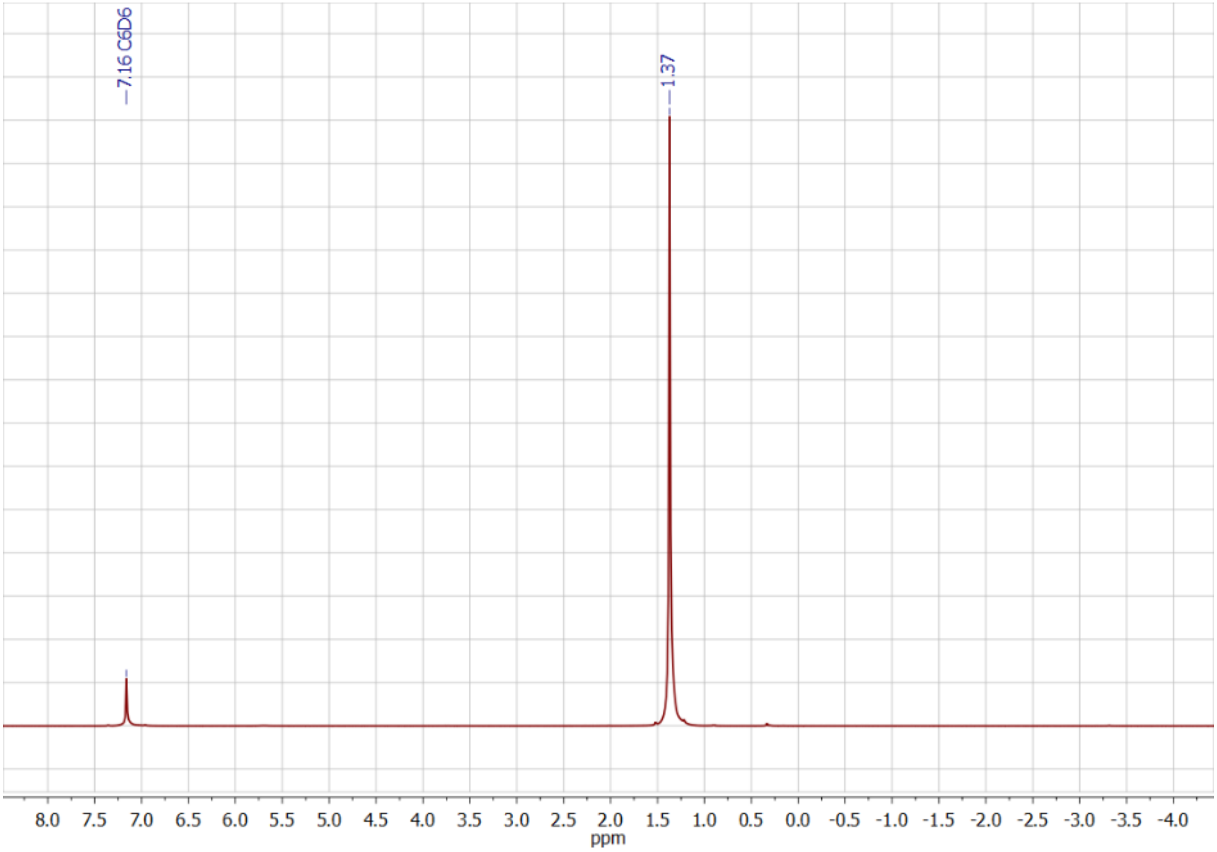

Figure S12-1: ^{1}H NMR of **NO_3@1** in [D6]benzene:

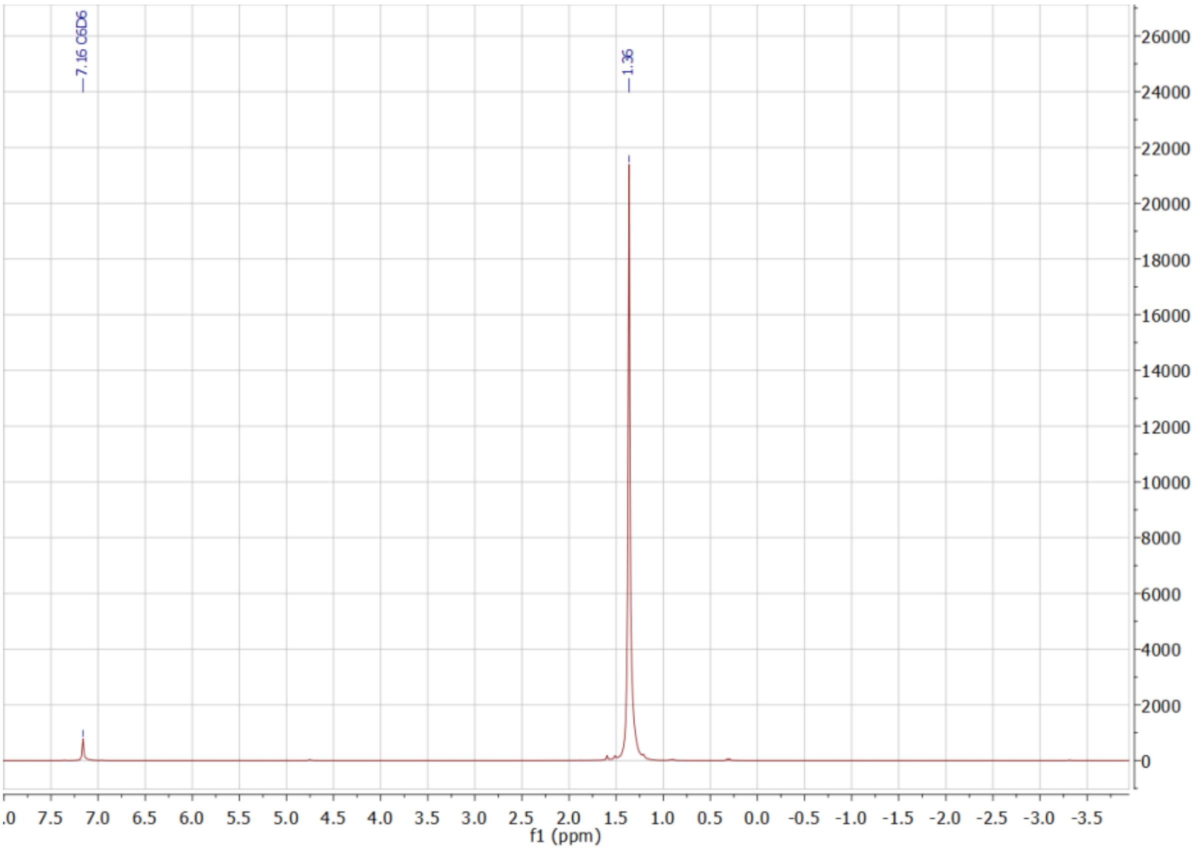

Figure S12-2: ^{1}H NMR of **$^{15}NO_3$@1** in [D6]benzene:

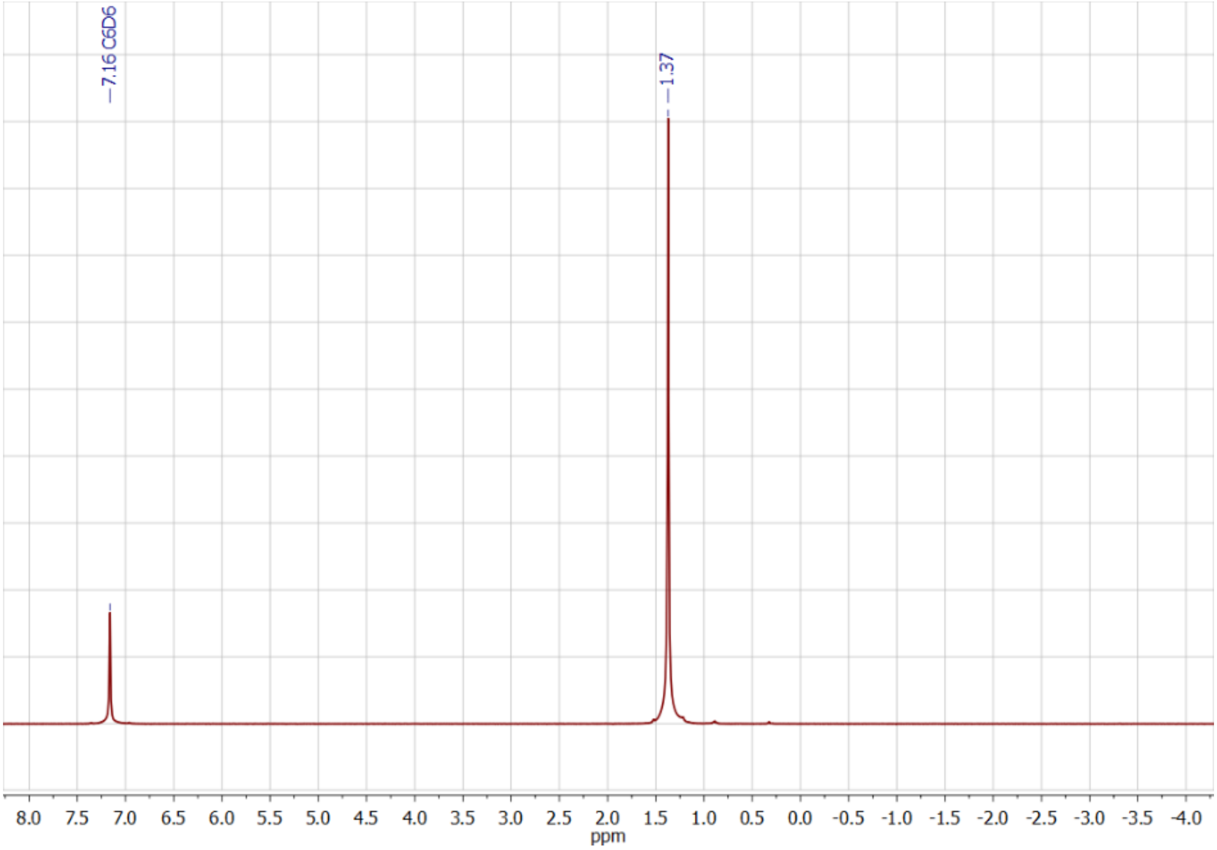

Figure S12-3: ^{15}N NMR of **$^{15}NO_3$@1** in [D6]benzene:

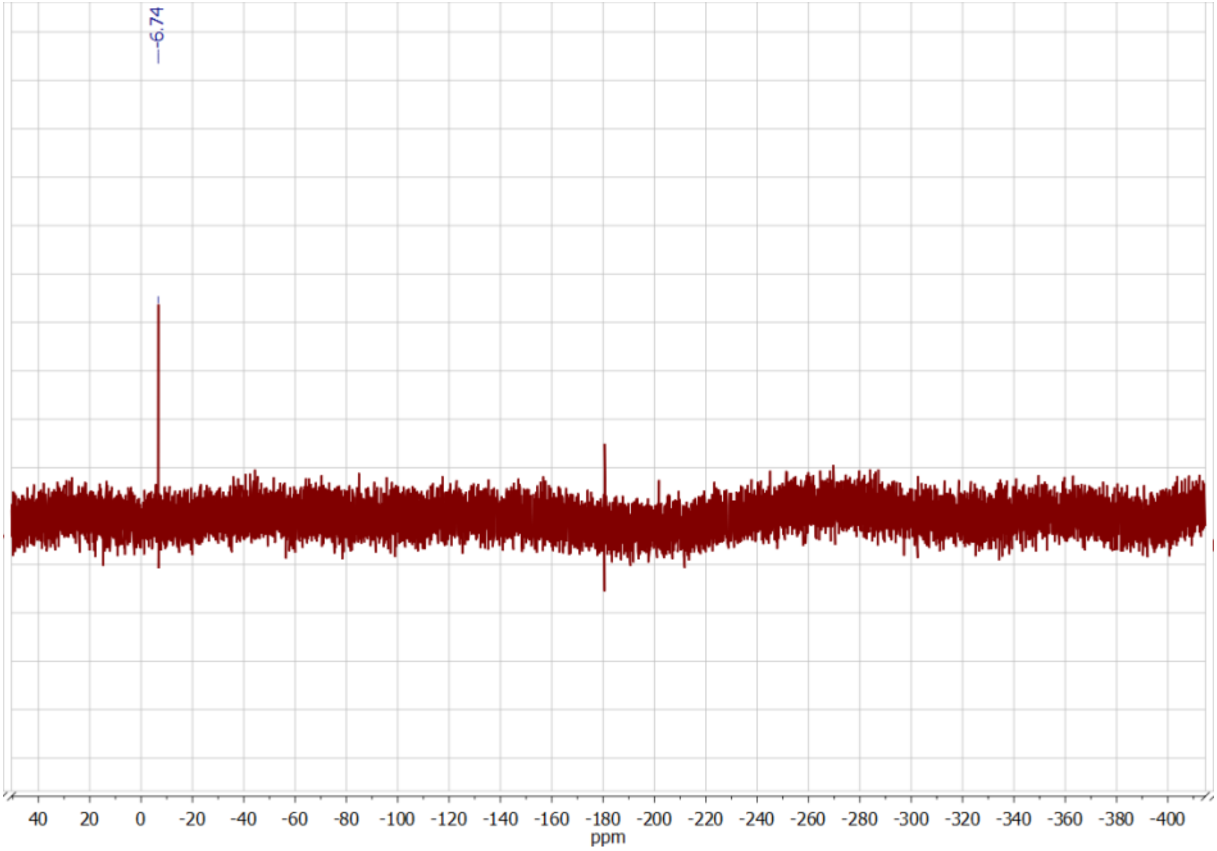

IR Spectroscopy

Figure S13-1: IR spectrum of **CH_3@1** (ATR):

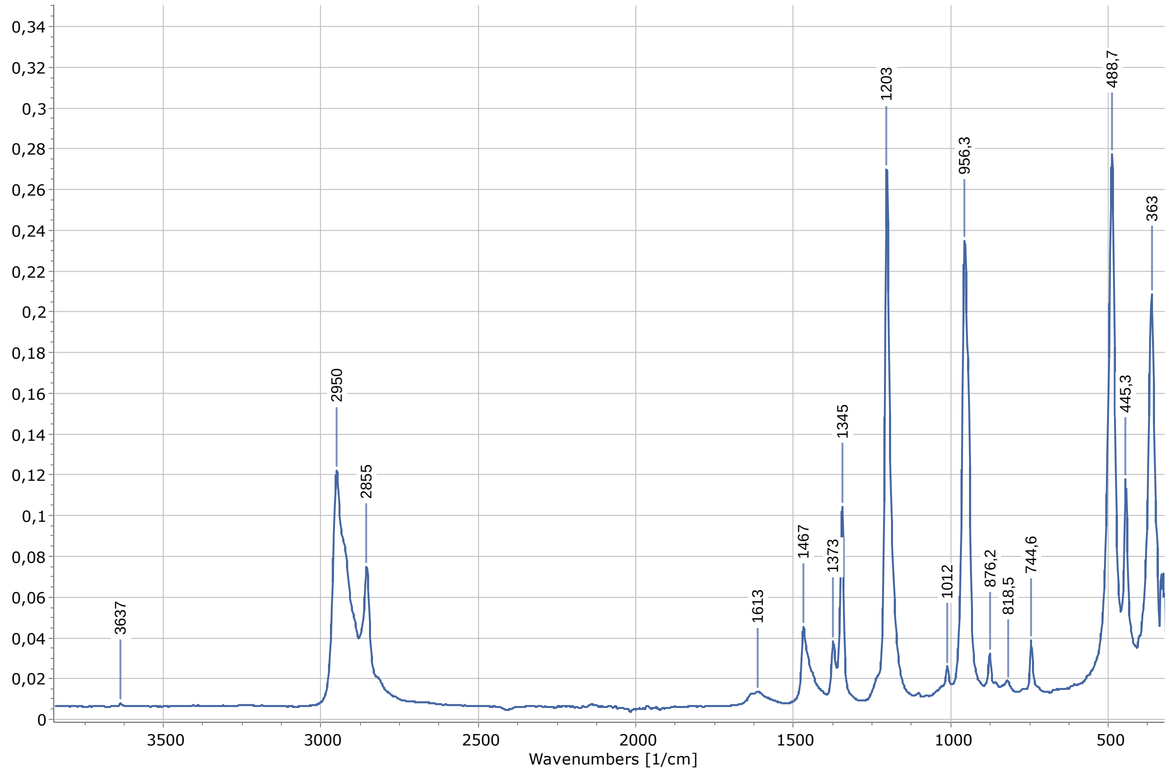

Figure S13-2: Raman spectrum of **CH_3@1** (single crystal):

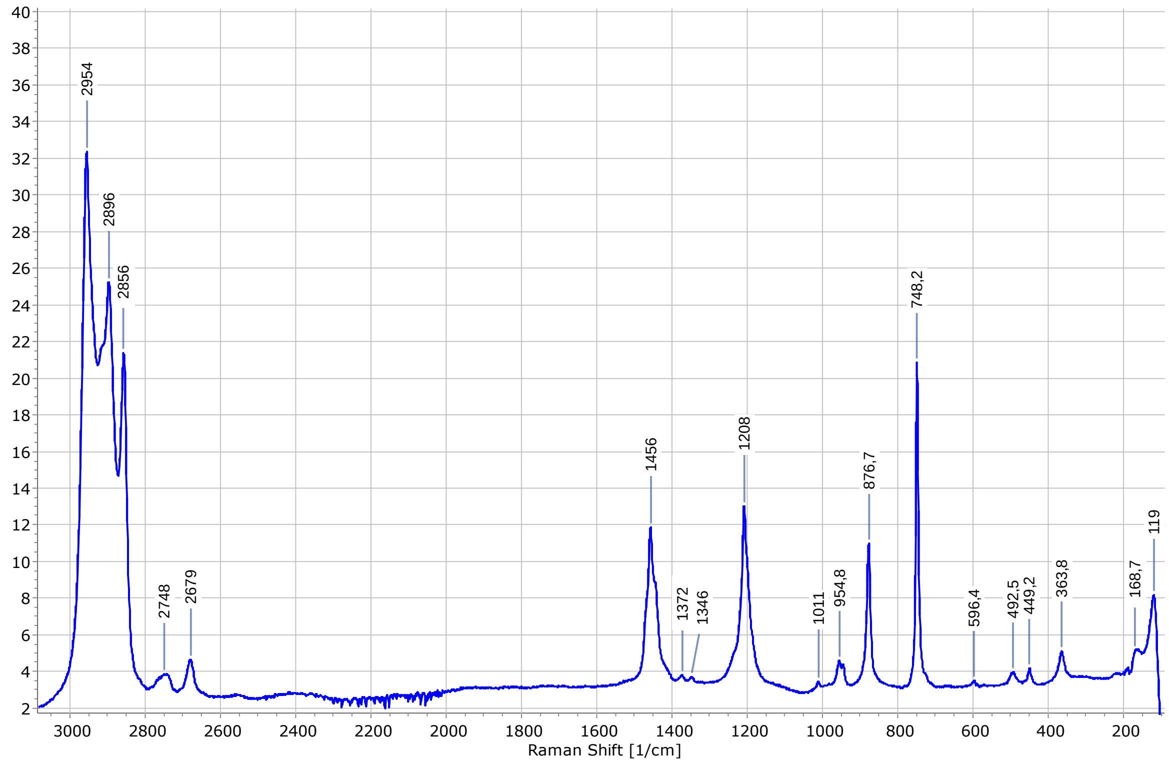

Figure S13-3: IR spectrum of **CD_3@1** (ATR):

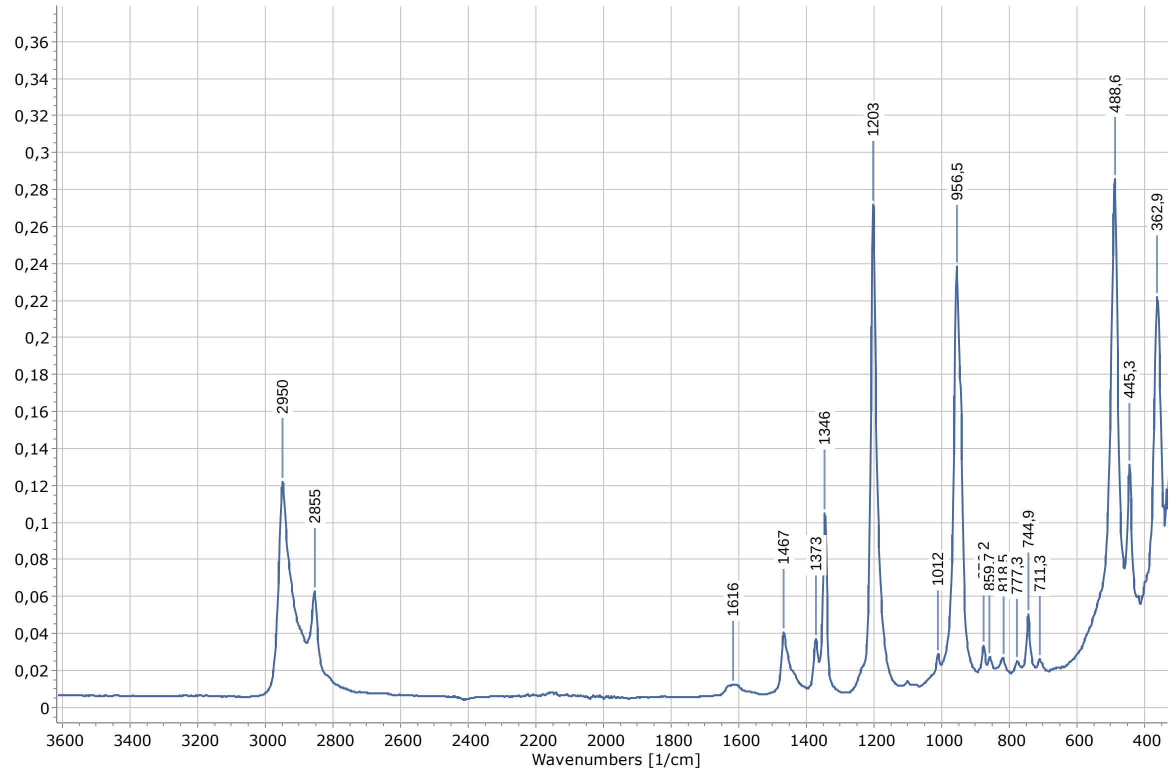

Figure S13-4: Raman spectrum of **CD_3@1** (single crystal):

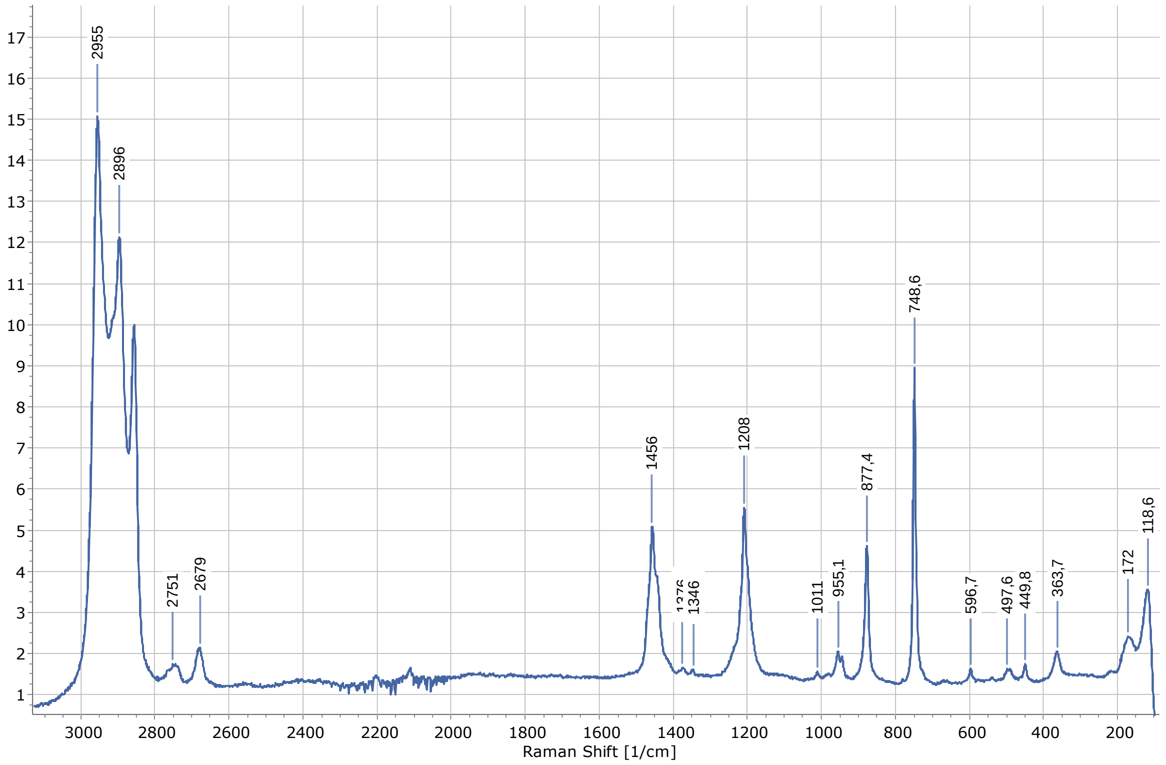

Figure S13-5: IR spectrum of NaO*t*Bu (ATR):

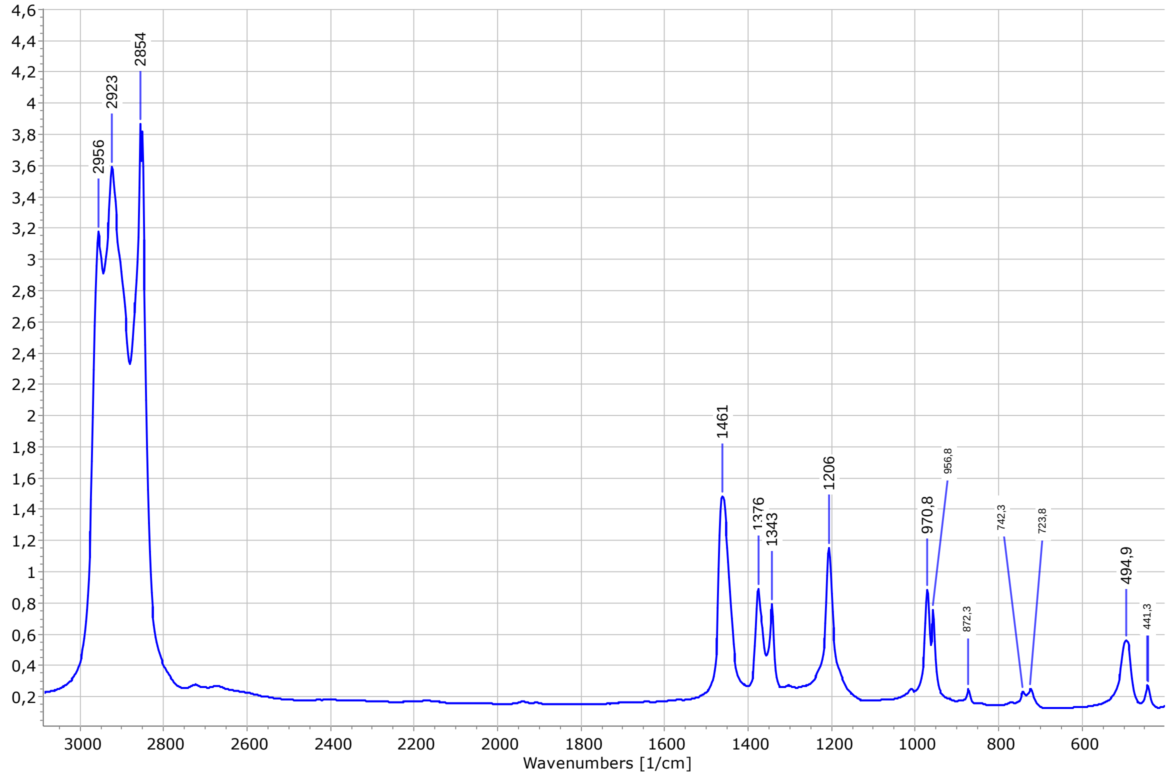

Figure S13-6: Raman spectrum of NaO*t*Bu (single crystal):

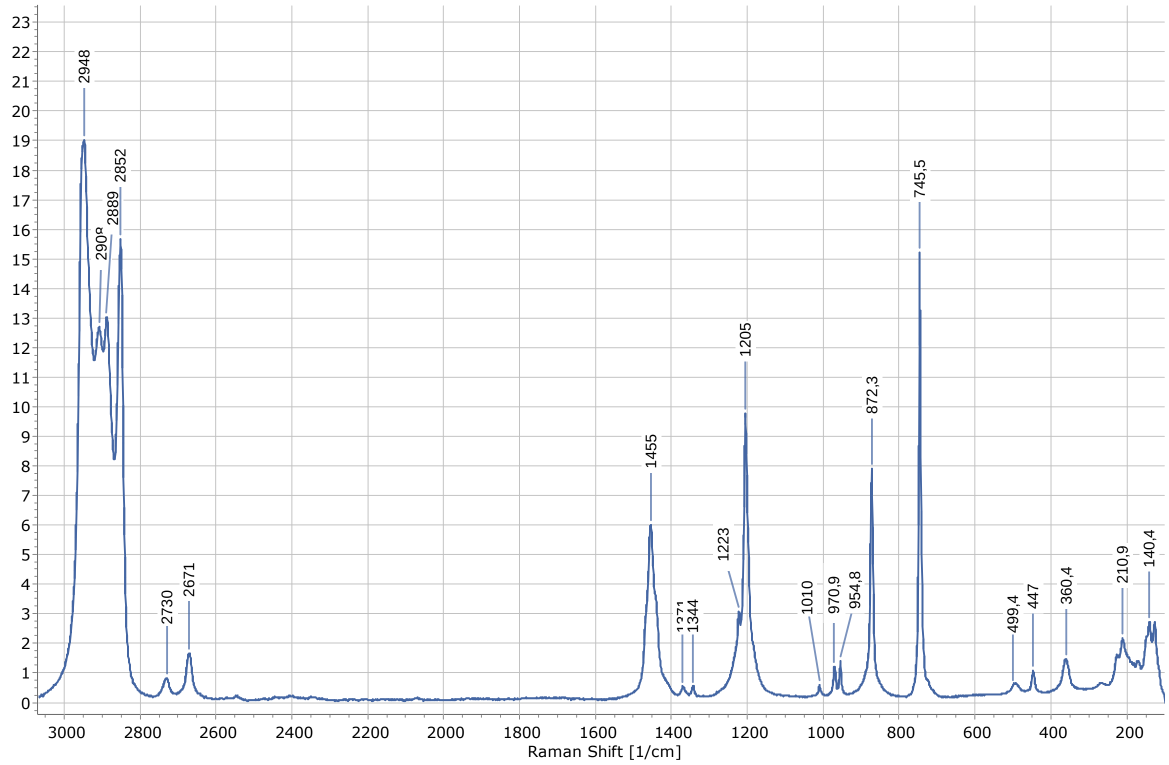

Figure S13-7: Compared IR spectra of NaO*t*Bu and **CH_3@1** (ATR):

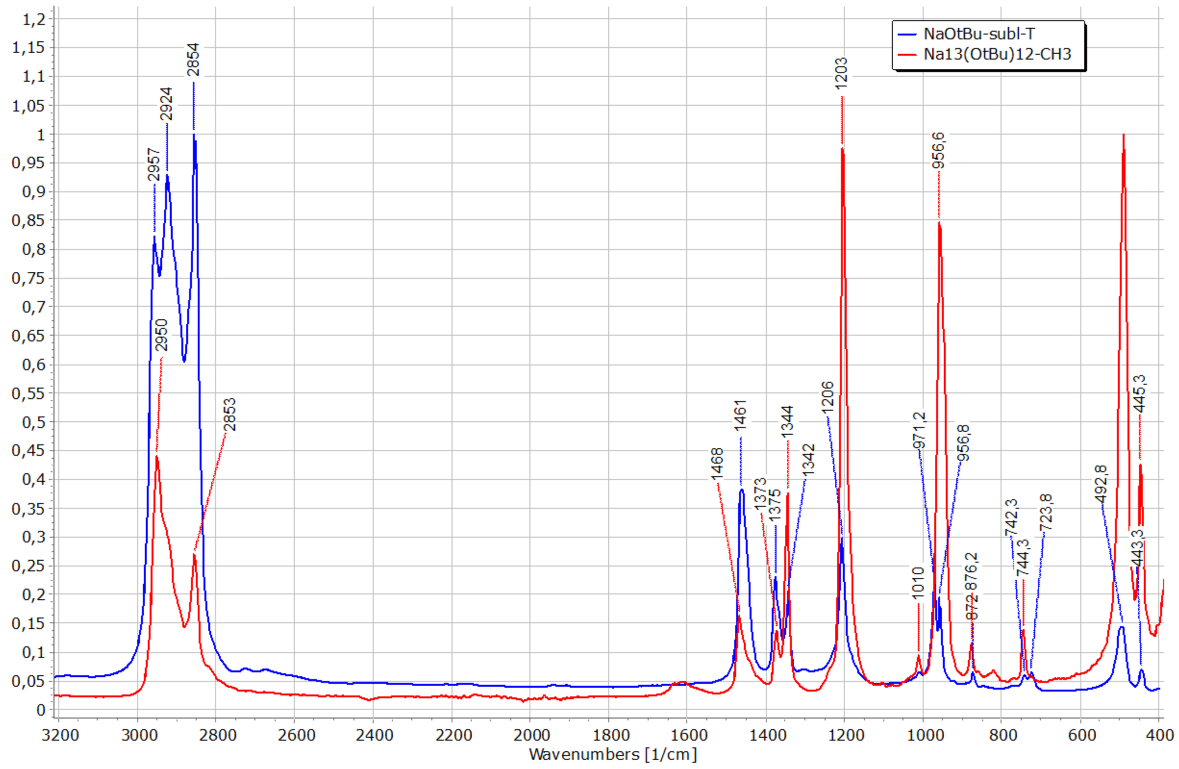

Figure S13-8: Compared Raman spectra of NaO*t*Bu (blue) and **CH_3@1** (red):

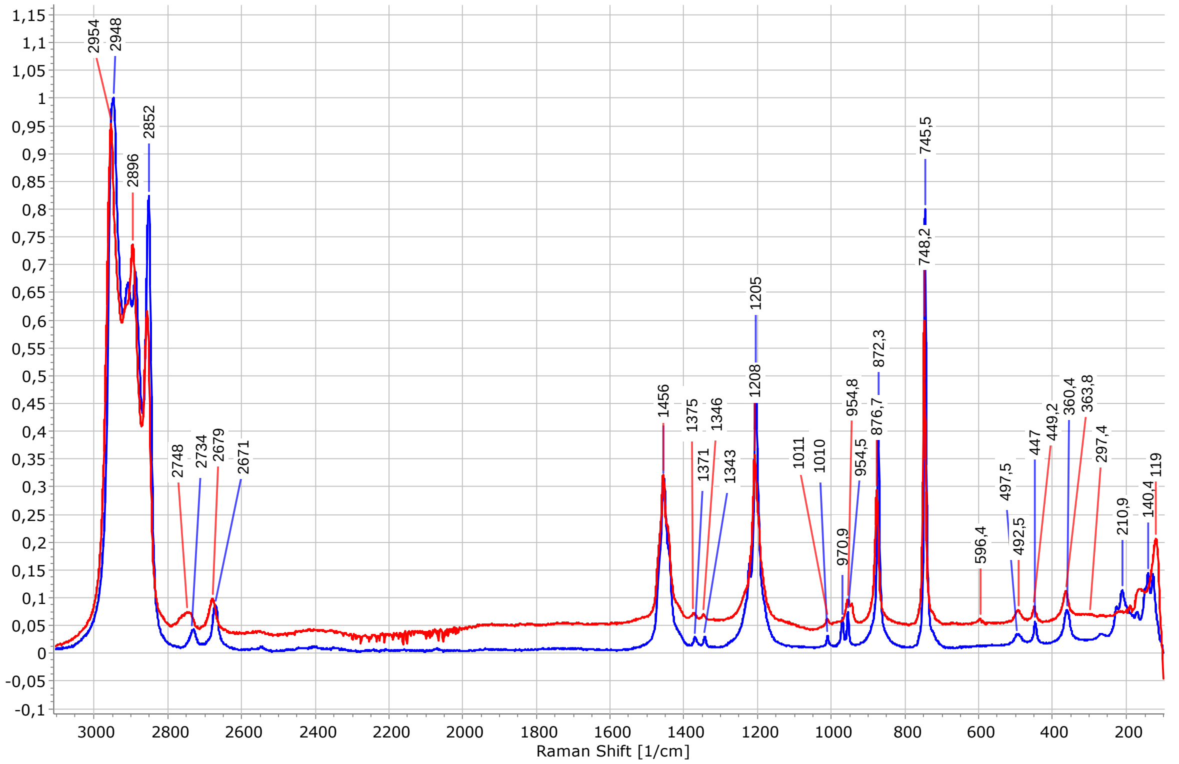

Figure S14-1: IR spectrum of **CN@1** (ATR):

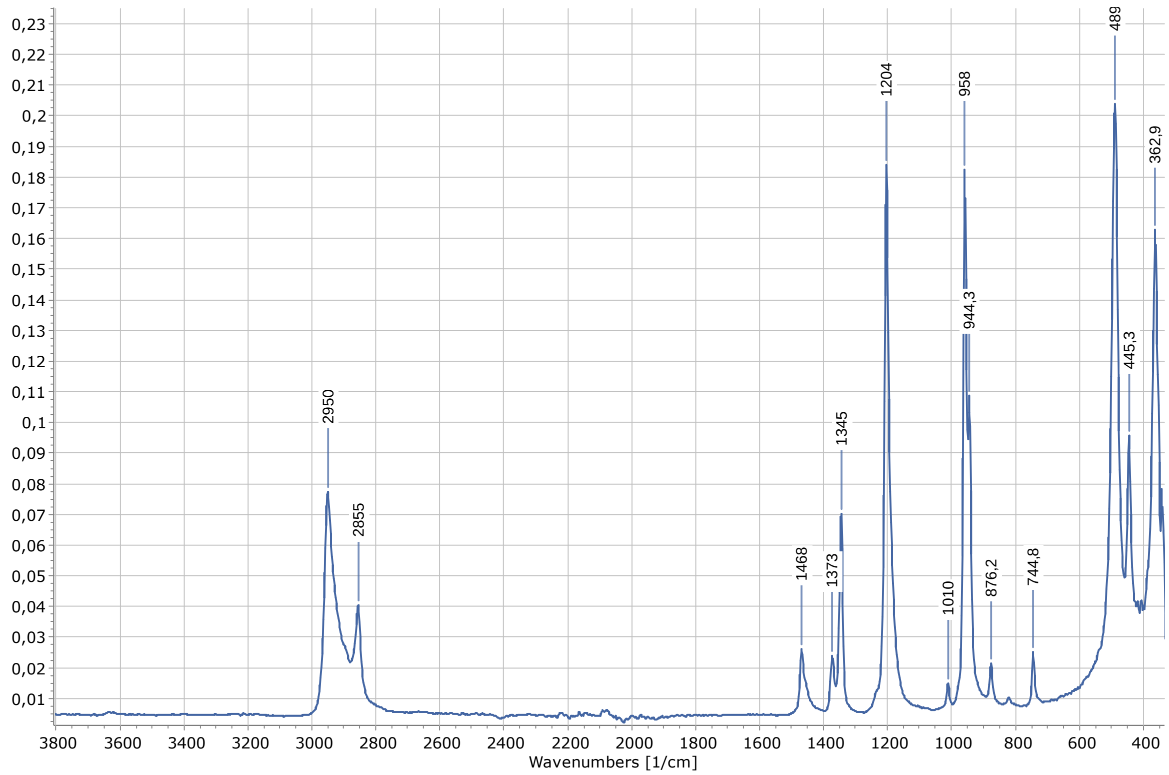

Figure S14-2: Raman spectrum of **CN@1** (single crystal):

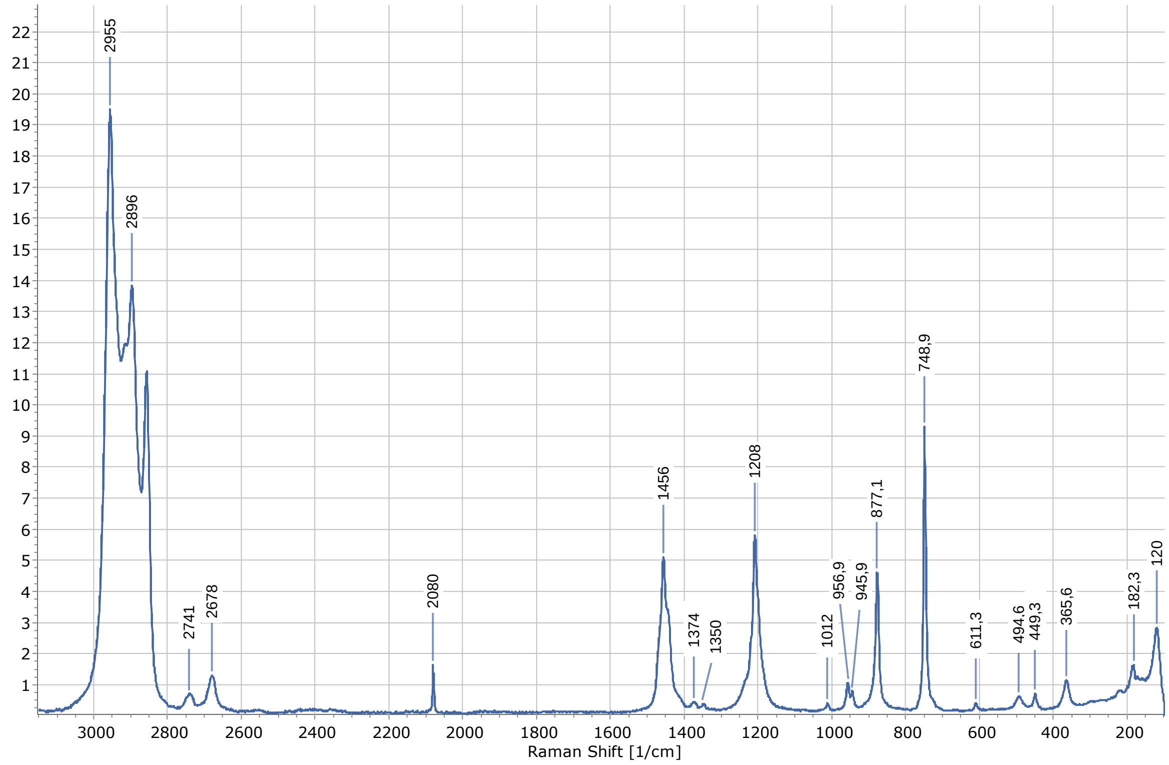

Figure S14-3: IR spectrum of ^{13}**CN@1** (ATR):

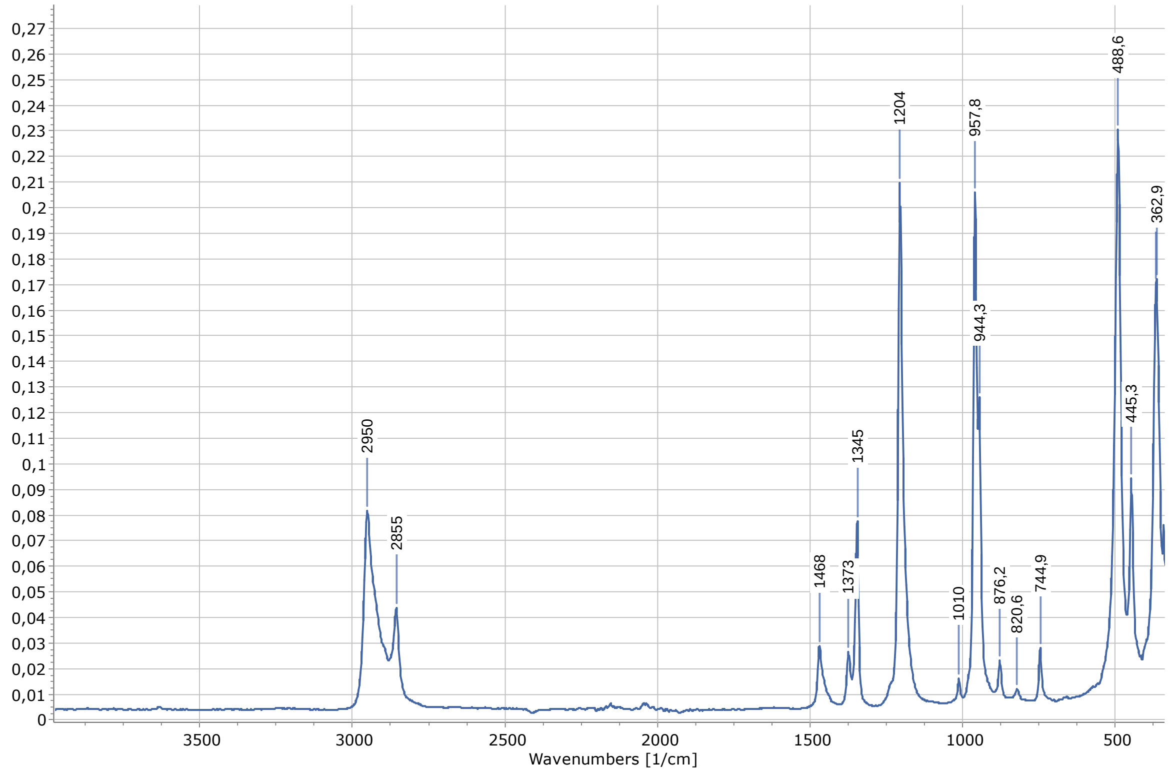

Figure S14-4: Raman spectrum of ^{13}**CN@1** (single crystal):

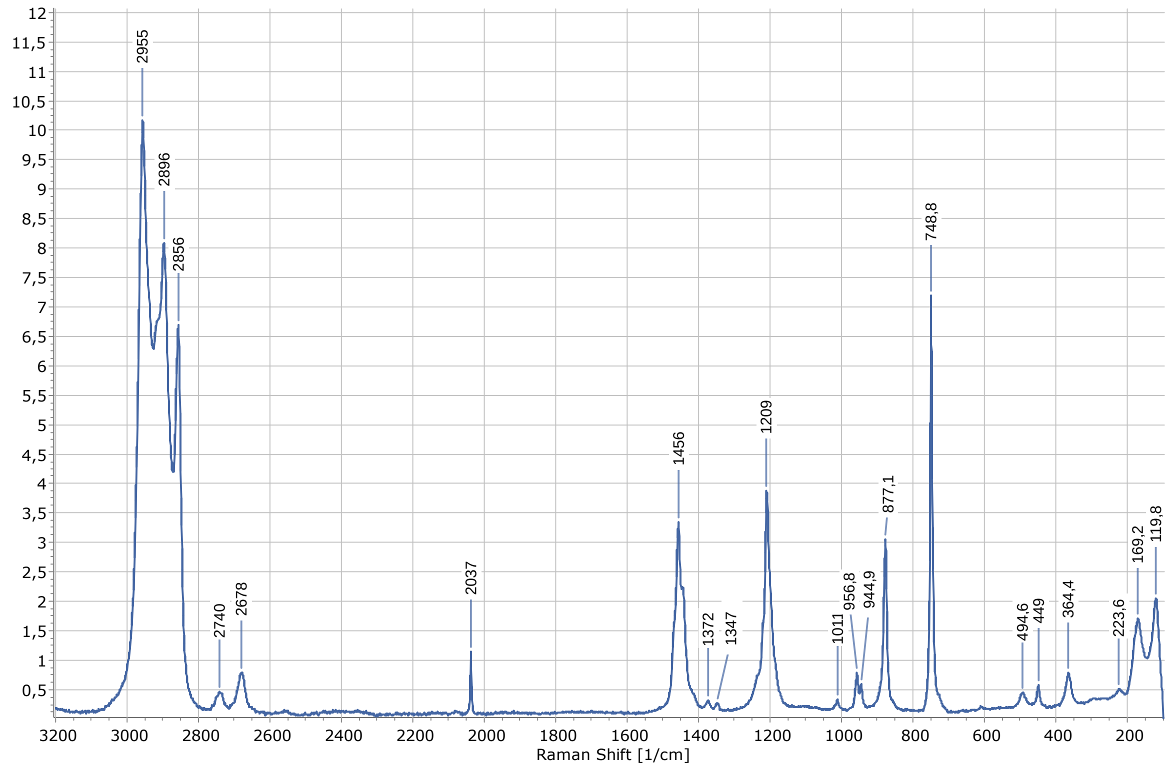

Figure S14-5: IR spectrum (detail) of **CN@1** (blue) and **^{13}CN@1** (green)

(nujol mull, CsBr plates):

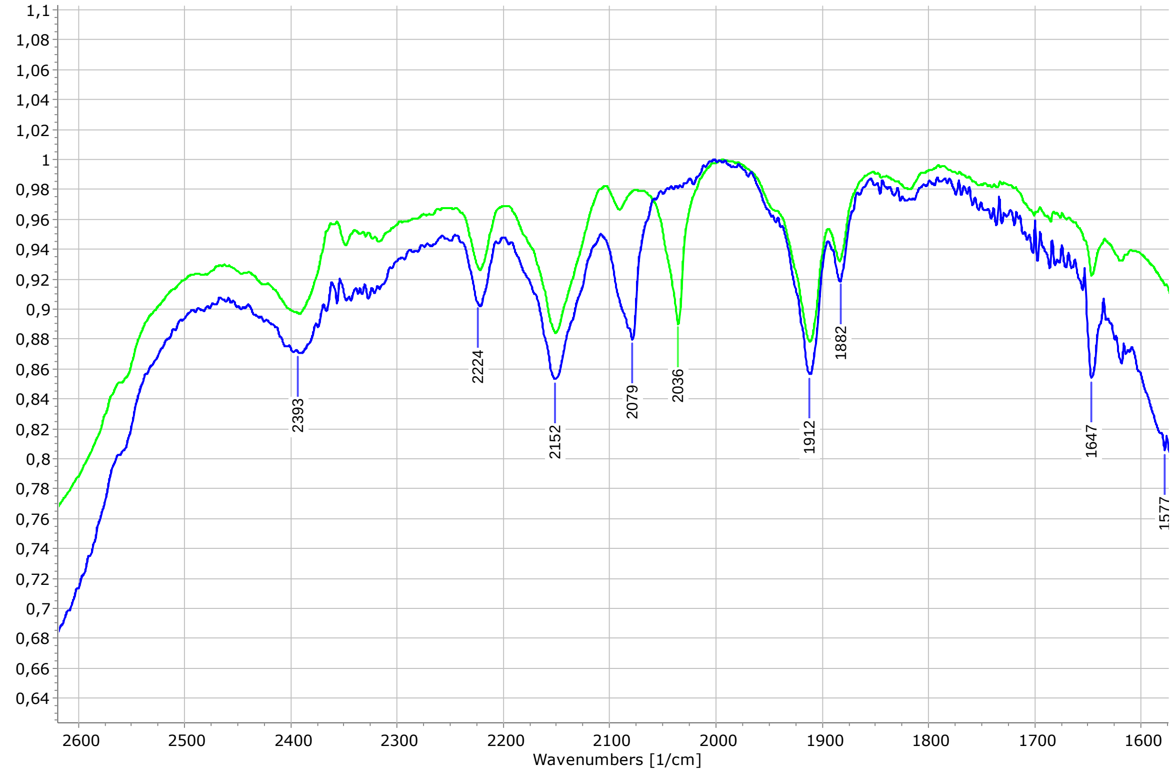

Figure S15-1: IR spectrum of **F-2** (ATR):

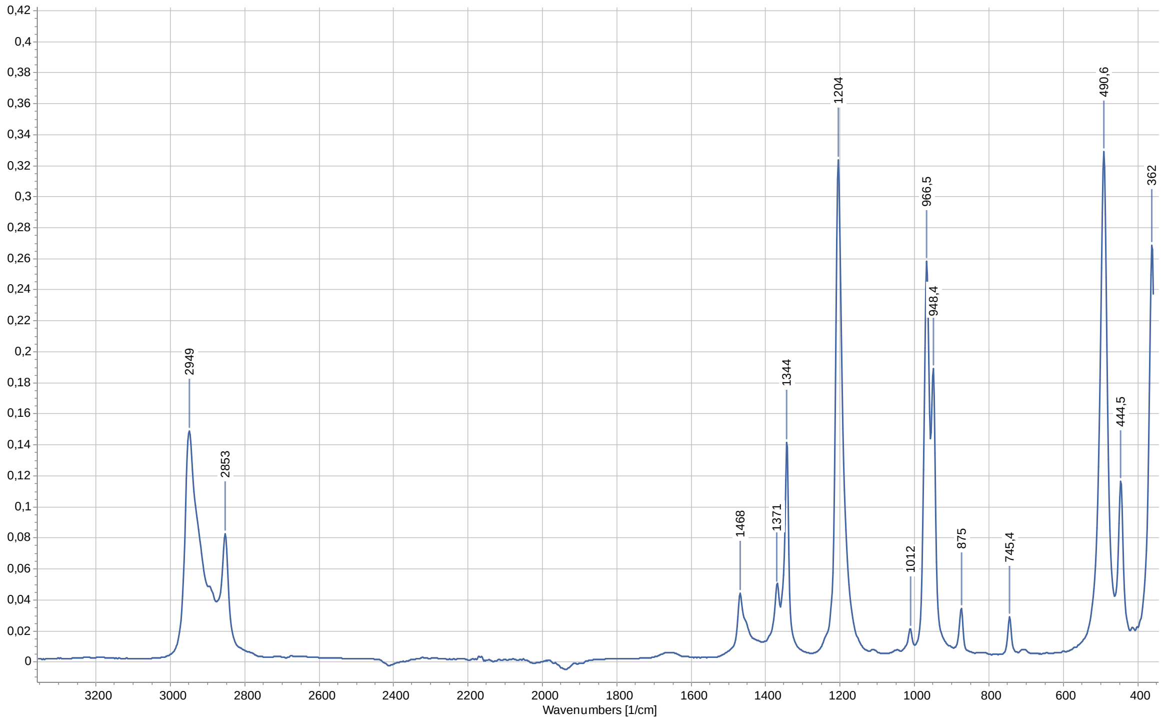

Figure S15-2: Raman spectrum of **F-2** (single crystal):

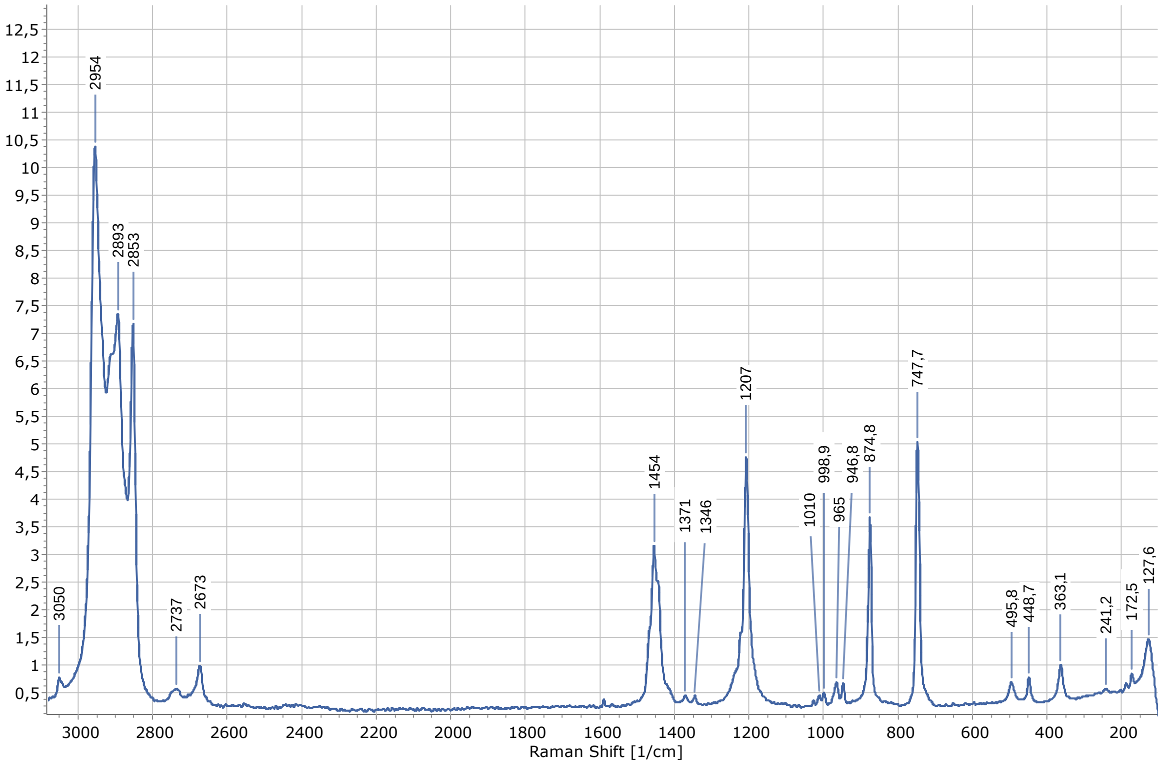

Figure S16-1: IR spectrum of **Cl@1** (ATR):

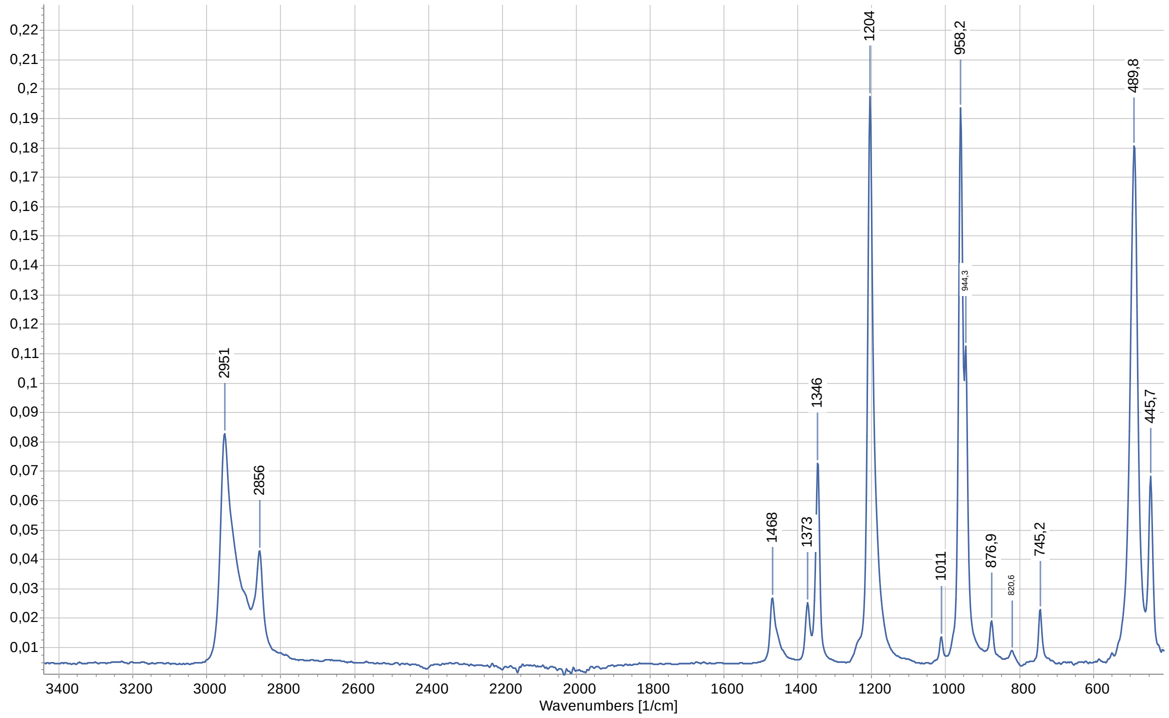

Figure S16-2: Raman spectrum of **Cl@1** (single crystal):

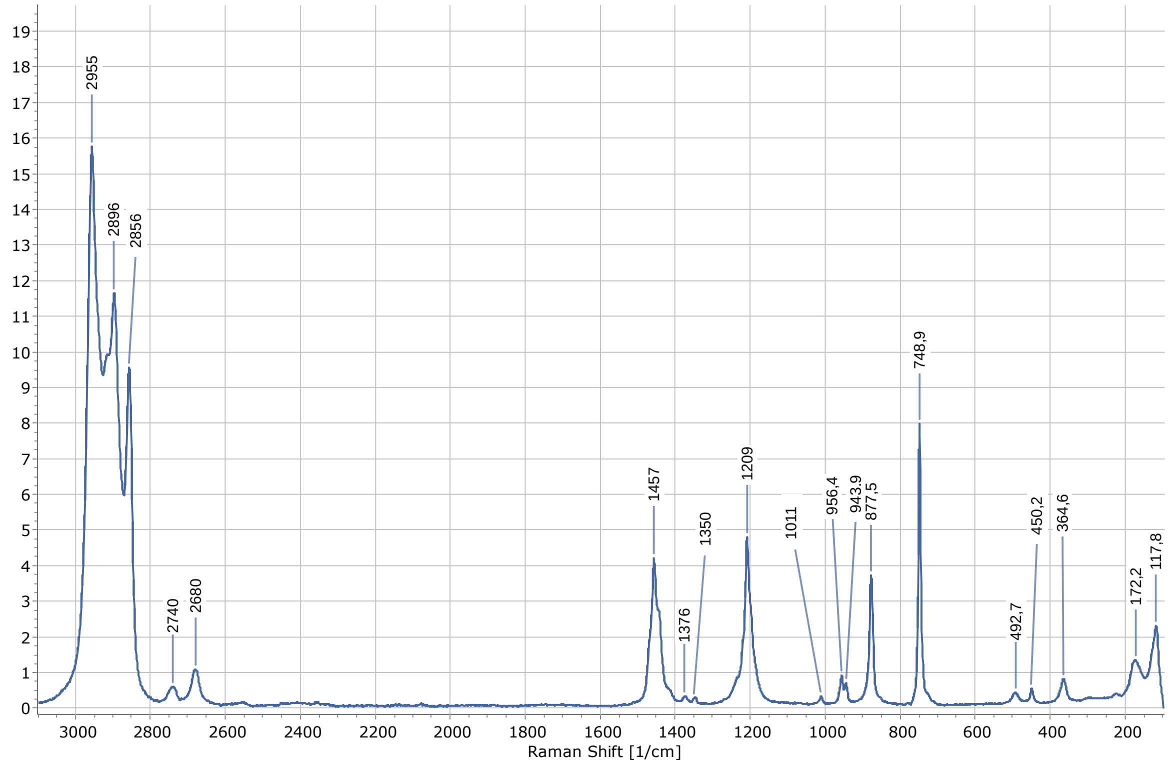

Figure S17-1: IR spectrum of **Br@1** (ATR):

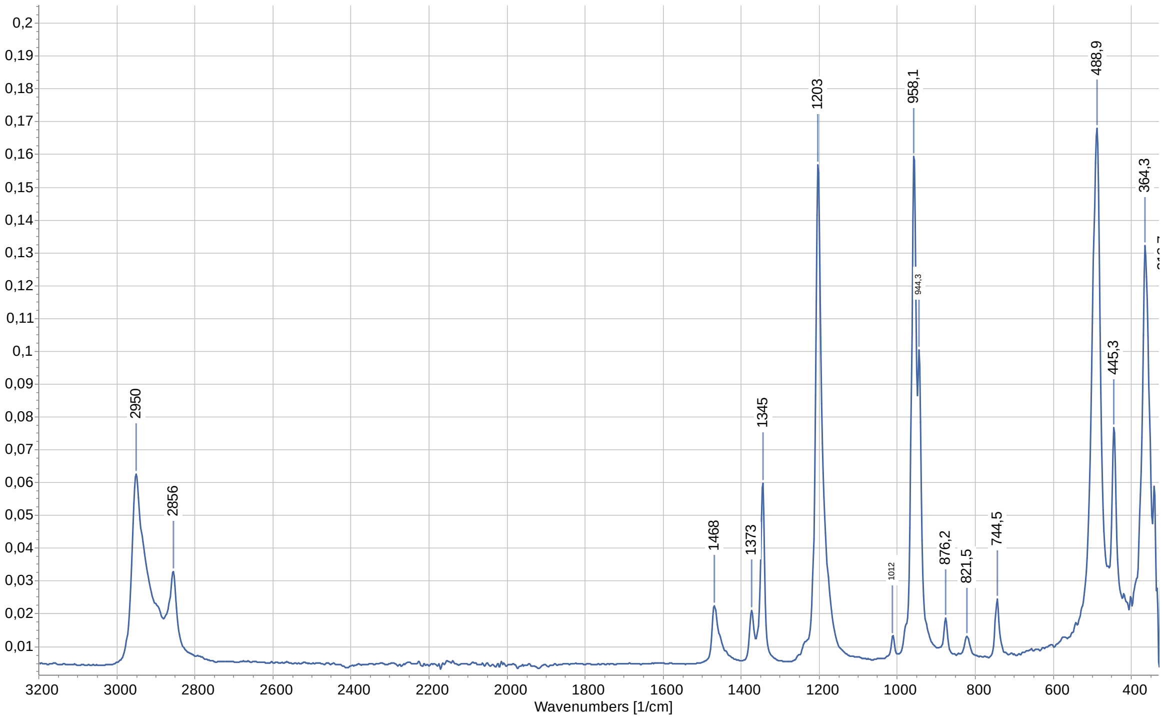

Figure S17-2: Raman spectrum of **Br@1** (single crystal):

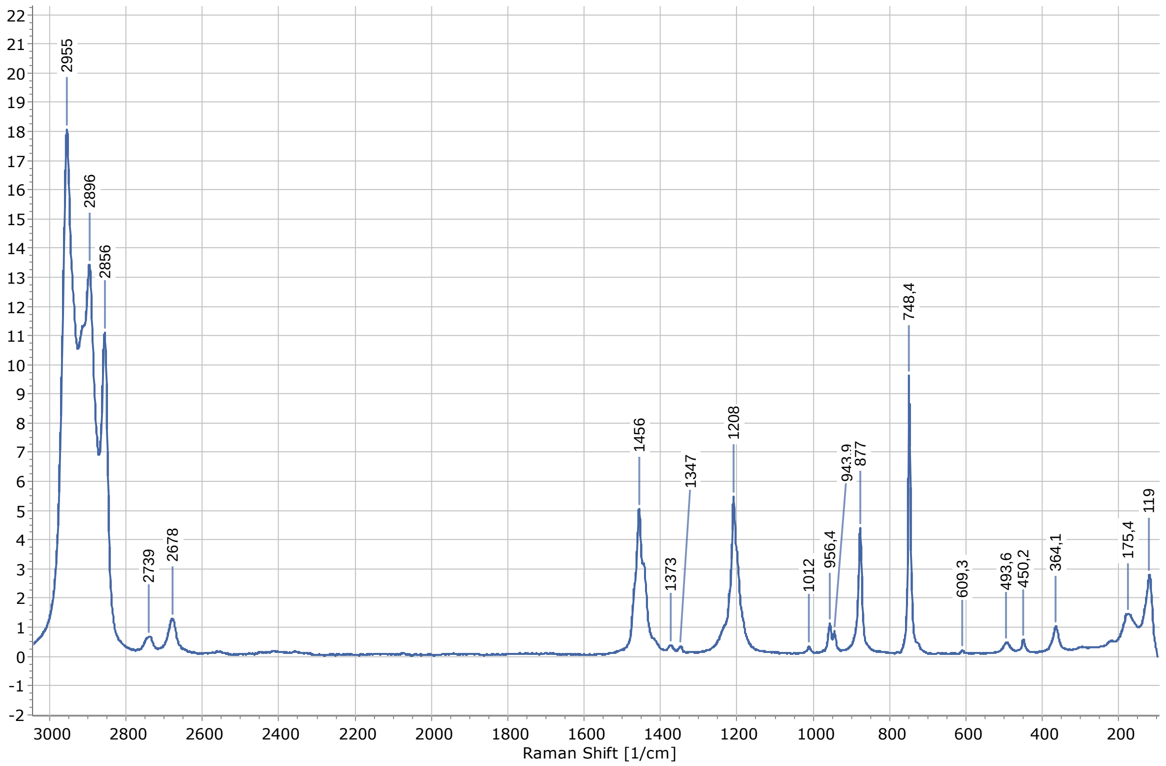

Figure S18-1: IR spectrum of **I@1** (ATR):

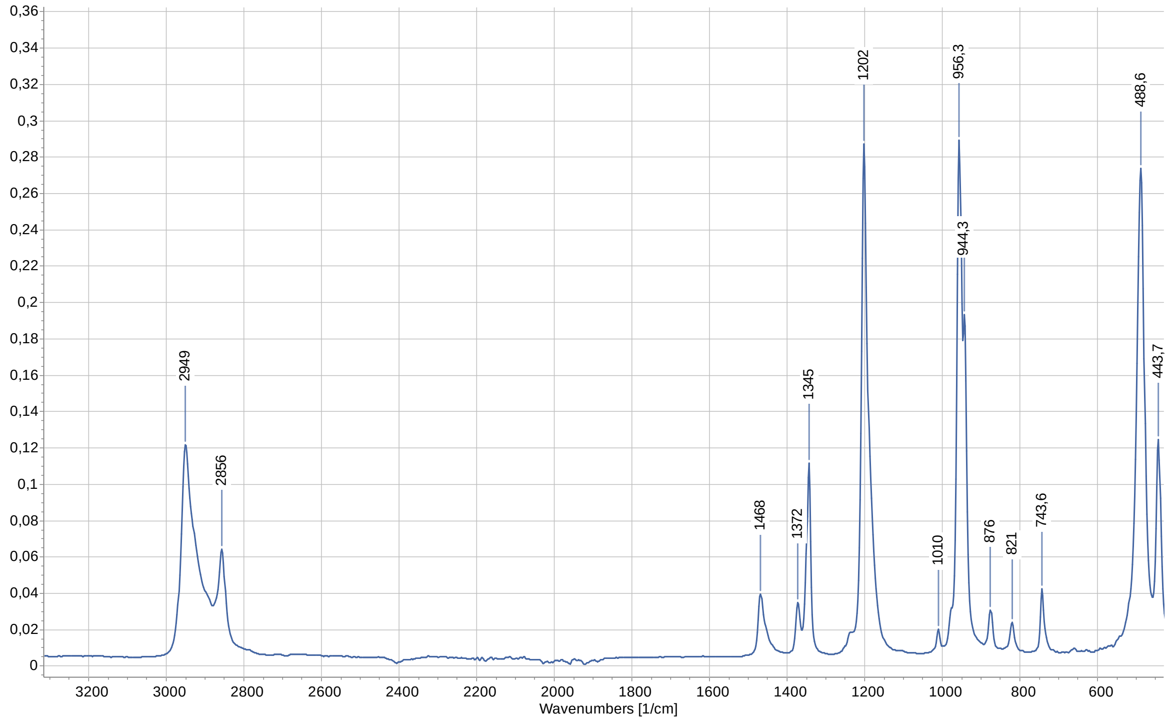

Figure S18-2: Raman spectrum of **I@1** (single crystal):

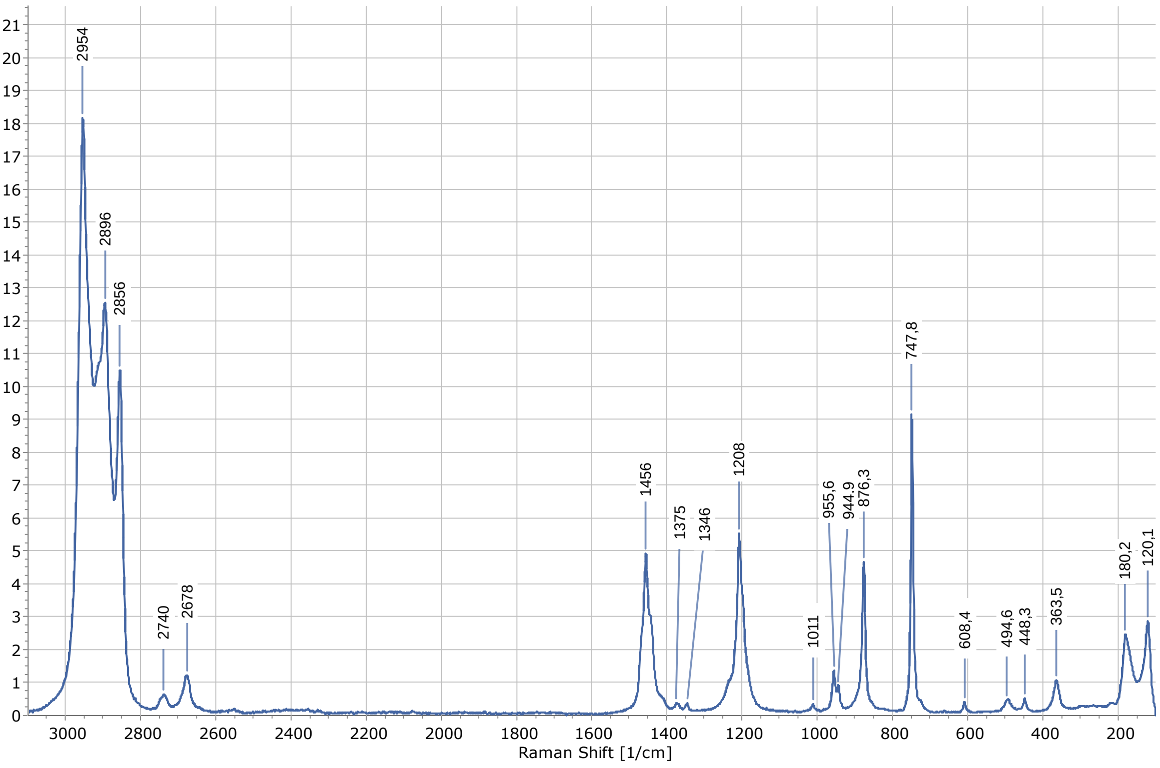

Figure S19-1: IR spectrum of **OCN@1** (ATR):

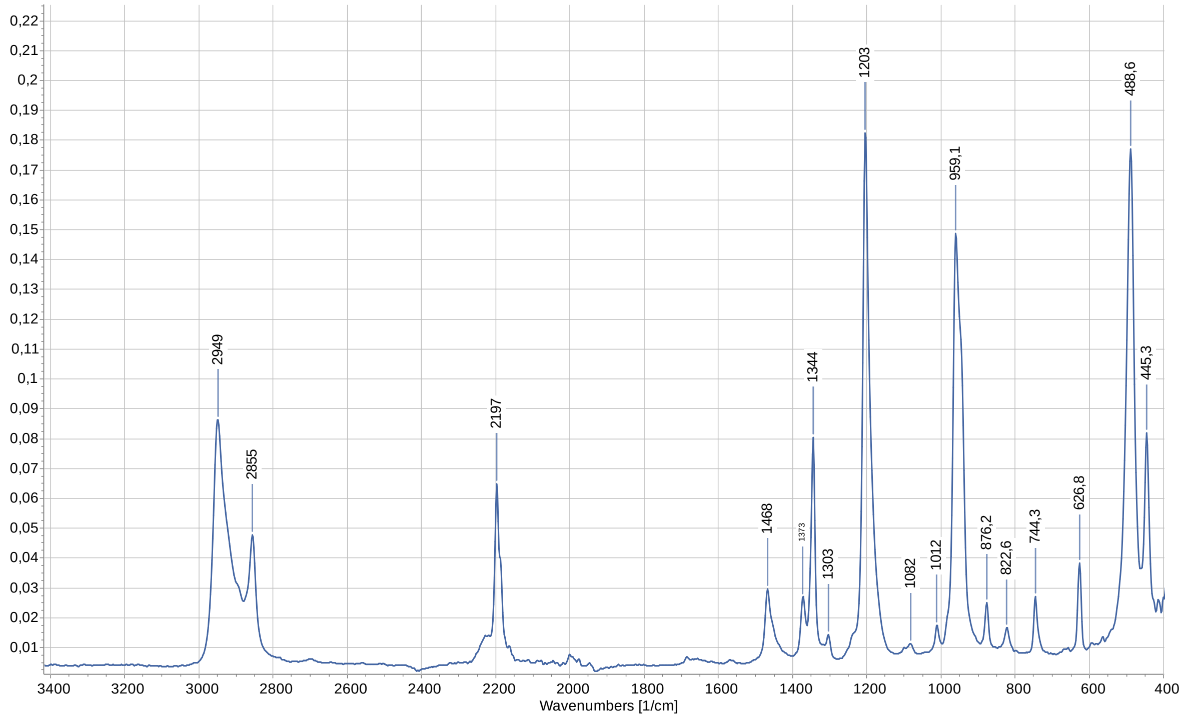

Figure S19-2: Raman spectrum of **OCN@1** (single crystal):

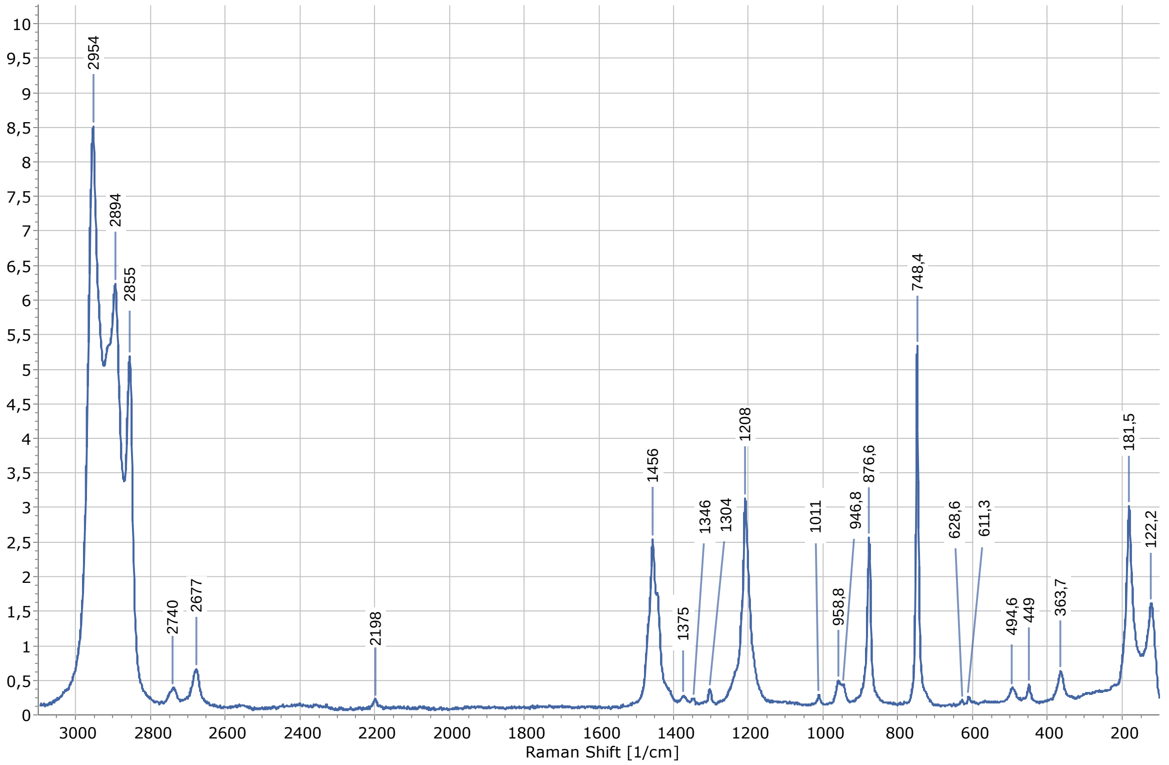

Figure S20-1: IR spectrum of **SCN@1** (ATR):

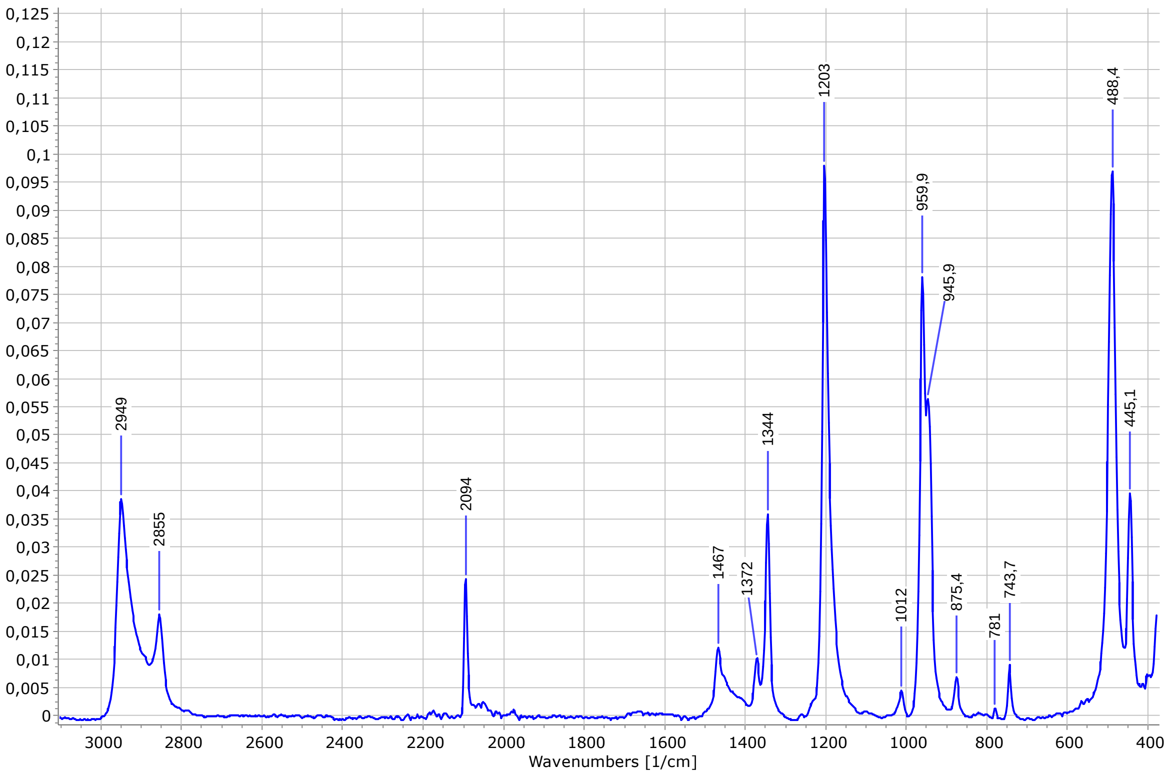

Figure S20-2: Raman spectrum of **SCN@1** (single crystal):

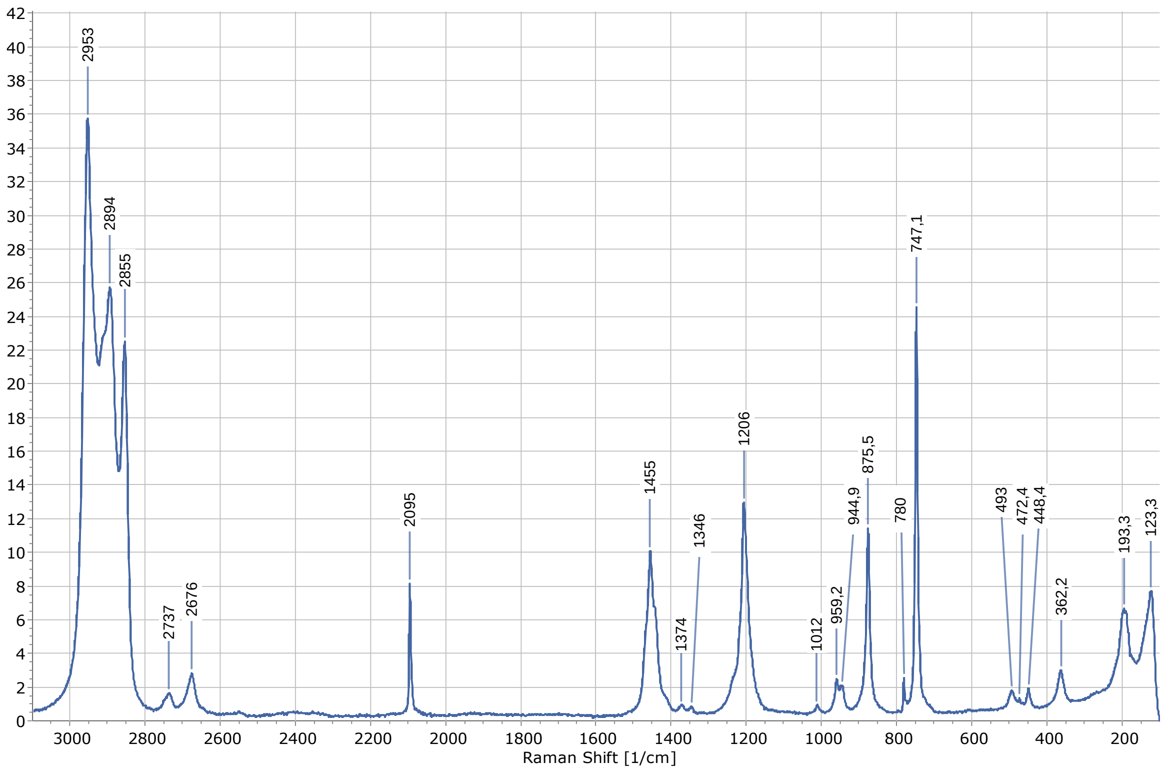

Figure S21-1: IR spectrum of **N_3@1** (ATR):

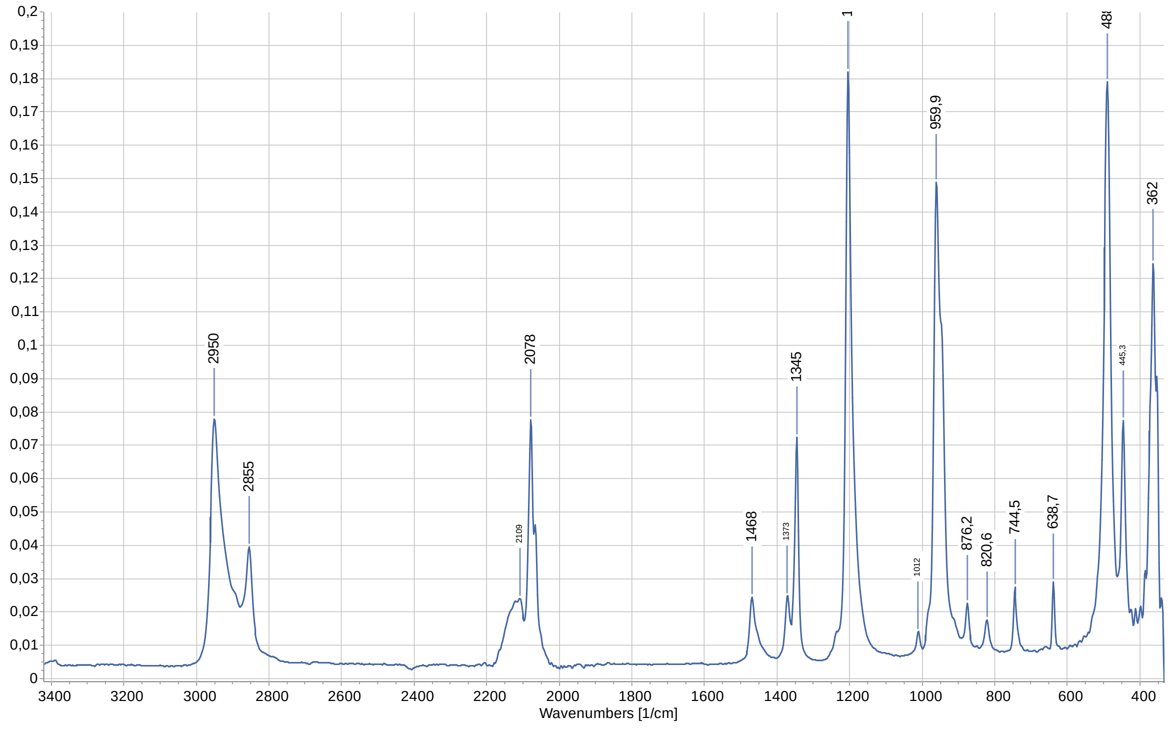

Figure S21-2: Raman spectrum of **N_3@1** (single crystal):

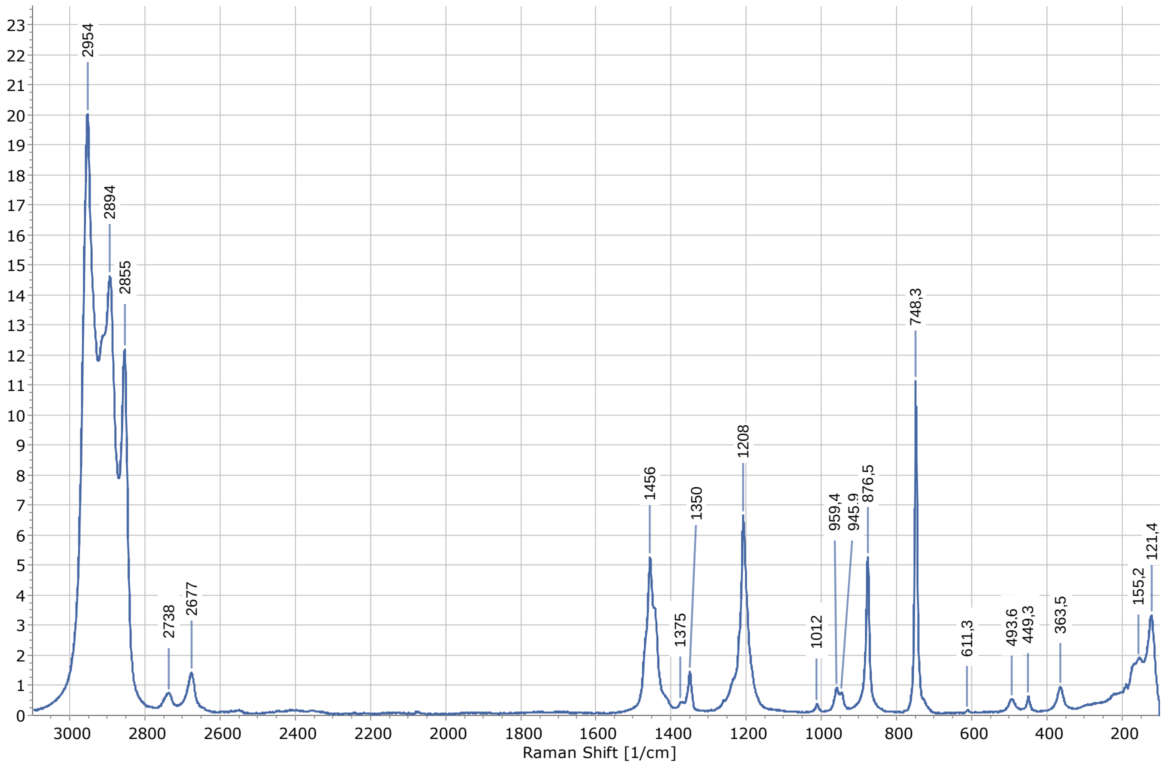

Figure S22-1: IR spectrum of **1-$^{15}N_3$@1** (ATR):

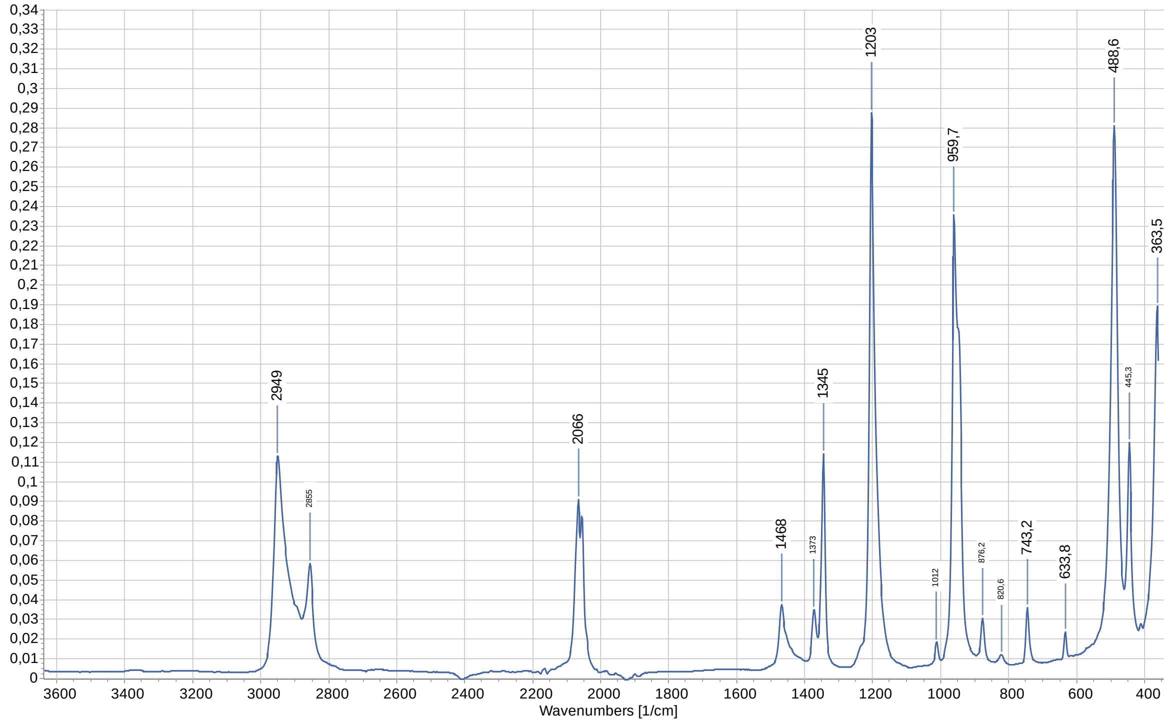

Figure S22-2: Raman spectrum of **1-$^{15}N_3$@1** (single crystal):

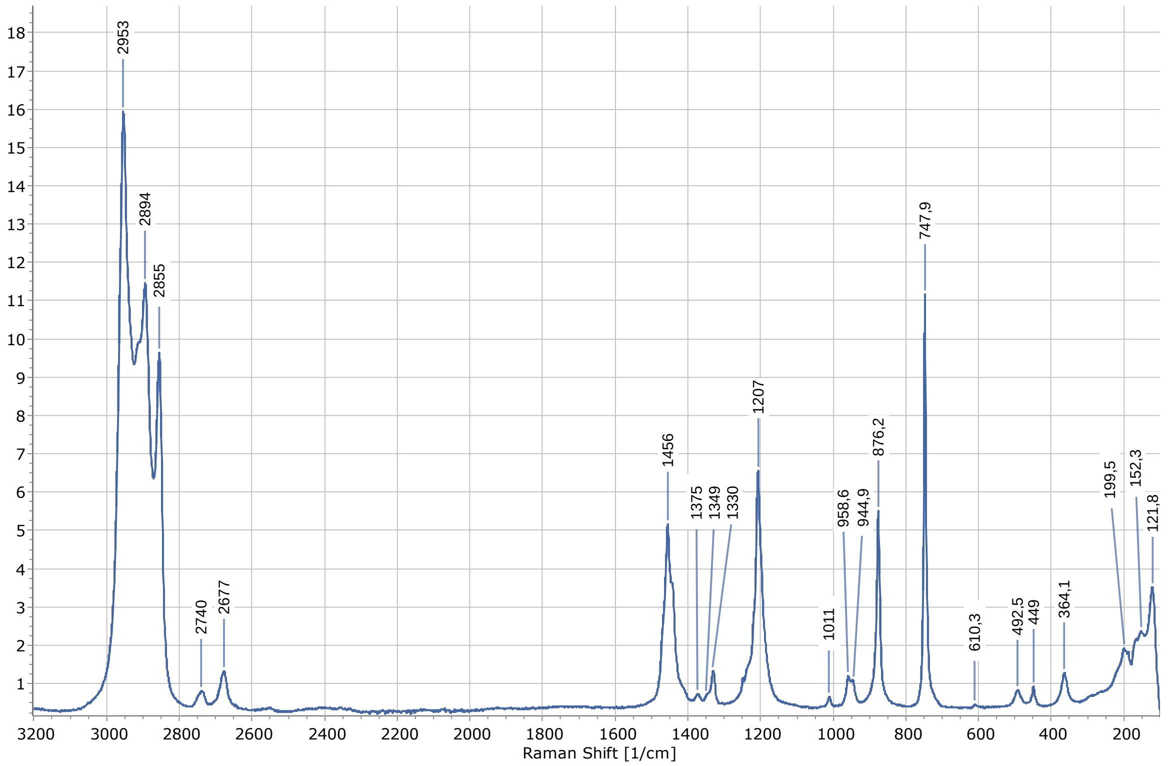

Figure S23-1: IR spectrum of **NO_3@1** (ATR):

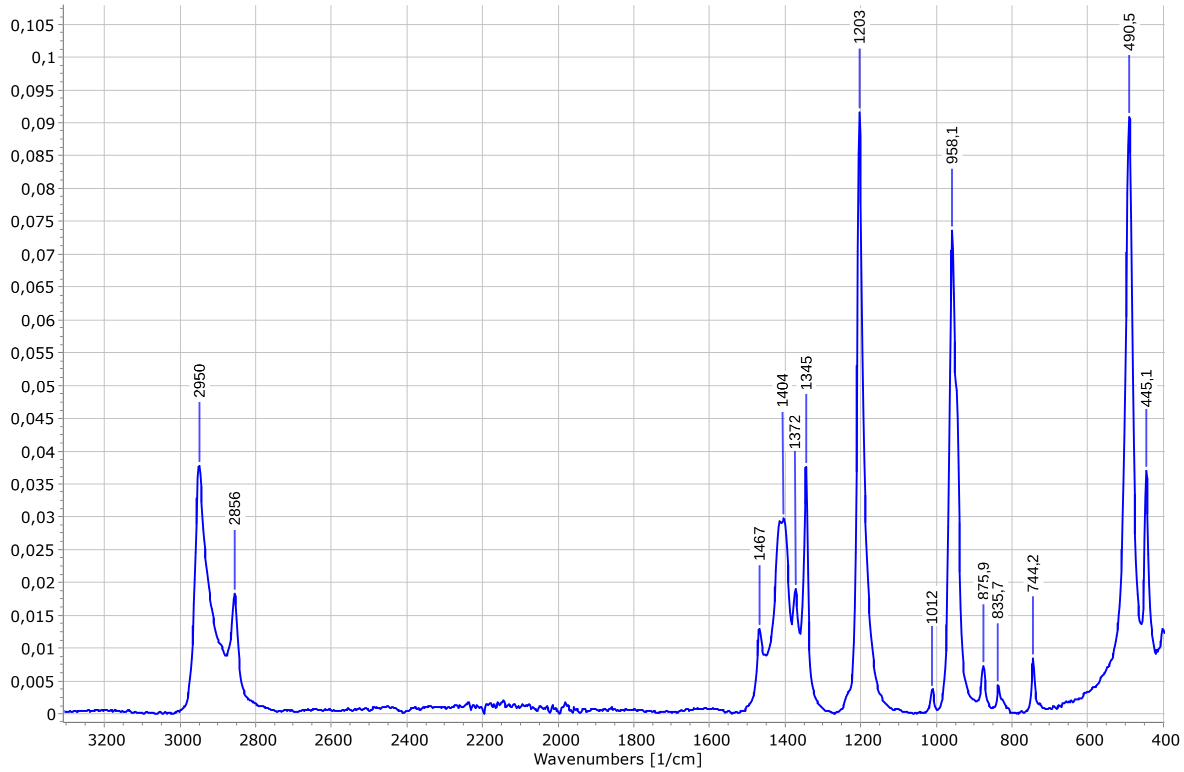

Figure S23-2: Raman spectrum of **NO_3@1** (single crystal):

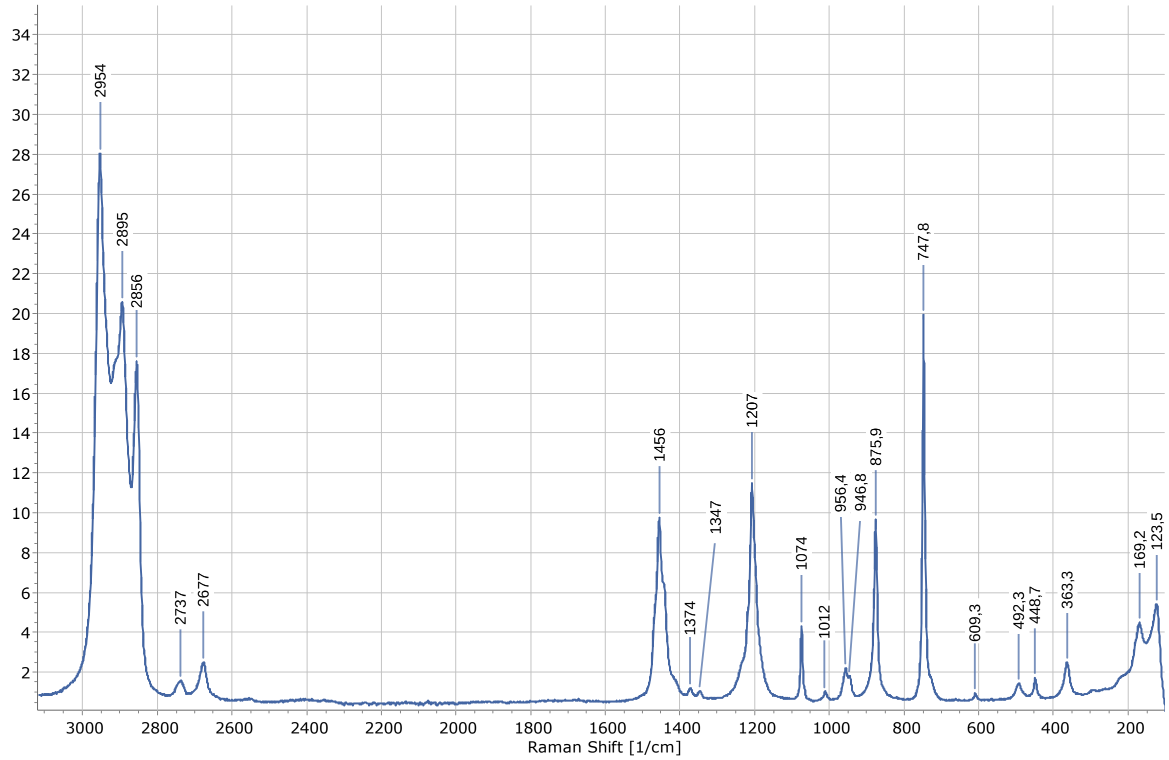

Figure S24-1: IR spectrum of ^{15}**NO$_3$@1** (ATR):

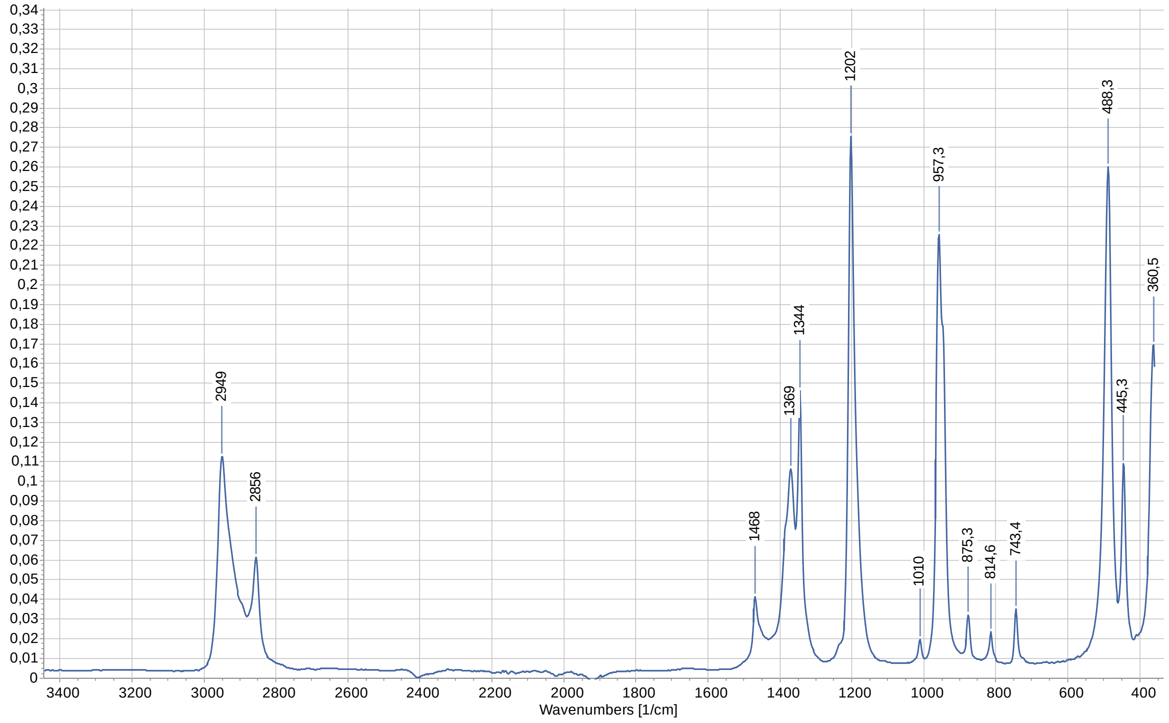

Figure S24-2: Raman spectrum of ^{15}**NO$_3$@1** (single crystal):

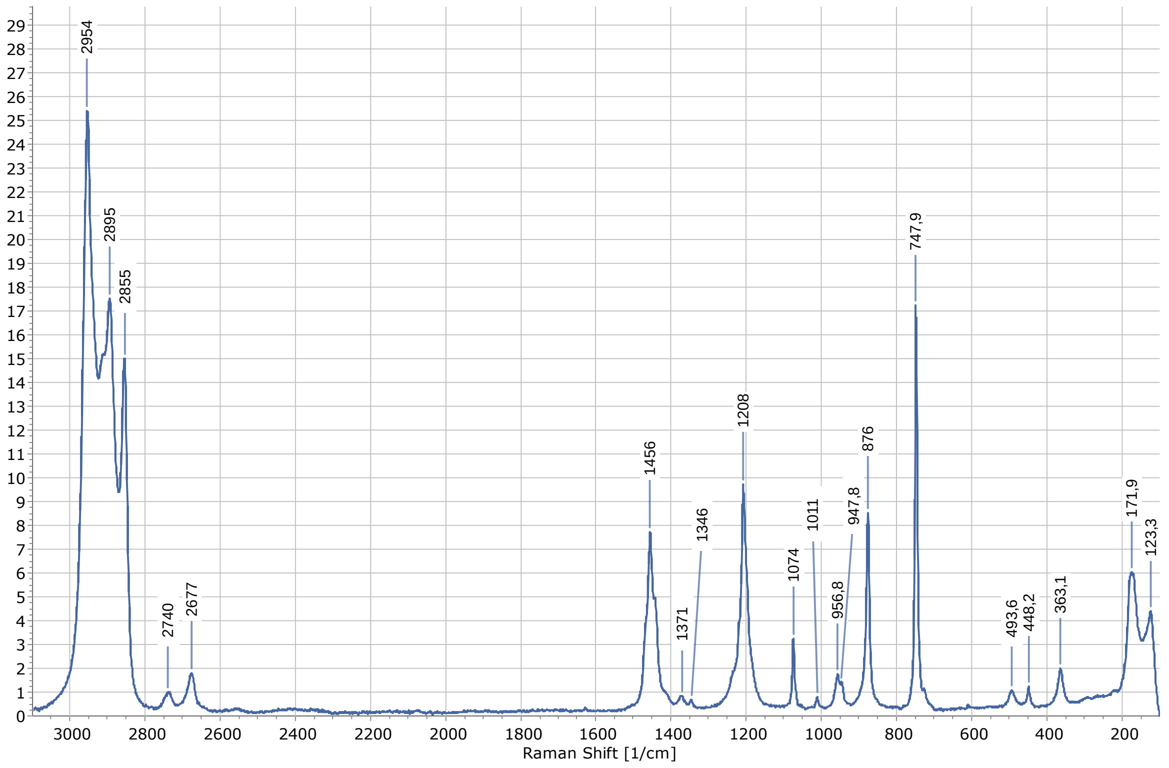

Figure S25-1: Raman spectrum of reference compound NaCN:

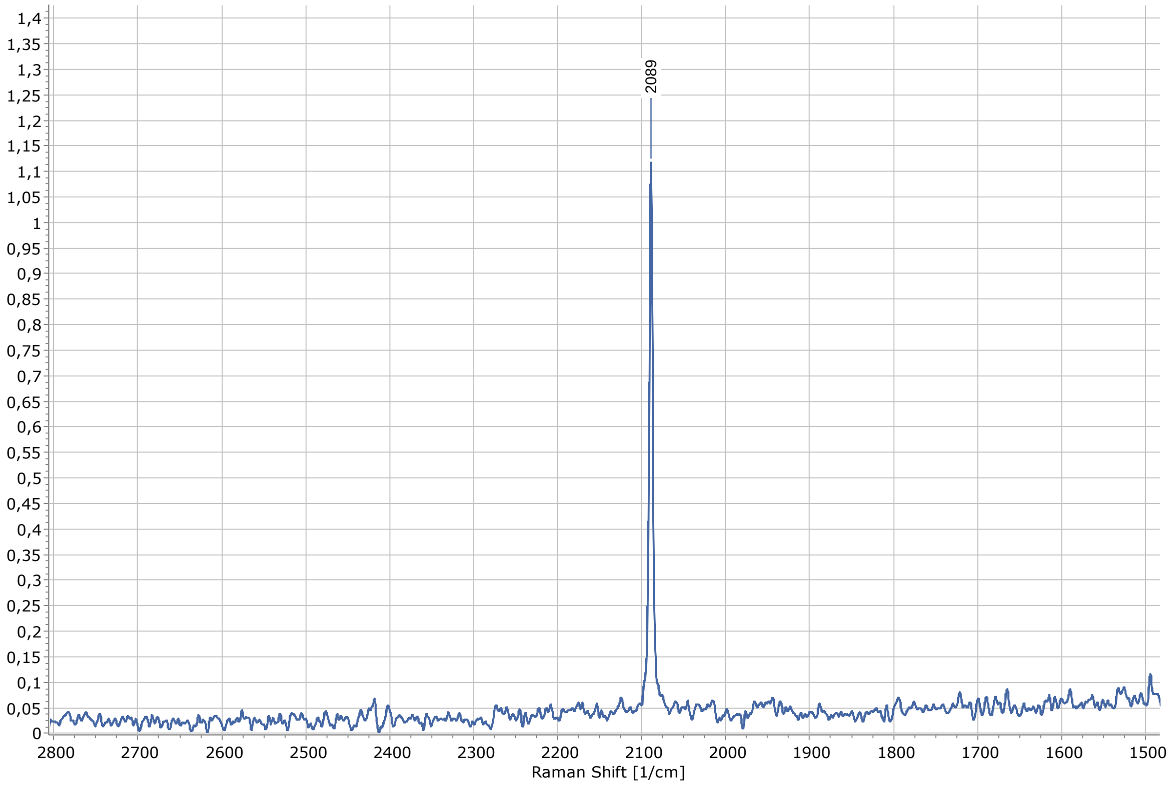

Figure S25-2: Raman spectrum of reference compound $KC^{13}N$:

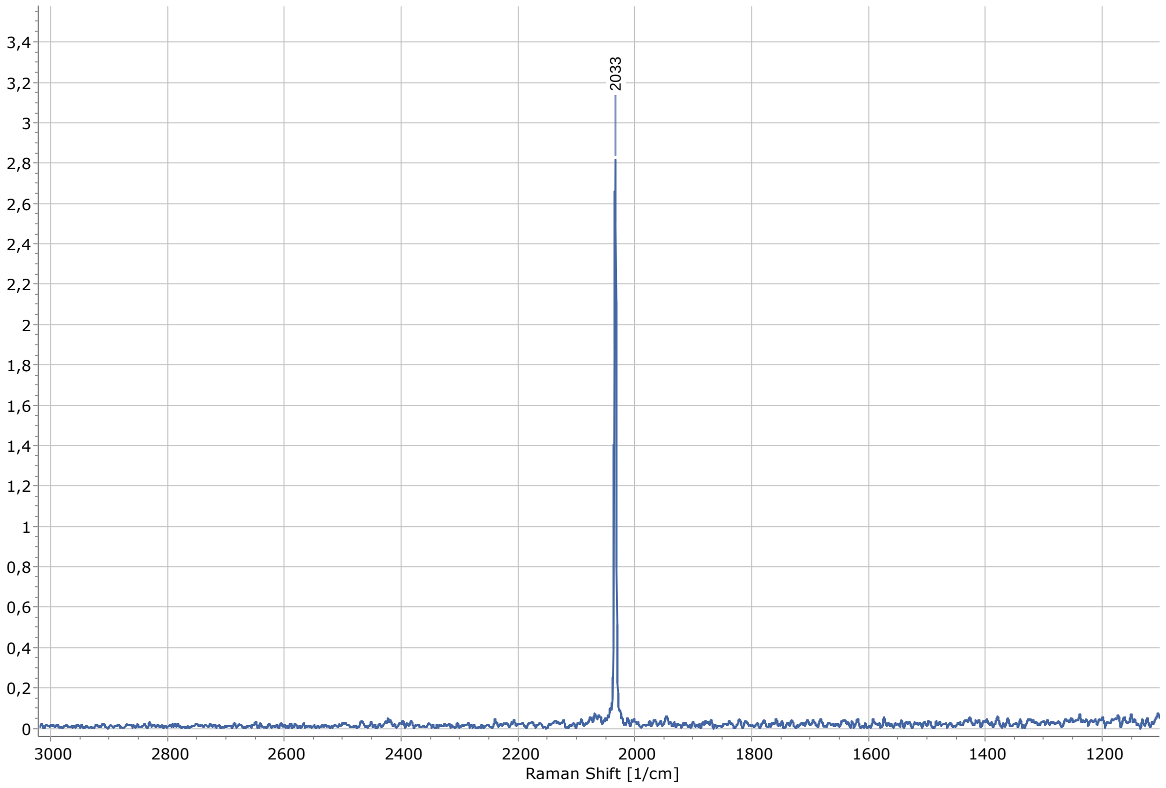

Figure S25-3: Raman spectrum of reference compound Na(1-$^{15}N_3$):

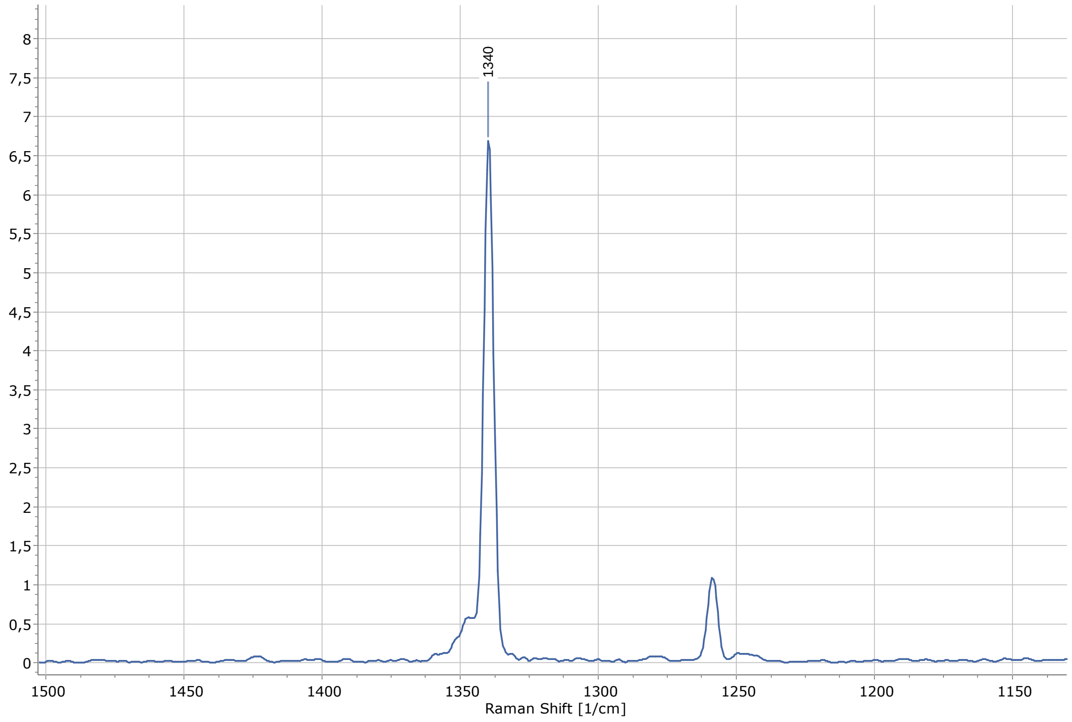

Table S5-1: IR spectroscopic data of NaO*t*Bu, **2-F**, and the sodium alkoxide shell **1**$^{+}$ [Na_{13}(O*t*Bu)$_{12}^{+}$] of compounds **X@1** (X = CH_3, CN, Cl, Br, I, OCN, SCN, N_3, NO_3).

	**NaO*t*Bu**	**CH_3@1**	**CN@1**	**2-F**	**Cl@1**	**Br@1**	**I@1**	**OCN@1**	**SCN@1**	**N_3@1**	**NO_3@1**
ν_{as}(Me)	2956	2950	2950	2949	2951	2950	2949	2949	2949	2950	2950
ν_{s}(Me)	2923										
ν_{s}(Me)	2854	2855	2855	2853	2856	2856	2856	2855	2855	2855	2856
δ_{as}(Me)	1461	1467	1468	1468	1468	1468	1468	1468	1467	1468	1467
δ_{s}(Me)	1376	1373	1373	1371	1373	1373	1372	1373	1372	1373	1372
δ_{s}(Me)	1343	1345	1345	1344	1346	1345	1345	1344	1344	1345	1345
$\delta_{s}(CC_3)+\nu$(CO)	1206	1203	1204	1204	1204	1203	1202	1203	1203	1203	1203
ρ(Me)		1012	1010	1012	1011	1012	1010	1012	1012	1012	1012
ν(CO)	971										
ν(CO)	957	956	958	967	958	958	956	959	960	960	958
ν(CO)			944	948	944	944	944		944		
$\nu_{as}(CC_3)$	872	876	876	875	877	876	876	876	875	876	876
					821	822	821	823		821	
$\nu_{s}(CC_3)$	742	745	745	745	745	745	744	744	744	745	744
	724										
δ(OCC)	495	489	489	491	490	489	489	489	488	488	491
$\delta_{s}(CC_3)$	441	445	445	444	446	445	444	445	445	445	445
$\delta_{as}(CC_3)$		363	363	362		364				362	(361)

Table S5-2: Raman spectroscopic data of NaO*t*Bu, **2-F**, and the sodium alkoxide shell **1**$^{+}$ [Na_{13}(O*t*Bu)$_{12}^{+}$] of compounds **X@1** (X = CH_3, CN, Cl, Br, I, OCN, SCN, N_3, NO_3).

	**NaO*t*Bu**	**CH_3@1**	**CN@1**	**2-F**	**Cl@1**	**Br@1**	**I@1**	**OCN@1**	**SCN@1**	**N_3@1**	**NO_3@1**
ν_{as}(Me)	2948	2954	2955	2954	2955	2955	2954	2954	2953	2954	2954
ν_{as}(Me)	2908										
ν_{s}(Me)	2889	2896	2896	2893	2896	2896	2896	2894	2894	2894	2895
ν_{s}(Me)	2852	2856	2856	2853	2856	2856	2856	2855	2855	2855	2856
	2730	2748	2741	2737	2740	2739	2740	2740	2737	2738	2737
	2671	2679	2678	2673	2680	2678	2678	2677	2676	2677	2677
δ_{as}(Me)	1455	1456	1456	1454	1457	1456	1456	1456	1455	1456	1456
δ_{s}(Me)	1371	1372	1374	1371	1376	1373	1375	1375	1374	1375	1374
δ_{s}(Me)	1344	1346	1350	1346	1350	1374	1346	1346	1346		1347
$\delta_{s}(CC_3)+\nu$(CO)	1223										
$\delta_{s}(CC_3)+\nu$(CO)	1205	1208	1208	1207	1209	1208	1208	1208	1206	1208	1207
ρ_{as}(Me)	1010	1011	1012	1010	1011	1012	1011	1011	1012	1012	1012
ν(CO)	971			999							
ν(CO)	955	955	957	965	956	956	956	959	959	959	956
ν(CO)		945	946	947	944	944	945	947	945	946	947
$\nu_{as}(CC_3)$	872	877	877	875	878	877	876	877	876	877	876
$\nu_{s}(CC_3)$	746	748	749	748	749	748	748	748	747	748	748
		596	611			609	608	611		611	609
δ(OCC)	499	493	495	496	493	494	495	495	493	494	492
$\delta_{s}(CC_3)$	447	449	449	449	450	450	448	449	448	449	449
$\delta_{as}(CC_3)$	360	364	366	363	365	364	364	364	362	364	363

Table S5-3: IR/Raman spectroscopic data of central anions of **CN@1**, **^{13}CN@1**, **OCN@1**, **SCN@1**, **N_3@1**, **1-$^{15}N_3$@1**, **NO_3@1**, **$^{15}NO_3$@1**, relevant data of reference compounds, and results of density functional theory calculations (B3LYP/6-311++G** level of theory), ia inactive; − not observed.

		CN@1	^{13}CN@1	OCN@1	SCN@1	N_3@1	1-$^{15}N_3$@1	NO_3@1	$^{15}NO_3$@1
ν	IR	2079	2036						
	Raman	2080	2037						
	ref.	2089[a]	2033[b]						
	calc.	2122	2078						
ν_{as}	IR			2197	2094	2078	2066	1404	1369
	Raman			2198	2095	ia	ia	-	-
	ref.			2165[c]	2043[d]	2043[e]		1385[g]	
	calc.			2200	2132	2077	2066	1377	1345
ν_s	IR			1303	781	ia	ia	ia	ia
	Raman			1304	780	1350	1330	1074	1074
	ref.			1302[c]	740[d]	1358[e]	1340[f]	1086[g]	1051[h]
	calc.			1250	728	1344	1328	1065	1065
δ	IR			627	-	639	634	-	-
	Raman			629	472	ia	ia	730?	727?
	ref.			628[c]	470[d]	638[e]	628[f]	724[g]	709,721[h]
	calc.			630	459	647	625	709	707
γ	IR							836	815
	Raman							ia	ia
	ref.							−	813[h]
	calc.							835	813

a) NaCN, Raman; b) K^{13}CN, Raman; c) NaOCN[5]; d) KSCN[5]; e) NaN_3[6]; f) Na(1-$^{15}N_3$)[6]; g) $NaNO_3$[7]; h) $NH_4{}^{15}NO_3$, Raman

Theoretical calculation of anion sizes

Table 6-1: Cell volume *V* [Å^3] of compounds **X@1** (X = CH_3, CN, Cl, Br, I, OCN, SCN, N_3, NO_3) based on XRD measurements at 173 K in relation to the corresponding calculated anions sizes [Å^3]. Anion sizes were calculated with Vega ZZ (A. Pedretti, L. Villa, G. Vistoli "VEGA: A VERSATILE PROGRAM TO CONVERT, HANDLE AND VISUALIZE MOLECULAR STRUCTURE ON WINDOWS-BASED PCs", *J. Mol. Graph.* **2002**, *21*, 47-49), based on calculation preformed with Gaussian 09 (B3LYP/6-311++G** level of theory).

Compound	cell volume [Å^3]	anion size [Å^3]
**CH_3@1**	19,4734	26,7
**Cl@1**	19,4387	22,4
**Br@1**	19,4715	26,5
**I@1**	19,5296	32,5
**CN@1**	19,430	27,3
**OCN**	19,3796	33,4
**SCN@1**	19,4555	43,7
**N_3@1**	19,3517	32,3
**NO_3@1**	19,5148	40,5

Figure 26: Graphical representation of Cell volume *V* [Å^3] of compounds **X@1** (X = CH_3, CN, Cl, Br, I, OCN, SCN, N_3, NO_3) based on XRD measurements at 173 K in relation to the corresponding calculated anions sizes [Å^3]. Anion sizes were calculated with Vega ZZ.

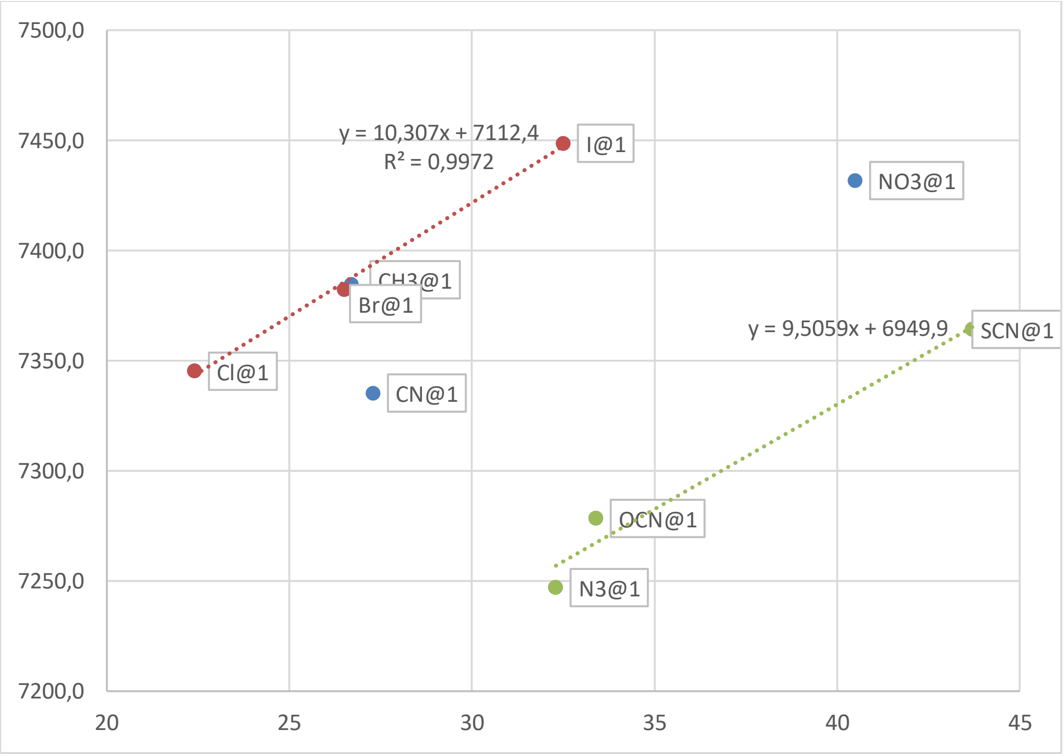

Calculated anion volume [Å^3]

Electrostatical considerations concerning spherical arrangements of point charges

The electrostatic potential of symmetrically distribute point charges was investigated and compared to an electrostatic potential of two point charges. The position of the charges is based on the idealized positions of the disordered sodium atoms and the oxygen atoms of the *tert*-butoxy groups.

The electrostatic potential was calculated with the formula

$$\mathrm{E}(\mathrm{r_i}) = E(r_i) = \frac{1}{4\pi\varepsilon_0}\sum_{i=1}^{n}\frac{q_i}{|r_i|}$$

a) - 20 point charges with a charge of +0.65 units on the corners of a regular pentagon dodecahedron with a distance of 3.3291 Å from the centre (blue).
   - 12 point charges with a charge of -1.00 units on the corners of a regular icosahedron with a distance of 3.7655 Å from the centre.

b) – 2 point charges with a charge of +1.00 units on two opposite corners of a regular pentagon dodecahedron with a distance of 3.3291 Å from the centre (red).

Figure 27-1: two dimensional plot of the electrostatic potentials based on a) (blue) and b) (red).

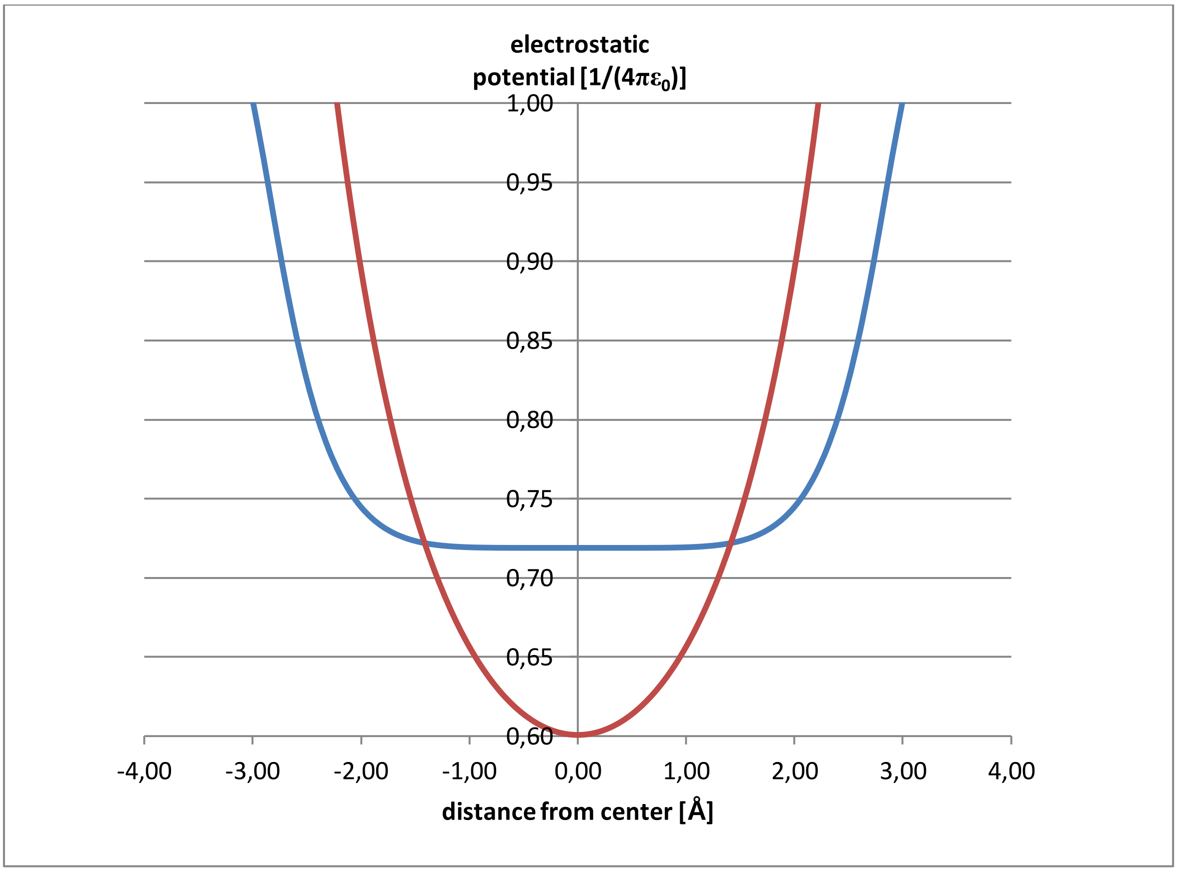

Figure 27-2: three dimensional plot of the electrostatic potentials based on a) with 3d surface plot and contour plot on a plane rectangular to the c_5-axis (left: full plot; right: cut along the c_5-axis).

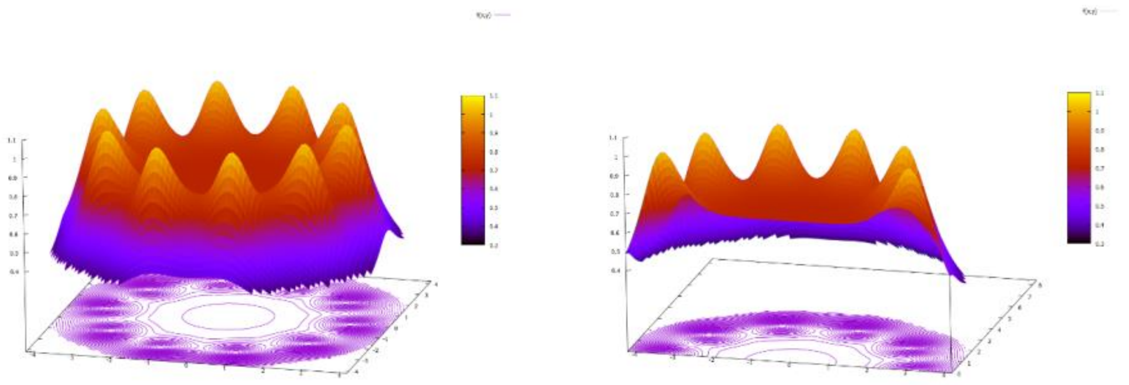

Figure 27-3: left: three dimensional plot of the electrostatic potentials based on a) with 3d surface plot and contour plot on a plane rectangular to the c_2-axis. Right: three dimensional plot of the electrostatic potentials based on b) (two sodium atoms on two opposite positions of a pentagon dodecahedron).

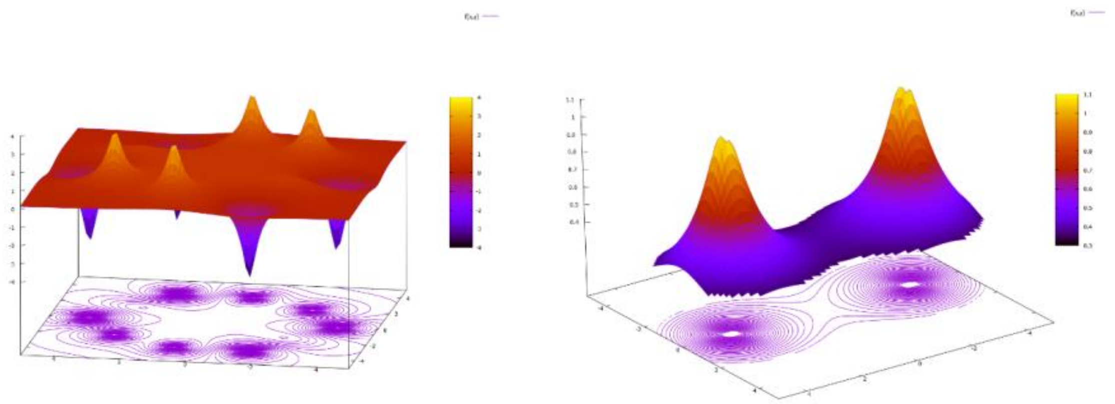

References:

[1] W. Clegg, B. Conway, A. R. Kennedy, J. Klett, R. E. Mulvey, L. Russo, *Eur. J. Inorg. Chem.* **2011**, *2011*, 721.
[2] P. Magnus, G. Roy, *Organometallics* **1982**, *1*, 553.
[3] Y. Tu, J. Zhong, H. Wang, J. Pan, Z. Xu, W. Yang, Y. Luo, *Journal of labelled compounds & radiopharmaceuticals* **2016**, *59*, 546.
[4] K. Shibatomi, Y. Zhang, H. Yamamoto, *Chemistry, an Asian journal* **2008**, *3*, 1581.
[5] S. D. Ross, *Inorganic infrared and Raman spectra*, McGraw-Hill, London, **1972**.
[6] J. I. Bryant, *J. Chem. Phys.* **1964**, *40*, 3195.
[7] D. L. Rousseau, R. E. Miller, G. E. Leroi, *J. Chem. Phys.* **1968**, *48*, 3409.
